# Rapid Measurement of Heteronuclear Coupling Constants in Complex NMR Spectra

**DOI:** 10.1021/jacs.3c05515

**Published:** 2023-08-31

**Authors:** Coral Mycroft, Guilherme Dal Poggetto, Thaís M. Barbosa, Cláudio F. Tormena, Mathias Nilsson, Gareth A. Morris, Laura Castañar

**Affiliations:** † Department of Chemistry, University of Manchester, Oxford Road, Manchester, M13 9PL, United Kingdom; ‡ Chemistry Research Laboratory, Department of Chemistry, University of Oxford, Oxford, OX1 3TA, United Kingdom; § Chemistry Institute, University of Campinas − UNICAMP, P.O. Box 6154, 13083-970 Campinas, SP, Brazil; ∥ Analytical Research & Development, Merck & Co., Inc., 126 Lincoln Avenue, Rahway, New Jersey 07065, United States; ⊥ Nanalysis Corp., 1-4600 5 Street NE, Calgary, Alberta, Canada T2E 7C3; # Department of Organic Chemistry, Faculty of Chemical Science, Complutense University of Madrid, Ciudad Universitaria s/n, 28040 Madrid, Spain

## Abstract

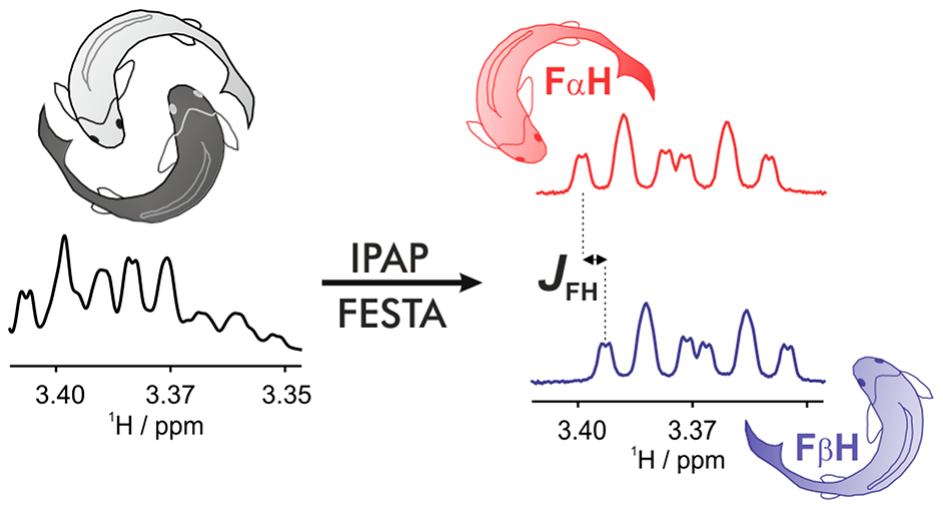

The NMR analysis of fluorine-containing molecules, increasingly widespread due to their importance in pharmaceuticals and biochemistry, poses significant challenges. Severe peak overlap in the proton spectrum often hinders the extraction of critical structural information in the form of heteronuclear scalar coupling constants, which are crucial for determining pharmaceutical properties and biological activity. Here, a new method, IPAP-FESTA, is reported that drastically simplifies measurements of the signs and magnitudes of proton–fluorine couplings. Its usefulness is demonstrated for the structural study of the steroidal drug fluticasone propionate extracted from a commercial formulation and for assessing solvent effects on the conformational equilibrium in a physically inseparable fluorohydrin mixture.

## Introduction

Chemists–especially in the pharmaceutical industry–frequently use the replacement of a hydrogen atom by fluorine to modify the physical, chemical, and biological properties of a molecule.^1−3^ This has the incidental advantage that the very favorable NMR properties of the ^19^F nucleus offer both a wealth of structural information from chemical shifts and coupling constants and a way to increase the resolving power of ^1^H NMR. Here we show that the high resolution of ^19^F NMR can be used to unlock the detailed structural information latent in complex and highly overlapped ^1^H spectra, allowing both signs and magnitudes of heteronuclear scalar couplings to be determined.

^19^F NMR is an attractive complement to ^1^H NMR, as the ^19^F chemical shift is much more sensitive to changes in the local chemical environment, with a signal dispersion range of over 500 ppm. Similarly to homonuclear scalar couplings (*J*_HH_), heteronuclear couplings (*J*_HF_) provide valuable insights into conformation and stereochemistry.^4^ While the magnitudes of *J*_HH_ couplings are typically <25 Hz, it is not unusual to observe much larger magnitudes in *J*_HF_ couplings; ^2^*J*_HF_ magnitudes can exceed 50 Hz. Vicinal ^3^*J*_HF_ couplings are typically larger than ^3^*J*_HH_ couplings, and negative *J*_HF_ couplings are more common than negative *J*_HH_ couplings.^5−8^ Long-range ^*n*^*J*_HF_ couplings, where *n* > 3, are frequently observable, with magnitudes dependent on both *n* and stereochemistry.^9,10^ Determining the signs and magnitudes of *J*_HF_ couplings is crucial for obtaining stereospecific structural information,^10−12^ but is often difficult in conventional 1D ^1^H NMR because of the added complexity of multiplet structure that is caused by both *J*_HH_ and *J*_HF_ couplings.

Heteronuclear couplings can be determined in relatively simple spectra by comparing ^19^F coupled and decoupled ^1^H NMR spectra, provided that the ^1^H signals are reasonably well dispersed. Still, obtaining *J*_HF_ values without performing a complete analysis and, preferably, iterative line shape fitting using spin simulation software is complicated. In complex spectra, it might be challenging to ascertain the hydrogen coupling partners and their respective *J*_HF_ values. Multidimensional experiments can alleviate the effects of signal overlap in 1D NMR spectra and can often allow the determination of *J*_HF_ coupling constants,^10,13−17^ but are very time-consuming when high resolution is required. Several 1D selective methods are available that would enable both the signs and magnitudes of *J*_HF_ couplings to be obtained, but these suffer from low sensitivity because of the use of ^13^C or are unsuitable in the presence of overlapping multiplets, which are common in mixtures.^18,19^ To facilitate the measurement of *J*_HF_ couplings, even for broad unresolved signals and complex multiplets, 1D and 2D NMR methods are often combined with the IPAP approach to reduce spectral complexity.^20−23^ This method relies on acquiring two complementary data sets where multiplets are in-phase (IP) or anti-phase (AP) with respect to heteronuclear couplings. By taking the sum and difference of IP and AP data sets, two IPAP subspectra are generated. *J*_HF_ signs and magnitudes can then be easily obtained by comparing the IPAP subspectra and measuring their displacements, either by overlaying and visual inspection or by least-squares fitting.^24^ Saurí et al.^19^ reported a 1D selective HSQMBC-TOCSY experiment using the IPAP approach, demonstrating fast and easy determination of *J*_HF_ couplings in simple systems. The method requires selective excitation of a well-resolved ^1^H multiplet; as otherwise, the resulting spectrum would contain multiple ^19^F-containing spin systems. Where a ^1^H multiplet is coupled to multiple fluorines, the measured “*J*-coupling” would be a compromise value between all of the *J*_HF_ coupling constants.

Several methods in the literature have been proposed that drastically reduce the spectral complexity in ^1^H NMR spectra, allowing the direct measurement of *J*_HF_ couplings from spectra. Pure shift NMR methods reduce signal overlap in ^1^H spectra by suppressing the effect of homonuclear couplings,^25−28^ allowing heteronuclear couplings to be measured in simple systems.^29−31^ However, most such methods suffer from low sensitivity and, with a couple of exceptions, are not sensitive to the sign of the *J*_HF_ coupling.^32,33^ Alternatively, FESTA (fluorine-edited selective TOCSY acquisition) NMR methods^34,35^ allow simplified ^1^H subspectra to be obtained that show only those protons that are in a spin system coupled to the fluorine of interest, facilitating the measurement of *J*_HF_ values. Two types of FESTA methods are available, differing in how the scalar coupled ^19^F–^1^H pair is excited: selective reverse INEPT (SRI) FESTA and modulated echo (MODO) FESTA. SRI-FESTA^34^ often results in cleaner spectra, whereas MODO-FESTA^35^ is typically more sensitive. Independent of which FESTA method is used, the magnitudes of *J*_HF_ couplings can be obtained by comparing ^19^F coupled and decoupled FESTA spectra, provided that the ^1^H signals are reasonably well dispersed. In both pure shift and FESTA methods, all signals are in-phase with respect to the *J*_HF_ couplings. Therefore, the signs of *J*_HF_ couplings cannot be determined. Combining pure shift methodology with an anti-phase version of FESTA, named HD-HAPPY-FESTA,^33^ allows both the signs and the magnitudes of *J*_HF_ couplings to be measured for the selected spin system. However, like conventional pure shift methods, HD-HAPPY-FESTA suffers from low sensitivity and long experiment times. Furthermore, small *J*_HF_ couplings are difficult to determine if the anti-phase signal line widths are comparable to the *J*_HF_ coupling, as the positive and negative components of the anti-phase signal cancel. This both reduces the signal intensity and causes the measured peak displacement to be greater than the heteronuclear coupling constant.

**Figure 1:**
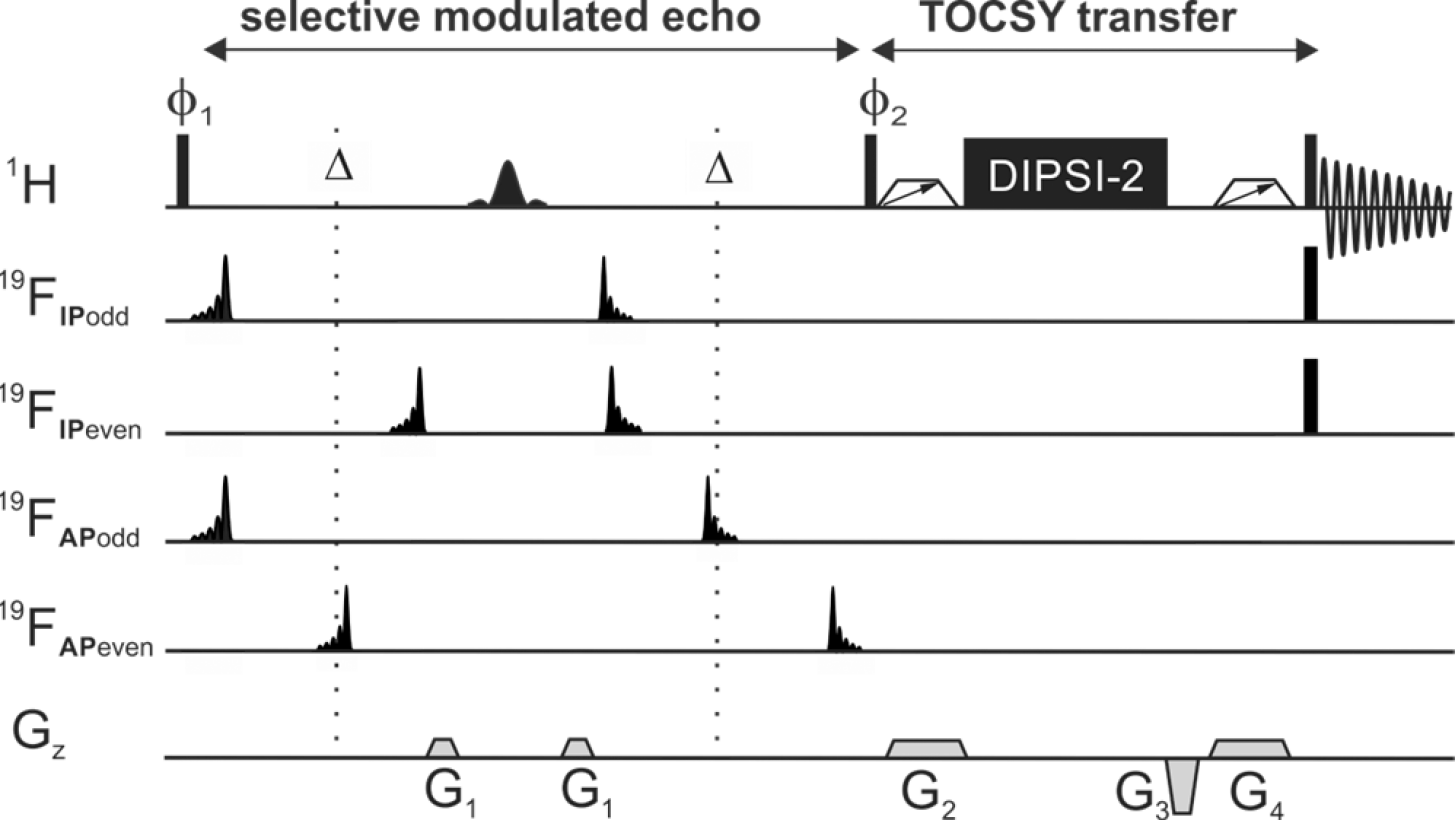
IPAP-FESTA pulse sequence diagram. A multiple of four scans is recorded, with each scan following a different ^19^F channel scheme, acquiring either in-phase (IP) or anti-phase (AP) multiplets. Narrow black rectangles denote hard 90° radiofrequency pulses. Shaped pulses denote ^1^H- and ^19^F-selective 180° pulses applied to the selected ^1^H and ^19^F coupled spins. The vertical dotted lines highlight the center of each half of the selective spin echo. Further details are given in the SI.

**Figure 2:**
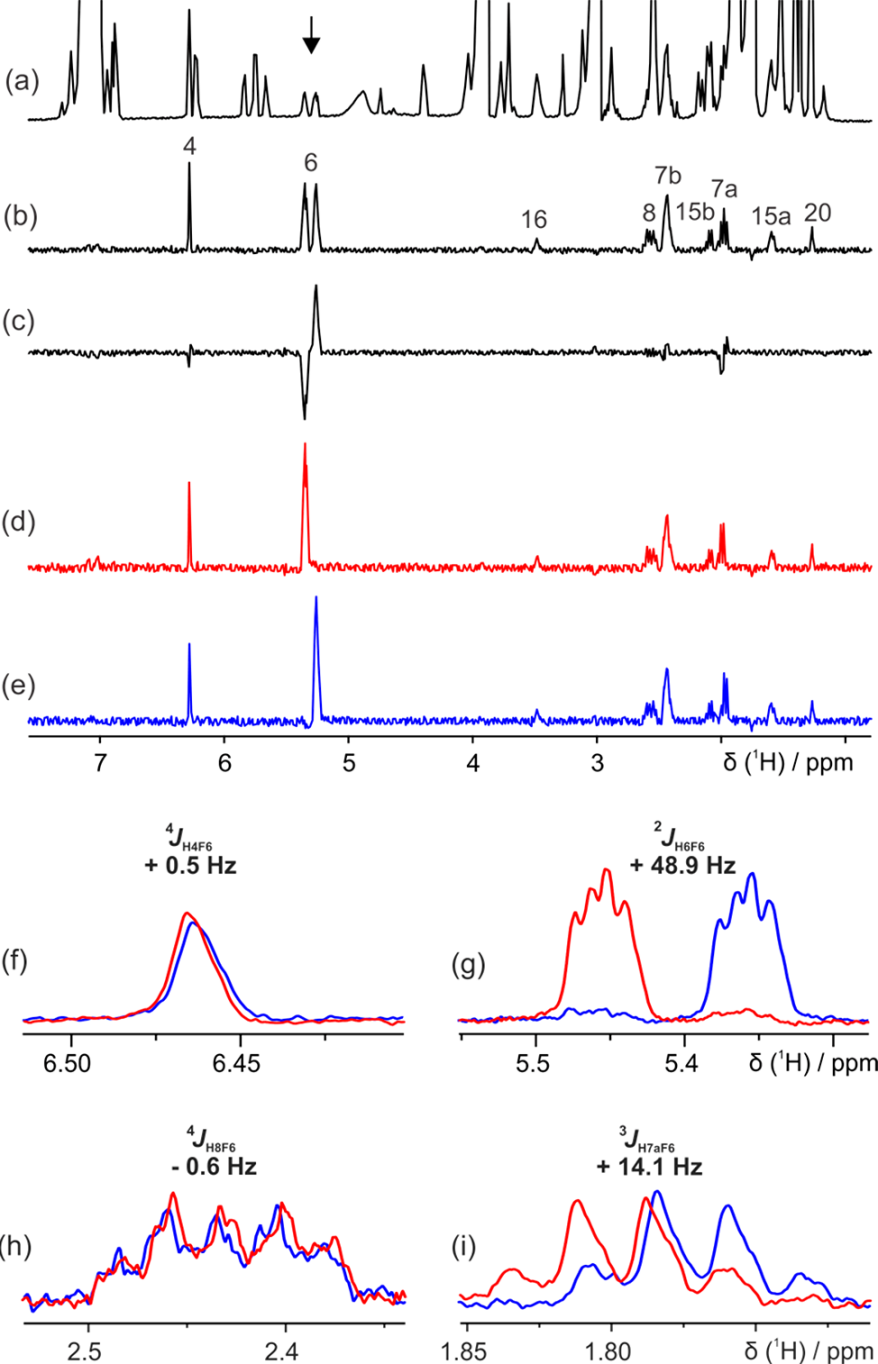
500 MHz ^1^H NMR spectra of crude extracted fluticasone propionate in CDCl_3_: (a) conventional ^1^H NMR, (b) IP-MODO-FESTA, and (c) AP-MODO-FESTA and processed (d) IP-*k*AP and (e) IP+*k*AP MODO-FESTA. (f–i) Expansions of an overlay of (d) and (e). A scaling factor *k* of 1.07 was applied to the AP data when generating spectra (d) to (i). In IPAP-FESTA experiments, the ^1^H signal at 5.4 ppm, indicated by an arrow in (a), and the ^19^F signal at −187.6 ppm were initially selected. Further experimental details are given in the SI.

**Figure 3:**
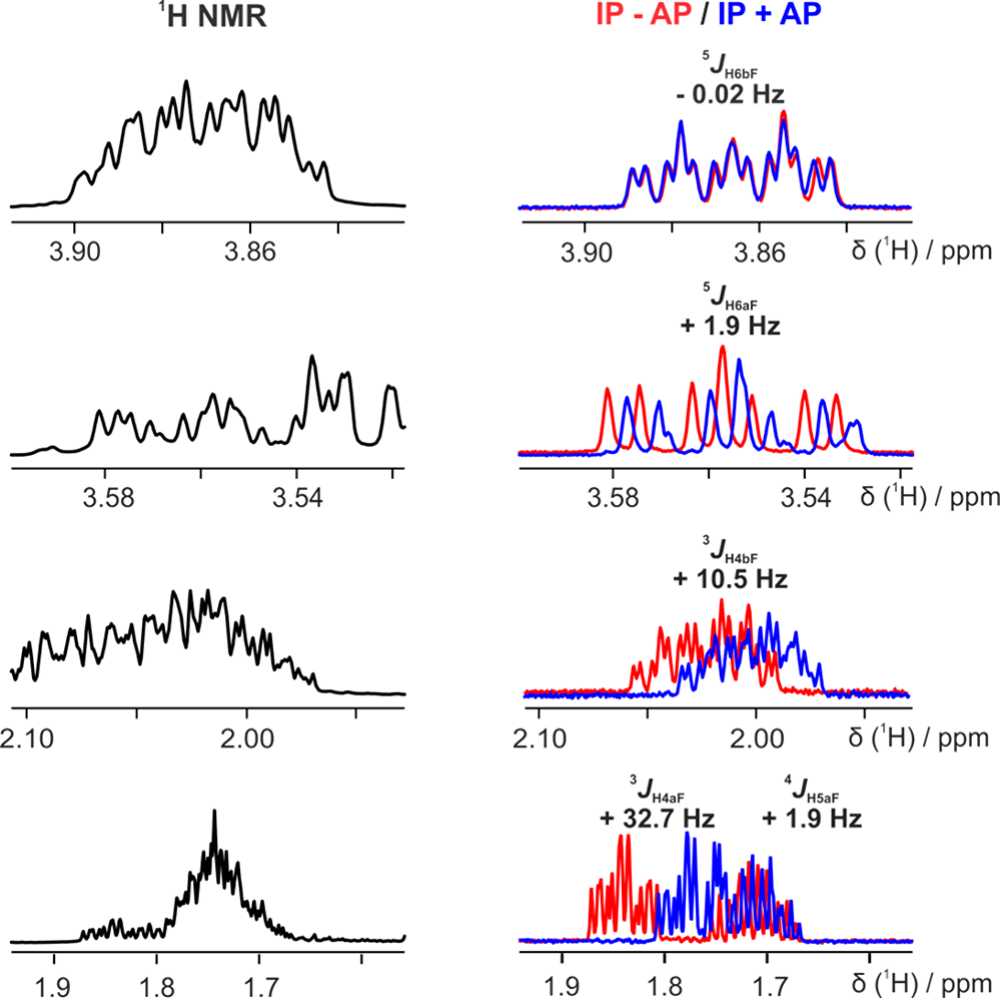
500 MHz ^1^H NMR spectra of fluorohydrin in D_2_O: (left) expansion of the conventional ^1^H and (right) overlay of (IP + *k*AP, blue) and (IP – *k*AP, red) IPAP-FESTA spectra for *cis*-fluorohydrin, for selected proton sites. *J*_HF_ values and peak assignments are shown above each IPAP spectrum pair. Full spectra, and all experimental parameters, can be found in the SI, Section 2b. In proton labels, “a” corresponds to axial and “b” to equatorial.

Here, we propose an experiment that combines the benefits of IPAP^24^ and FESTA^35^ methodologies. The new IPAP-FESTA method (Figure 1) allows the extraction of both the signs and magnitudes of *J*_HF_ couplings as small as 0.1 Hz in as little as 3 min, much more rapidly than HD-HAPPY-FESTA or alternative 2D experiments. Its usefulness is demonstrated in the structural analysis of a crude mixture of fluticasone propionate, extracted from a nasal formulation used to treat asthma and allergic rhinitis.^36,37^ IPAP-FESTA suppresses the signals of the major excipient in the mixture, and aids structure determination, by revealing the signs and magnitudes of all the *J*_HF_ couplings of weakly coupled ^1^H signals within the selected spin system (Figure 2). The chemical utility of the method is also illustrated by experimentally measuring *J*_HF_ values (Figure 3) to investigate solvent effects in a physically inseparable fluorohydrin mixture of two isomers, each with two main conformers in rapid equilibrium. Measured values of *J*_HF_ for the fluorohydrin mixtures are compared to those obtained by density functional theory (DFT) calculations, to determine the relative populations of the different isomers and conformers in three different solvents.

## Experimental Section

### Sample Preparation

The crude mixture of fluticasone propionate and excipients used in this work contained approximately 0.9 mM fluticasone and was extracted using CDCl_3_ (99.9%, Eurisotop) from the Nasofan formulation. The three fluorohydrin mixtures used contained 0.68 M 3-fluorotetrahydro-2*H*-pyran-2-ol in DMSO-*d*_6_ (99.9 atom % D, Sigma-Aldrich), 0.58 M in D_2_O (99.9 atom % D, Sigma-Aldrich), and 0.74 M in CDCl_3_ (99.9 atom % D, Sigma-Aldrich).

### DFT Calculations

All calculations were performed using Gaussian 16 software;^38^ computational details and Cartesian geometries are provided in the SI. The potential energy curves for *cis*- and *trans*-fluorohydrin were scanned at the B3LYP/cc-pVDZ level of theory, and the geometries for the minima in the curves were then fully reoptimized. The Gibbs energy differences for the stable conformers were calculated at the M062X/aug-cc-pVTZ level of theory, in isolated phase and including solvent effects, using the SMD model. The *J*_HF_ couplings for each stable conformer were determined at B3LYP functional level employing the EPR-III basis set for the hydrogen, carbon, and fluorine atoms and the cc-pVDZ basis set for oxygen. The *J*_HF_ couplings calculated for each stable conformer were combined with the conformer ratios to give a weighted average *J*_HF_ coupling for each proton, in the isolated phase and in H_2_O, CHCl_3_, and DMSO.

### NMR Methodology

A schematic representation of the IPAP-FESTA pulse sequence is shown in Figure 1. It can be described as a doubly selective modulated echo followed by a TOCSY transfer, similar to MODO-FESTA.^35^ The resulting spectrum shows only ^1^H signals that are in the same spin system as that of a specific ^19^F site. IPAP-FESTA contains four variations of the modulated echo, resulting in the acquisition of two complementary sets of interleaved IP and AP data. Within each data set, modulated and unmodulated data are acquired by changing the phase of the excitation 90° ^1^H pulse (ϕ_1_ = *x* on odd-numbered scans, ϕ_1_= −*x* on even-numbered scans) and using different arrangements of ^19^F selective iBURP pulses and delays in odd- and even-numbered scans. The delay Δ is set to 1/2*J*_HF_ for A_*n*_X and A_*n*_X_3_ spin systems and 1/4*J*_HF_ for A_*n*_X_2_ spin systems, where *n* is the number of equivalent protons and *J*_HF_ is the heteronuclear coupling constant between the selected fluorine and proton(s). Note that the second iBURP ^19^F pulse is time-reversed with respect to the first, to refocus any *J*_HF_ evolution that occurs during the first pulse.^33,39^ In each data set, identical signals are obtained in odd- and even-numbered scans for the selected protons, while all other protons yield equal but opposite phases and, therefore, are suppressed by summing the two scans. Field gradient pulses flank the selective ^1^H shaped pulse for coherence transfer pathway selection. A hard 90° ^1^H pulse is applied after the modulated echo, with the phase ϕ_2_ of the pulse depending on whether IP (ϕ_2_ = *x*) or AP (ϕ_2_ = −*x*) data are being acquired. For the TOCSY transfer, DIPSI-2 is used as the isotropic mixing element,^40^ transferring the IP or AP magnetization from the selected ^1^H signal to all other spins within the same spin system. Zero-quantum suppression elements are applied on either side of the DIPSI-2 element, together with a purge gradient pulse, to suppress the effects of zero-quantum coherences and of unwanted transverse magnetization.^41^ A final 90°_*x*_^1^H pulse transfers all desired magnetization to the transverse plane for observation. An additional phase-cycled 90° ^19^F pulse is applied simultaneously to the 90° ^1^H pulse in IP-MODO-FESTA, to ensure that any remaining AP contributions are suppressed before data acquisition and to give pure IP signals. The result is that only proton signals from the selected ^1^H–^19^F spin system, IP or AP with respect to the *J*_HF_ coupling to the specified ^19^F survive with any other signals being suppressed. SRI-FESTA^34^ could potentially be used instead of MODO-FESTA; however the higher sensitivity of the latter is advantageous when analyzing dilute mixtures such as the fluticasone propionate mixture described here. Further details are provided in the SI.

### NMR Acquisition and Processing

Fluticasone propionate spectra were recorded at 298 K on a Bruker Avance NEO 500 MHz spectrometer (Bruker Biospin) with a 5 mm BBFO probe with standard transmitter routing and equipped with a z-gradient coil with a maximum nominal gradient strength of 50 G cm^–1^. Fluorohydrin spectra were recorded at 298 K on a Bruker Avance III 500 MHz spectrometer (Bruker Biospin) with a 5 mm BBO probe with standard transmitter routing equipped with a z-gradient coil with a maximum nominal gradient strength of 53.5 G cm^–1^. The durations of the hard ^1^H and ^19^F 90° pulses were calibrated before every sample analysis. iBURP2^42^^19^F-selective and rSNOB^43^^1^H-selective 180° shaped pulses were used. The delay Δ was set to 9.1–11.8 ms, depending on the selected ^19^F–^1^H pair and their corresponding *J*_HF_ coupling. All gradient pulses (G_1_–G_4_) had a smoothed square shape (SMSQ) with 100 digitized points. Gradients G_1_–G_4_ had strengths of 23%, 3%, 79%, and 4%, respectively, as a fraction of the nominal maximum amplitudes given above. Following acquisition, IPAP data were processed using the Bruker “split” AU macro. All raw data, experimental parameters, Mathematica notebooks used for the accurate measurements of *J*_HF_ couplings, and Bruker pulse program codes used in this work are available at DOI: 10.48420/21975908. Further details are provided in the SI.

## Results and Discussion

### Structural Study: Fluticasone Characterization from the Nasofan Formulation

The structural analysis of a crude extract of fluticasone propionate, a pharmaceutical known for its anti-inflammatory properties, was facilitated by IPAP-FESTA.^36,37^

**Scheme 1:**
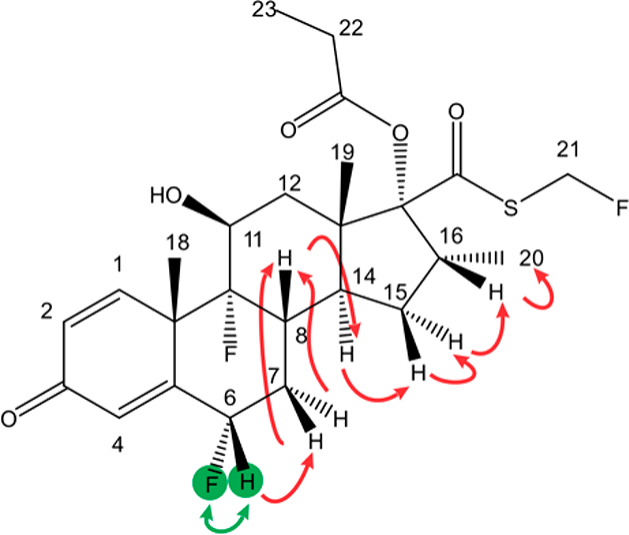
Fluticasone Propionate The green discs and arrow indicate the ^1^H–^19^F coupled pair that was selected, and red arrows represent the ^1^H–^1^H TOCSY transfer.

The extraction process (detailed in the SI) resulted in a dilute mixture (Figure 2a) in which fluticasone propionate is present at approximately 4%, determined by ^1^H NMR, of the concentration of the major component of the extracted formulation, the excipient 2-phenylethan-1-ol. The presence of fluticasone propionate (Scheme 1) was confirmed by the appearance of three distinct signals in the ^19^F NMR, corresponding to F6, F9, and F21 (Figure S2c). Distinct ^1^H signals of fluticasone can be observed in the region 4.0 to 6.5 ppm, but signal overlap below 4 ppm obscures most of the ^1^H signals. Comparison between the conventional ^1^H NMR spectrum and the corresponding ^19^F decoupled spectrum (Figure S3) showed that the ^1^H signal at 5.4 ppm had a coupling to F6 of 48.9 Hz, indicative of ^2^*J*_HF_ coupling. This information was used to set the delay Δ in the MODO element of IPAP-FESTA, to generate IPAP spectra of the proton signals from the spin system that contains F6. The IP/AP-MODO-FESTA ^1^H NMR spectra (Figure 2b and c, respectively) show a significant enhancement in resolution, as all other signals, of solvent, major excipient, or otherwise, are suppressed.

In the AP-MODO-FESTA spectrum (Figure 2c), most of the anti-phase ^1^H multiplets have the same relative phase, indicating that most of the ^1^H–^19^F couplings have the same sign as the large coupling ^2^*J*_H6F6_. However, for signals with a small *J*_HF_ coupling, such as that at 2.43 ppm, it is difficult to determine either the sign or magnitude at this stage. Taking the difference (IP – AP) and the sum (IP + AP) of the IP and AP spectra (with a small extra weighting applied to the AP data to compensate for the slightly greater relaxation losses during the pulse sequence; see below) generates complementary IP – AP and IP + AP subspectra (Figures 2d and 1e). The signs and magnitudes of all *J*_HF_ couplings can be determined directly from the relative displacements of the multiplets in the two subspectra. Approximate values can be obtained by inspection, but for accurate results, iterative fitting was carried out in Mathematica, to determine the exact displacement at which maximum overlap is obtained. Iterative fitting gives excellent precision (see SI, Section 3.5), 1 to 2 orders of magnitude better than fitting by eye, but the principal limitation on the accuracy of the results obtained is, as with all IPAP methods, the requirement for weakly coupled, resolved multiplets.

*J*_HF_ values obtained from IPAP-FESTA have been compared to the *J*_HF_ values of well-resolved signals in the ^1^H NMR spectra of all mixtures described in this work and shown to be accurate (see SI, Section 2c). The H8 and H7a protons of fluticasone (Figure 2h and i) illustrate the power of IPAP-FESTA. In the ^1^H NMR spectrum (Figure 2a), both multiplets lie in regions severely overlapped by excipient and residual solvent signals. IPAP FESTA clearly resolves both signals, and their *J*_HF_ values can be determined (^4^*J*_H8F6_ = −0.6 Hz, ^3^*J*_H7aF6_ = +14.1 Hz). The signs of the *J*_HF_ couplings were determined relative to that of the ^2^*J*_H6F6_ coupling of 48.9 Hz, which was assumed to be positive, as expected for geminal ^2^*J*_HF_ couplings. Here, IPAP-FESTA allows the *J*_HF_ coupling between H8 and F6 to be distinguished from the ^3^*J*_H8F9_ coupling, where conventional methods would not allow this.

It is common for unwanted cross-talk artifacts to be present in IPAP spectra, originating from small differences in peak intensity between the IP and AP data sets.^22^ These differences can originate from strong coupling effects, off-resonance effects, inaccurate *J*_HF_ values leading to a mismatched delta (evolution) delay, and, particularly, differing transverse relaxation between IP and AP coherences (see SI, Section 2d). One example of these artifacts can be observed in Figure 2g; in this case, they do not hinder the extraction of the desired *J*_HF_ values. However, such artifacts should ideally be minimized, as they can interfere with determining *J*_HF_ values in less favorable cases. Applying an arbitrary scaling factor *k* to the AP data before calculating the sum and difference, IP ± *k*AP, minimizes cross-talk difference artifacts.^22^ As with the actual determination of *J*_HF_, *k* can be optimized by eye, either for a complete spectrum or for individual multiplets, but once again, for best results, numerical optimization should be used (see SI, Section 5).

### Conformational Study: Impact of the Solvent on the Conformational Isomerism of Fluorohydrins

**Scheme 2:**
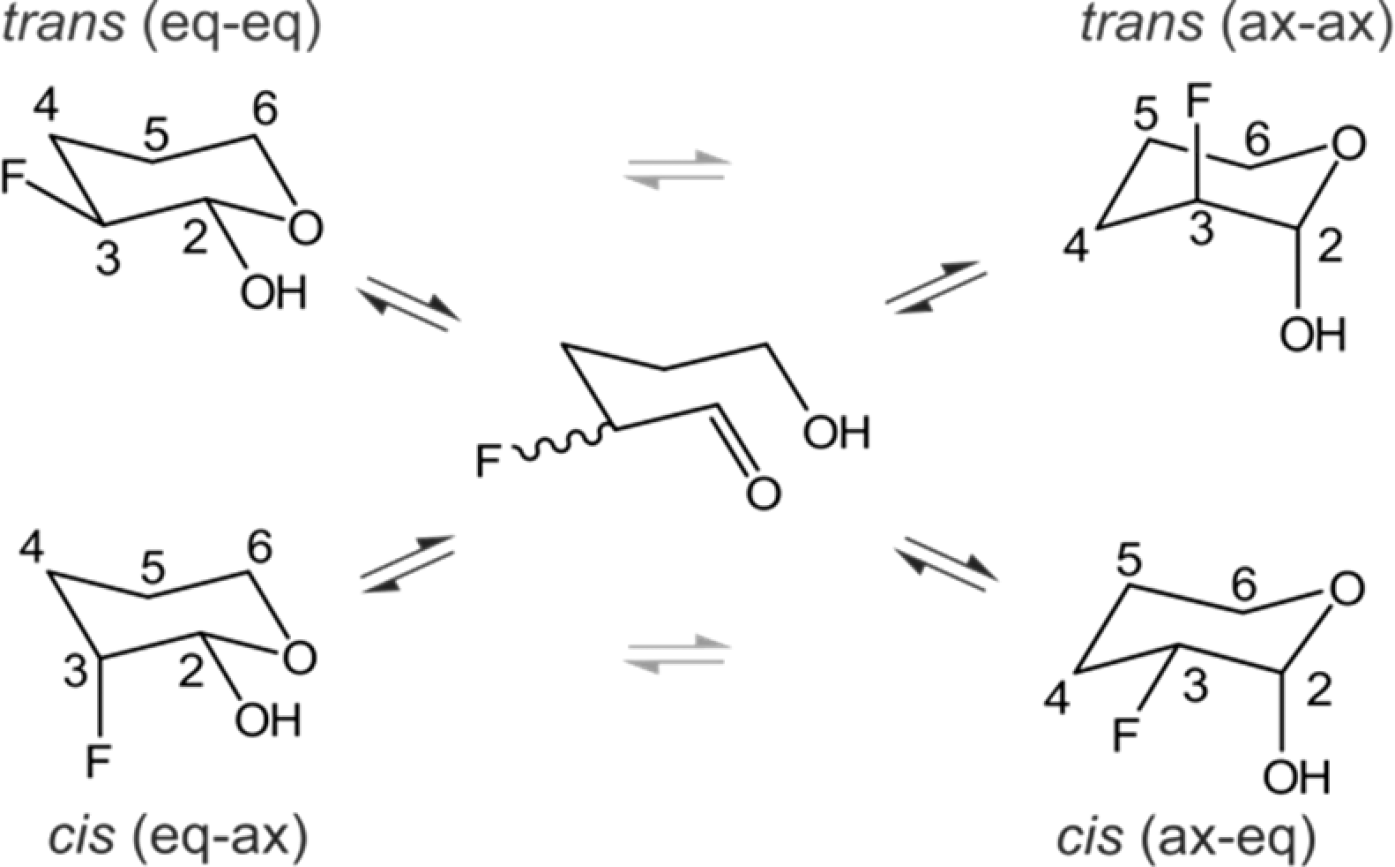
*cis* and *trans* Isomers of 3-Fluorotetrahydro-2*H*-pyran-2-ol (Fluorohydrin), Each with Two Main Conformers The equilibrium between the distinct species is solvent-dependent. Note that the ax–ax conformer has both the F and OH substituents in axial positions, whereas the eq–eq conformer has both substituents equatorial. Grey and black arrows denote equilibration rates on a sub-second and second time scale, respectively.

A further demonstration of the power and usefulness of IPAP-FESTA is provided by a study of solvent effects on the conformational equilibria in a physically inseparable (due to exchange between isomers) mixture of *cis*- and *trans*-fluorohydrins. Fluorohydrins have attracted interest in organofluorine chemistry^44^ as precursors to a range of biologically active fluorinated analogues^45^ and as probes for use as biomarkers.^46^ In solution, 3-fluorotetrahydro-2*H*-pyran-2-ol, referred to here as fluorohydrin, is in a fast conformational equilibrium dominated by eq–eq and ax–ax conformers for the *trans* isomer and by eq–ax and ax–eq conformers for the *cis* isomer (Scheme 2).^47^ Interconversion between the *cis* and *trans* isomers takes place via the ring-opened aldehyde structure. The *cis* and *trans* isomers give rise to separate sets of signals in the ^1^H NMR spectrum, as their interconversion is slow on the chemical shift time scale. However, each isomer shows just one set of signals due to the fast interconversion between conformers; the observed chemical shifts and coupling constants are weighted averages of those for the individual conformers. The ratios between *cis* and *trans* isomers in different solvents were obtained directly by integration of the H3 signal for each isomer from ^1^H spectra (Table 1).

**Table 1:** *cis*/*trans*-Fluorohydrin Isomer Ratios Obtained from ^1^H NMR Spectra (Experimental) and Relative Conformer Percentages Obtained from DFT (Calculated) in Chloroform, DMSO, and Water

	^**1**^H NMR (measured)		DFT (calculated)			
			*cis* conformers		*trans* conformers	
solvent	*cis* isomer	*trans* isomer	ax–eq	eq–ax	ax–ax	eq–eq
chloroform	33%	67%	39%	61%	61%	39%
DMSO	34%	66%	23%	77%	40%	60%
water	40%	60%	19%	81%	18%	82%

**Figure 4:**
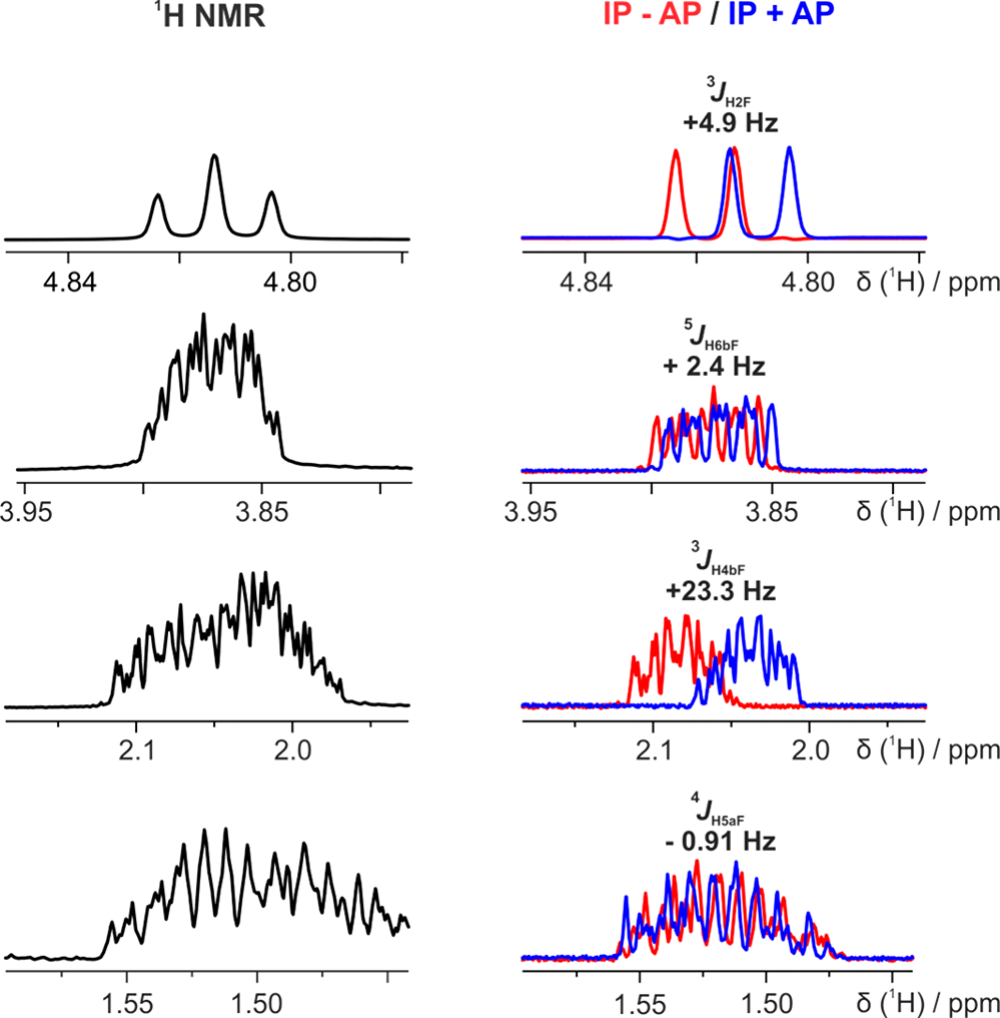
500 MHz ^1^H NMR spectra of fluorohydrin in D_2_O: (left) expansion of the conventional ^1^H and (right) overlay of (IP + *k*AP, blue) and (IP – *k*AP, red) MODO-FESTA spectra for *trans*-fluorohydrin, for selected proton sites. *J*_HF_ values and peak assignments are shown above each IPAP spectrum pair. Full spectra, and all experimental parameters, can be found in the SI, Section 2.2. In proton labels, “a” corresponds to axial and “b” to equatorial.

The relative populations of the four conformers are solvent-dependent, as a consequence of their different intermolecular interactions with solvent. The relative populations of the conformers can be deduced if the scalar coupling constants for all conformers are known, but this is not always the case. Determining the relative populations and, in turn, the solvent effects on them is problematic with conventional NMR methods. The severity of the overlap observed in the ^1^H spectrum of the fluorohydrin mixture can be seen in the expansions at the left of Figures 3 and 4. Analysis of both isomers is almost impossible for signals appearing at 1–4 ppm, as no individual isomer signal is resolved, and there are overlaps with at least one other proton in all three solvents (see SI, Section 2b). Here, the difference in the ^19^F chemical shift (Figure S5) allows the *cis* and *trans* isomers to be separated by FESTA; hence, IPAP-FESTA spectra of both were acquired (Figures 3 and 4, right panel). By comparison between the IP + *k*AP and IP – *k*AP spectra for each isomer, the weighted average *J*_HF_ coupling of the ^1^H signals was obtained (Tables 2 and 3).

**Table 2:** Calculated *J*_HF_ Couplings for *cis*-Fluorohydrin in H_2_O Obtained from DFT and Experimental Couplings in D_2_O Obtained from IPAP-FESTA Spectra^a^

		*J*/Hz			
^*n*^*J*_H(*X*)F_		calculated			
*n*	*X*	*cis* (ax–eq)	*cis* (eq–ax)	weighted average	measured IPAP-FESTA
2	3	54.1	56	55.6	48.5
3	2	0.9	18.3	15.0	14.7
3	4a	6.8	51.7	43.3	32.7^b^
3	4b	9.9	11.5	11.2	10.5
4	5a	4.5	0.7	1.4	1.9^b^
4	5b	–2.2	0.3	–0.2	–0.8^c^
5	6a	4.5	1.2	1.8	1.9
5	6b	–0.4	–0.1	–0.2	–0.02

a In proton labels, “a” corresponds to axial and “b” to equatorial.

b Overlapped signals. *J*_HF_ couplings were measured manually instead of using the Mathematica notebook supplied in the SI.

c *J*_HF_ coupling was measured from the HD-HAPPY-FESTA spectrum due to severe multiplet overlap in both conventional 1D and IPAP-FESTA spectra.

**Table 3:** Calculated *J*_HF_ Couplings for *trans*-Fluorohydrin in H_2_O Obtained from DFT, and Experimental Couplings in D_2_O Obtained from IPAP-FESTA Spectra^a^

		*J*/Hz			
^*n*^*J*_H(*X*)F_		calculated			
*n*	*X*	*trans* (ax–ax)	*trans* (eq–eq)	weighted average	measured IPAP-FESTA
2	3	53	57.6	56.8	48.5
3	2	10.7	3.3	4.7	4.9
3	4a	12.1	13.7	13.4	11.4
3	4b	52	5.8	14.3	23.3
4	5a	–0.1	–2	–1.7	–0.91
4	5b	0.7	3.1	2.7	3.6^b^
5	6a	–0.3	–0.2	–0.2	–0.07
5	6b	1.5	3.7	3.3	2.4

a In proton labels, “a” corresponds to axial and “b” to equatorial.

b *J*_HF_ coupling was measured from the HD-HAPPY-FESTA spectrum due to severe multiplet overlap in both conventional 1D and IPAP-FESTA spectra.

To investigate the effect of solvent on the conformational equilibrium, calculated *J*_HF_ couplings for each stable conformer were required to be compared with the *J*_HF_ values measured from the IPAP-FESTA spectra. The DFT calculations yielded four stable conformers for the *cis* isomer, two each for ax–eq and eq–ax, and five for the *trans* isomer, two for ax–ax, and three for eq–eq, in each case differing in the orientation of the hydrogen of the OH group. The relative conformer populations (Table 1) were calculated using the Gibbs free energy and then grouped together, ignoring the orientation of the hydrogen of the OH group for simplicity.

The weighted average *J*_HF_ coupling for each signal was determined using the conformer-specific *J*_HF_ couplings from DFT calculations and the relative conformer percentages. The calculations, for CHCl_3_, DMSO, and H_2_O, used the SMD implicit solvent approach (see SI, Sections 3.2–3.4),^48^ as the calculation results are largely unaffected by solvent deuteration. It is known from the literature that the use of the implicit approach causes systematic errors in the weighted average calculated scalar coupling values, due to its lack of explicit inclusion of the solvation layer. However, the trends in *J*_HF_ values provide insight into the solvent effects in this system. Calculated DFT and measured IPAP-FESTA *J*_HF_ values in D_2_O are shown in Figures 3 and 4 and are listed in Tables 2 and 3 for *cis*- and *trans*-fluorohydrin, respectively. Further information, including results for CHCl_3_ and DMSO and NMR spectra for CDCl_3_ and DMSO-*d*_6_, is given in the SI.

Overall, there is reasonable agreement between the calculated and measured *J*_HF_ values for all three solvents, with the relative signs of *J*_HF_ couplings consistent and most *J*_HF_ values agreeing within ±1 Hz (Tables S19–S24). Comparison between the IPAP-FESTA and DFT data can be highly informative for obtaining structural information. For example, the long-range ^5^*J*_HF_ couplings between F and H6a and H6b for *cis*-fluorohydrin in D_2_O (Figure 3 and Table 2) allow us to differentiate between them. From DFT calculations, the ^5^*J*_HF_ couplings between F and H6a and H6b are +1.8 and −0.2 Hz, respectively. Experimentally, these ^5^*J*_HF_ couplings are measured to be +1.9 and −0.02 Hz, so they are consistent with the DFT calculations in both sign and magnitude. The same process can be used to differentiate between H6a and H6b in *trans*-fluorohydrin in D_2_O (Figure 4 and Table 3). Examples such as these demonstrate how useful determining the values of long-range couplings can be and highlight the usefulness of determining the sign of a *J*_HF_ coupling as well as the magnitude. It is worth noting that there is a discrepancy between the calculated and measured ^2^*J*_H3F_ values for both *cis*- and *trans*-fluorohydrin in all solvents. This is to be expected, as it is difficult to optimize the basis set and method for long-range and vicinal couplings simultaneously.

To assess the effects of the solvents on the conformational equilibrium, the values for ^3^*J*_H4aF_ in *cis-* and ^3^*J*_H4bF_ in *trans*-fluorohydrin in all three solvents were compared (Table 4), alongside their relative conformer ratios (Table 1). For *trans*-fluorohydrin, the trends in the calculated and measured ^3^*J*_H4bF_ values are in good agreement; as the polarity of the solvent increases, the relative percentage of the eq–eq *trans* conformers increases (Table 1) and the ^3^*J*-coupling decreases. It should be noted that the differences between the calculated and measured ^3^*J*_H4bF_ values increase with the polarity of the solvent, but this is not the case for ^3^*J*_H4aF_ coupling in the *cis* isomer.

**Table 4:** ^3^*J*_H4aF_ Couplings for *cis* and ^3^*J*_H4bF_ for *trans* Isomers of Fluorohydrin in Different Solvents Obtained from DFT Calculations and Experimental IPAP-FESTA Spectra^a^

	isomers			
	*cis*, ^3^*J*_H4aF_/Hz		*trans*, ^3^*J*_H4bF_/Hz	
solvent	DFT	IPAP	DFT	IPAP
chloroform	33.0	26.4^b^	32.8	30.1
DMSO	41.8	18.0	24.1	28.0
water	43.3	32.7	14.3	23.3

a In proton labels, “a” corresponds to axial and “b” to equatorial.

b *J*_HF_ coupling was measured using HD-HAPPY-FESTA data due to severe multiplet overlap.

The calculated values for ^3^*J*_H4aF_ suggest little change between DMSO-*d*_6_ and D_2_O, with values of +41.3 and +43.3 Hz, respectively, but the measured *J*-values for DMSO-*d*_6_ and D_2_O are +18.0 and +32.7 Hz. The discrepancy between the two sets of *J*-values suggests that DMSO interacts stereospecifically with fluorohydrin. This would alter the conformer populations, leading to different average ^3^*J*_FH4a_ couplings when compared to the other solvents, or change the geometry around the site-specific interaction, leading to a change in the transmission of coupling. The disagreement between the calculated and measured *J*-values suggests that the implicit solvent model fails to fully describe solute–solvent interaction in this case.^49,50^ Calculations using explicit solvent (molecular dynamics or quantum dynamics),^51,52^ at increased computational cost, are necessary to describe the studied system adequately, but are beyond the scope of this study.

## Conclusion

The analysis of complex fluorinated mixtures poses an ongoing challenge to ^1^H NMR analysis, with spectra that show both ^1^H–^1^H and ^1^H–^19^F *J*-couplings being particularly difficult to interpret. Here, we have demonstrated how a new method, IPAP-FESTA, can greatly simplify the analysis of mixtures of fluorine-containing species by extracting separate subspectra for the α and β spin states of a single heteronuclear spin-1/2, in as little as 3 min. This allows both signs and magnitudes of heteronuclear couplings to be determined, even when overlapping signals completely obscure the resonances in the conventional ^1^H spectrum. The reduction in spectral overlap and the accurate measurement of *J*_HF_ couplings aid the structural analysis of even the most complex mixtures. It is also shown that IPAP-FESTA can be used alongside DFT calculations to determine conformer ratios in multiple solvents. FESTA methods are not limited to the analysis of fluorine-containing compounds and can be applied to other spin-1/2 heteronuclei, such as ^31^P or ^29^Si.

## Supporting Information Available

Experimental data, pulse programs, and relevant Mathematica notebooks (PDF)
