# Supplementary material for: Rapid Measurement of Heteronuclear Coupling Constants in Complex NMR Spectra
Source: J Am Chem Soc. 2023 Aug 31;145(36):19824–31. doi: 10.1021/jacs.3c05515 (PMC10510310; doi:10.1021/jacs.3c05515)
Supplement: Supplementary file 1 — ja3c05515_si_001.pdf [file ja3c05515_si_001.pdf]

Rapid measurement of heteronuclear coupling constants in complex NMR spectra

Supporting Information

Coral Mycroft,[a,b] Guilherme Dal Poggetto,*[c,d] Thaís M. Barbosa,[c,e] Cláudio F. Tormena,[c] Mathias Nilsson,[a] Gareth A. Morris,[a] Laura Castañar*[a,f]

[a] Department of Chemistry, University of Manchester, Oxford Road, Manchester, M13 9PL, United Kingdom.

[b] Chemistry Research Laboratory, Department of Chemistry, University of Oxford, Oxford, OX1 3TA, United Kingdom.

[c] Chemistry Institute, University of Campinas – UNICAMP, P.O. Box. 6154, 13083-970 Campinas, SP, Brazil.

[d] Analytical Research & Development, Merck & Co., Inc., 126 Lincoln Ave, Rahway, NJ, USA.

[e] Nanalysis Corp., 1-4600 5 street NE, Calgary, Alberta, Canada T2E 7C3.

[f] Department of Organic Chemistry, Faculty of Chemical Science, Complutense University of Madrid, Ciudad Universitaria s/n, 28040 Madrid, Spain.

laura.castanaracedo@manchester.ac.uk/ lcastana@ucm.es
guilherme.dal.poggetto@merck.com

Contents

1. Experimental section ... 3

1.1. IPAP-FESTA pulse sequence ... 3

1.2. Using Wavemaker to generate shaped pulses ... 5

1.3. Recommended experimental protocol ... 5

1.4. Processing of IPAP-FESTA data using TopSpin ... 5

1.5. Processing of IPAP-FESTA data using Mathematica ... 6

1.6. Computational procedures ... 6

2. Sample preparation and experimental data ... 7

2.1. Fluticasone propionate ... 7

2.2. Fluorohydrin ... 9

2.3. Comparison of J_{HF} couplings measured using IPAP-FESTA and using ^{1}H and $^{1}H\{^{19}F\}$ NMR spectra ... 20

2.4. Cross-talk artifacts and the scaling factor *k* in IPAP data ... 20

3. Computational data and measured IPAP-FESTA data ... 21

3.1. Fluticasone propionate J_{HF} values ... 21

3.2. Potential energy curves for *cis*- and *trans*- fluorohydrin ... 22

3.3. Fluorohydrin conformer populations determination from Gibbs free energy ... 26

3.4 J_{HF} values from DFT calculations and values extracted from experimental IPAP-FESTA data ... 28

3.5. Repeatability of J_{HF} couplings measured from experimental IPAP-FESTA data ... 32

4. Product operator description of the MODO element in IPAP-FESTA ... 33

5. Mathematica code for determining J_{HF} coupling constants from IPAP-FESTA data ... 34

6. Pulse program codes for Bruker spectrometers ... 36

6.1. IPAP-MODO-FESTA pulse program suitable for a TBI probe ... 36

6.2. IPAP-MODO-FESTA pulse program suitable for a BBFO probe ... 42

6.3. Selective fluorine reverse INEPT (SRI) pulse program suitable for a TBI probe ... 48

7. References ... 50

1. Experimental section

1.1. IPAP-FESTA pulse sequence

The detailed pulse sequence for Bruker implementation is shown in Figure S1. Narrow and wide filled rectangles represent hard 90° and 180° pulses, respectively. The durations of the hard 90° pulses (p1) for each sample are given in Section 2. The two selective 180° iBURP shaped pulses (with duration p14) and the 180° rSNOB shaped pulse (with duration p12) were used to select the specified ^{19}F and ^{1}H coupled spins, respectively. These pulses were generated using Wavemaker (see Section 1e). If only one ^{19}F resonance is present, the selective ^{19}F 180° pulses can be replaced by hard ^{19}F 180° pulses, as heteronuclear selectivity is not required.

TOCSY transfer was achieved using the DIPSI-2 isotropic mixing scheme with a mixing time (d9) of 100-150 ms dependent on the spin system being analysed. The trapezoids on either side of the DIPSI-2 element indicate low-power 180° chirp pulses used to suppress the effects of zero quantum coherences. Their durations were set to 10 and 30 ms, respectively. The field gradient pulse labelled G_1 in the pulse sequence is used to select the desired coherence transfer pathway, with an amplitude of 23 % of the maximum gradient strength and a duration of 1 ms. Gradient pulse G_3 is a homospoil gradient with a duration of 1 ms and amplitude of 79 %, to dephase any transverse magnetization. G_1 and G_3 have a smoothed square shape (gpnam1 = gpnam2 = SMSQ10.100). Gradient pulses G_1 and G_3 are followed by recovery delays (d16 and d18) of 200 µs. Gradient pulses G_0 and G_{10} are applied during chirp pulses to suppress the effects of zero-quantum coherences and are followed by recovery delays of 100 µs. The delays Δ_1 (equal to half of the delay Δ detailed in the main manuscript) are set to $1/(4J_{HF})$ for A_nX and A_nX_3 spin systems, and $1/(8J_{HF})$ for A_nX_2 spin systems, where *n* is the number of equivalent protons, and J_{HF} is the heteronuclear coupling constant between the specified proton and fluorine. The recommended minimum phase cycle is 16 steps; the full phase cycle is given in Table S1.

Table S1: Phase cycle for IPAP-FESTA described in Figure S1.

**Φ_1**	$x_8, -x_8$
**Φ_{11}**	$-x$
**Φ_2**	$x_2, y_2, -x_2, -y_2$
**Φ_3**	x
**Φ_4**	$x_{16}, -x_{16}$
**Φ_5**	$y_{16}, -y_{16}$
**Φ_6**	x
**Φ_7**	$x_{32}, -x_{32}$
**Φ_8**	$x, -x, -x, x$
**Φ_{23}**	$-y$
**Φ_{25}**	y
**Φ_{31}**	$(x_2, -x_2)_2, (-x_2, x_2), (x_2, -x_2), (-x_2, x_2)_2, (x_2, -x_2), (-x_2, x_2)_3, (x_2, -x_2), (-x_2, x_2), (x_2, -x_2)_2, (-x_2, x_2), (x_2, -x_2)$

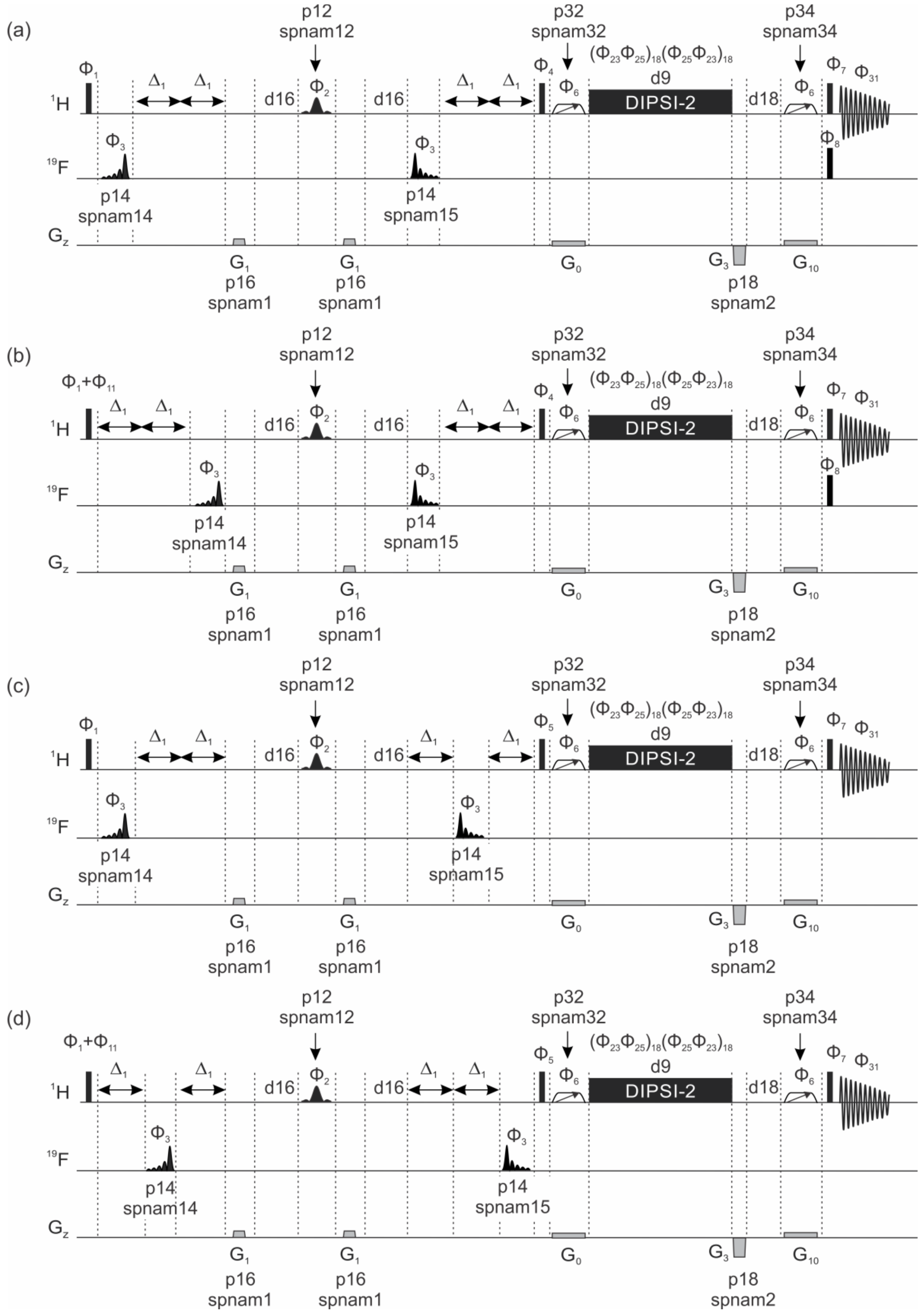

Figure S1: Detailed pulse program for IPAP-FESTA for Bruker implementation, where (a) and (b) are for IP-MODO-FESTA scans and (c) and (d) are for AP-MODO-FESTA scans. The definition of each parameter can be found at the end of the pulse sequence, along with recommended standard values. Modulated and unmodulated data are acquired by changing the phase of the excitation 90° ^{1}H pulse; φ_1= x on odd-numbered scans, φ_1= –x on even-numbered scans.

1.2. Using Wavemaker to generate shaped pulses

Wavemaker is a pulse shaping function within TopSpin® that allows shaped pulses to be generated without extensive manual set-up by the user. *Wavemaker* reads the relevant lines within the pulse program, and creates the shaped pulses required from the specified parameters. In this pulse sequence, there are 3 shaped pulses designed by *Wavemaker*:

- An rSNOB shape (sp12, channel F1) to be used as the ^{1}H 180° refocusing pulse.
- Two inversion iBURP pulses (sp14 and sp15, channel F2) to invert the specified ^{19}F resonance.

The lines in the pulse program defining the *Wavemaker's* parameters are shown below:

```
;sp12:wvm:IPAP_rsnob:f1 rsnob(cnst12 Hz, cnst13 ppm; PA=0.5; NPOINTS=5000) ss=1 us;
;sp14:wvm: IPAP_Fiburp1:f2 iburp2(cnst14 Hz, cnst15 ppm; PA=1; NPOINTS =5000) ss=1 us;
;sp15:wvm: IPAP_Fiburp2:f2 iburp2(cnst14 Hz, cnst15 ppm; TR=1; NPOINTS =5000) ss=1 us;
```

Wavemaker reads the parameters 'cnst12' (^{1}H) and 'cnst14' (^{19}F) to define the effective bandwidths in Hz. For ^{1}H, the effective bandwidth must include only one proton resonance, that is coupled to the specified ^{19}F, and no other ^{1}H coupled partners, in order to prevent J_{HH} modulation. 'cnst13' (^{1}H) and 'cnst15' (^{19}F) specify the chemical shifts at which the selective pulses are to be applied. To generate the shaped pulses, the command '*wvm -a*' must be used following any changes to the parameters.

Wavemaker is automatically downloaded as part of the current standard TopSpin® installation. Any *Wavemaker* updates are available in the Bruker User Library. Alternatively, each shaped pulse can be set up manually.

1.3. Recommended experimental protocol

1. Acquire ^{1}H, ^{1}H {^{19}F}, ^{19}F and ^{19}F{^{1}H} NMR spectra.
2. Acquire SRI (optional) to determine the number of ^{1}H coupled to the chosen ^{19}F, approximate $J_{HF,}$ and their chemical shifts. For more information, refer to the original SRI-FESTA publication.[1]
3. Use the parameters determined in step two to set up the MODO element of IPAP-MODO-FESTA.
    a. Set ZGOPTNS in the Acquisition window ('ACQPARS') to '-DIPAP' and the acquisition dimension to '2D'. To acquire only the IP or AP MODO-FESTA spectrum, set ZGOPTNS to '-DIP' or '-DAP' respectively, and the acquisition dimension to '1D'.
    b. Choose an appropriate DIPSI-2 duration (30 – 200 ms) for the TOCSY transfer to observe the full spin system under study.
    c. Set up the zero quantum filter (ZQF) chirp pulses.[2]
4. Acquire the 2D IPAP-MODO-FESTA experiment to obtain IP and AP spectra of the ^{1}H NMR spectra which only contain protons that belong to the chosen ^{19}F-^{1}H spin system.

1.4. Processing of IPAP-FESTA data using TopSpin

Below is the recommended procedure for processing IPAP-FESTA data within TopSpin version 3.1.6. Minor modifications may be required to process IPAP-FESTA data in other versions of TopSpin.

1. Extract the 1D IP and AP-FESTA spectra from the 2D data set. For example, use the command 'ft 1 1001' to extract the 1st data set (IP-FESTA) to processing number 1001, and the command 'ft 2 1002' to extract the 2nd data set (AP-FESTA) to processing number 1002. Ensure that the IP- and AP-FESTA spectra used identical processing parameters (for example, SI, weighting functions, etc.)
2. Overlay the extracted IP- and AP-FESTA spectra. Determine an appropriate scaling factor *k* for the AP spectrum to ensure minimum cross-talk artifacts in the resulting IPAP spectra.
3. For addition/subtraction processing, use the '*split*' macro to obtain the IP+AP and IP-AP spectra.
    a. The macro will prompt the user to input a scaling factor (*k*) to be applied to the AP-FESTA spectra.
    b. Note that the data may be processed differently depending on the system and '*split*' macro version used.
    c. Measure the signs and magnitudes of J_{HF} using the sign and direction of displacement of the $^{19}F_{\alpha}$ and $^{19}F_{\beta}$ coupled ^{1}H signals. Note that only the relative signs of J_{HF} values can be obtained, not absolute signs. $^2J_{HF}$ couplings are, however, usually large and positive and can be used as a reference.

1.5. Processing of IPAP-FESTA data using Mathematica

A Mathematica® notebook is supplied (see Section 5) to extract the IP and AP-FESTA spectra, apply an optimised scaling factor *k* (between 0.8 and 1.2) to each specified J_{HF} coupled ^{1}H signal, and accurately extract the signs and magnitudes of J_{HF} couplings. The default scaling factor (*k*) range of 0.8 - 1.2 is typically sufficient for correcting relaxation loss in small molecules, but can be modified if required (see below).

The Mathematica notebook requires 7-9 inputs from the user:

- The scaling factor (*k*) range to be applied to the AP-MODO-FESTA spectra ($0.8 < \mathit{fratio} < 1.2$). This range is stated in two lines of code starting with '*res1*' and '*res2*'.
- A comma-separated list of the centres in ppm of the regions to be fitted (*shifts).*
- A comma-separated list of the widths in ppm of the regions to be fitted (*widths).*
- Number of consecutive FESTA experiments (integer value, typically 1) (*nreps).*
- First experiment number (EXPNO - *filestart*).
- File path where experimental data are stored (*filenamebase*).
- Processed IP experiment number (*procnoIP*) generated when processing IPAP-FESTA data using TopSpin (e.g. 1001).
- Processed AP experiment number (*procnoAP*) generated when processing IPAP-FESTA data using TopSpin (e.g. 1002).

See below for an example of the inputs required:

```
res1=NMinimize[{vardiff[tspip,tspap,fratio,5 fdiff],fdiff>-len/5,fdiff<len/5,fratio>0.8,fratio<1.2},{fdiff∈ Integers,fratio},MaxIterations->100];
res2=NMinimize[{vardiff[tspip,tspap,fratio, fdiff],fratio>0.8,fratio<1.2,fdiff>Round[(5 fdiff/.res1[[2]])]-len/10,fdiff<Round[len/10+5 fdiff/.res1[[2]]]},{fdiff∈ Integers,fratio},MaxIterations->1000];

shifts={5.5,4.2};
widths={0.3,0.2};
nreps=1;
filestart=10;
filenamebase="W:/FILEPATH/";
procnoIP="1001";
procnoAP="1002";
```

Running the notebook should extract all the J_{HF} coupling constants from the raw data, as well as the corresponding scaling factors *k* and root mean squared (RMS) uncertainties.

1.6. Computational procedures

The conformational preferences for *cis*- and *trans*-3-fluoro-2-hydroxytetrahydropyran diastereomers (Scheme S2) were computationally evaluated by varying the orientation of the O-H group relative to the six-membered ring system. The potential energy curves were scanned at the B3LYP/cc-pVDZ level of theory, varying the C_3-C_2-O-H dihedral angle from 0 to 360° in steps of 10° for the four significant conformers (Figure S2). For each of the conformers the C_3-C_2-O-H dihedral angle was fixed and the rest of the molecule was allowed to relax during the geometry optimization calculations.

The geometries for the minima in the curves were then fully reoptimized and the Gibbs energy differences for the stable conformers were calculated at the M062X/aug-cc-pVTZ level of theory, in isolated phase (xyz cartesian coordinates listed below), and including solvent effects ($CHCl_3$, DMSO and H_2O) using the SMD model. The Gibbs energies for the stable conformers are listed in Section 3c, while calculated values for the $^{n}J_{FH}$ coupling constants for the most stable conformers were obtained at the B3LYP functional level employing the EPR-III basis set for the hydrogen, carbon, and fluorine atoms and cc-pVDZ basis set for oxygen. The EPR-III basis set was not used for oxygen to save computational time as no couplings involving ^{17}O were calculated or measured. All calculations were performed in the Gaussian 16 program.[3]

2. Sample preparation and experimental data

For fluticasone propionate, assignments were determined using IPAP-FESTA with the aid of ^{1}H-^{1}H COSY, edited ^{1}H-^{13}C HSQC, and ^{1}H-^{13}C HMBC spectra (data not shown) on the pure material. For fluorohydrin in all solvents, assignments were determined using IPAP-FESTA with the aid of ^{1}H-^{1}H COSY, edited ^{1}H-^{13}C HSQC, ^{1}H-^{1}H TOCSY, ^{1}H-^{13}C HMBC and ^{1}H-^{1}H NOESY spectra (data not shown).

2.1. Fluticasone propionate

The compound was extracted from Nasofan^{TM} Aqueous Fluticasone Propionate formulation (product: 87159-E, 4111405-02, batch: 100015299). Deuterated chloroform ($CDCl_3$, 100.0%, Eurisotop, 1.5 mL) and deuterated water (D_2O, 2 mL) were added to the formulation, which was vortexed at 700 min^{-1} for 1 min. The mixture was allowed to settle for 1 h, then the $CDCl_3$ was transferred to an NMR tube for analysis. The extracted mixture contained 0.9 mM fluticasone.

Fluticasone spectra were recorded at 298 K on a Bruker Avance NEO 500 MHz spectrometer (Bruker Biospin) with a 5 mm BBFO probe with standard transmitter routing, equipped with a z-gradient coil with a nominal maximum gradient strength of 50 G cm^{-1}, operating at 500.1 MHz and 470.6 MHz for ^{1}H and ^{19}F, respectively. Data were processed using the software TopSpin (version 4.0.9, Bruker BioSpin). Figure S3 was acquired at 298 K on a Bruker Avance NEO 500 MHz spectrometer (Bruker Biospin) with a 5 mm TBI probe with standard transmitter routing, equipped with a z-gradient coil with a nominal maximum gradient strength of 67 G cm^{-1}, operating at 500.1 MHz and 470.6 MHz for ^{1}H and ^{19}F, respectively.

For ^{1}H NMR experiments, the ^{1}H spectral window was set to 5000 Hz (10 ppm), the carrier frequency to 1900 Hz (3.80 ppm), and the hard 90° pulse duration to 9.75 µs. 1D ^{1}H NMR data were acquired with 64 transients, a time domain data length (TD) of 16384, and an experiment time of 5 min. Prior to Fourier transformation of all 1D ^{1}H NMR data, zero-filling to 65536 complex points was applied, and weighting for a Lorentzian broadening LB of 1 Hz. For ^{19}F experiments, the ^{19}F spectral window was set to 27778 Hz (59.04 ppm), the carrier frequency to –84706 Hz (–180 ppm), and the duration of the hard 90° pulse to 15 µs. 1D ^{19}F NMR data were acquired with 128 transients, a TD of 65536, and an experiment time of 7 min. {^{1}H}^{19}F NMR spectra were acquired using the CPD sequence WALTZ16 with a pulse duration of 80 µs and power level of 0.44 W. Prior to transformation of 1D ^{19}F NMR data, zero-filling to 262144 complex points was applied, and Gaussian weighting with LB of –0.01 Hz and GB of 0.005 was used.

^{1}H IPAP-FESTA spectra were recorded with 2048 transients in an experiment time of 4 h 23 min. The ^{1}H signal at 5.41 ppm was selected using an rSNOB shaped pulse (4.1 ms, 450 Hz effective bandwidth) and the ^{19}F signal at –187.4 ppm was selected by using iBURP shaped pulses (2.8 ms, 1650 Hz effective bandwidth). These shaped pulses were designed using *Wavemaker*. A 100 ms mixing period was used for the DIPSI-2 (d9) element. When generating IPAP-FESTA by addition/subtracting processing, a scaling factor (*k*) of 1.072 was applied to the AP spectra.

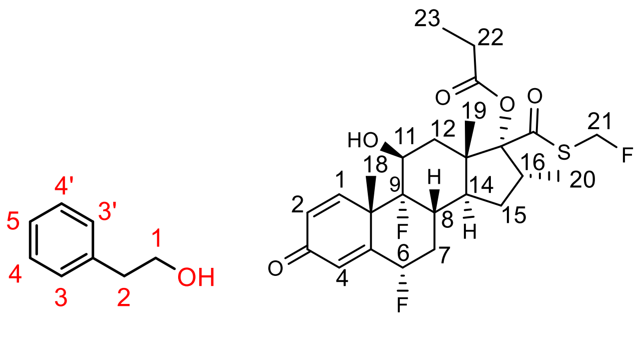

Scheme S1: Molecular structures of major excipient 2-phenylethanol (left) and fluticasone propionate (right).

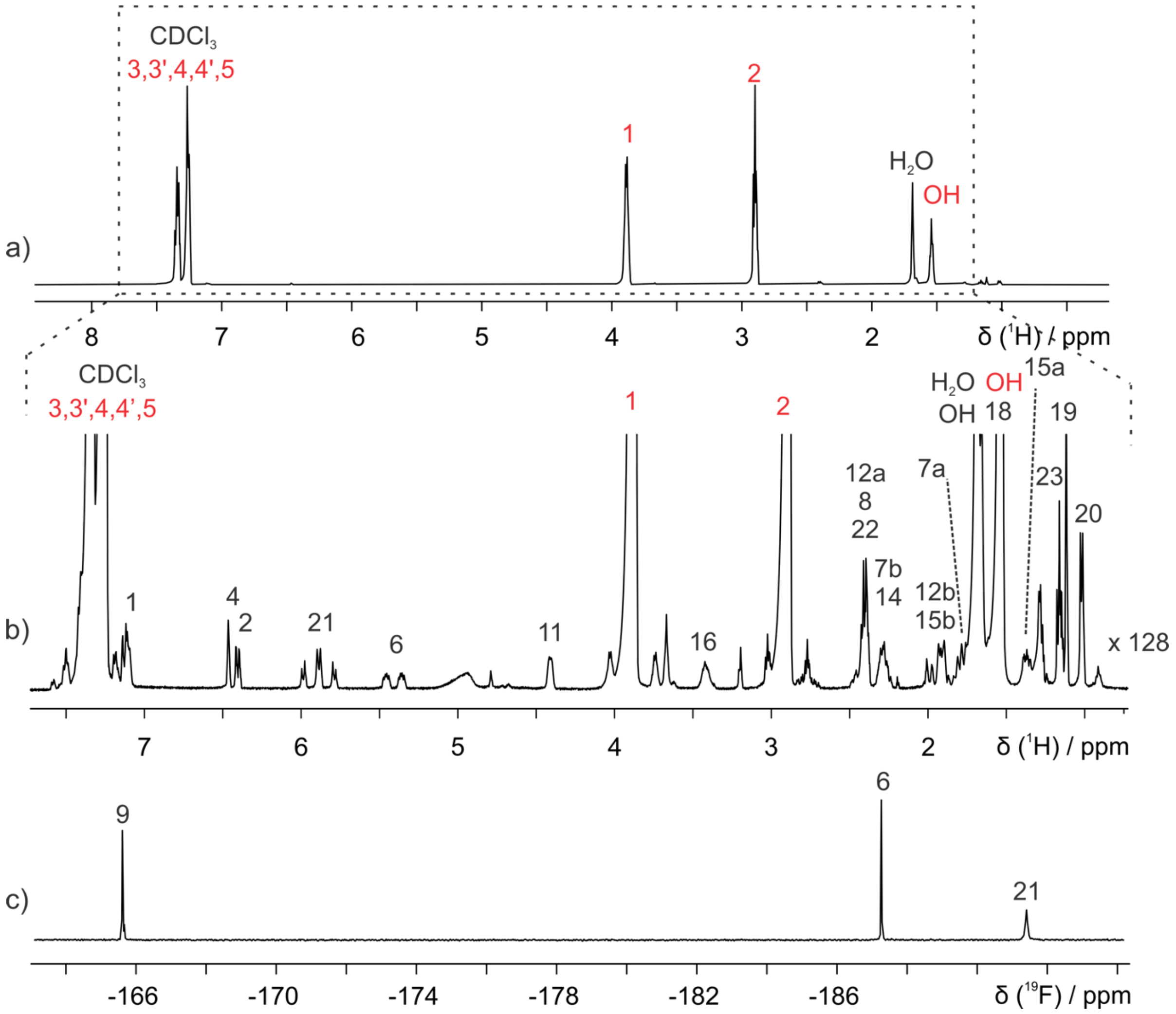

Figure S2: 500 and 470 MHz NMR ^{1}H and ^{19}F spectra of extracted fluticasone propionate in $CDCl_3$: (a) conventional ^{1}H, (b) vertically scaled conventional ^{1}H to show signals of interest, and (c) $^{19}F\{^{1}H\}$ NMR spectra. Peak assignments for fluticasone propionate are shown in black, and those for the major extraction excipient 2-phenylethanol in red. Other small impurity signals have not been assigned.

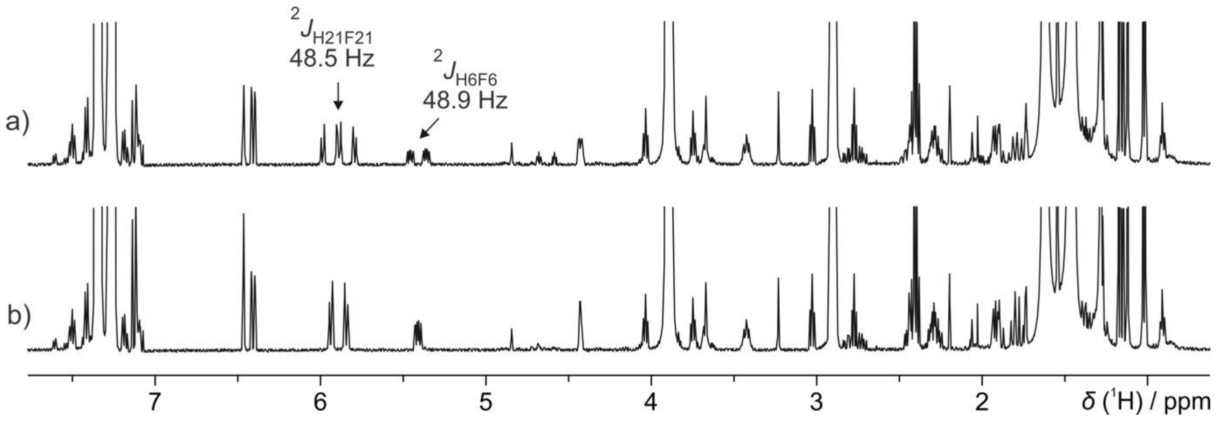

Figure S3: 500 MHz ^{1}H NMR spectra of extracted fluticasone propionate in $CDCl_3$: (a) conventional ^{1}H and (b) $^{1}H\{^{19}F\}$ using adiabatic decoupling sequence 'p5m4sp180.2' with WURST-80 pulses. Only J_{HF} couplings that can confidently be measured are marked, in spectrum (a).

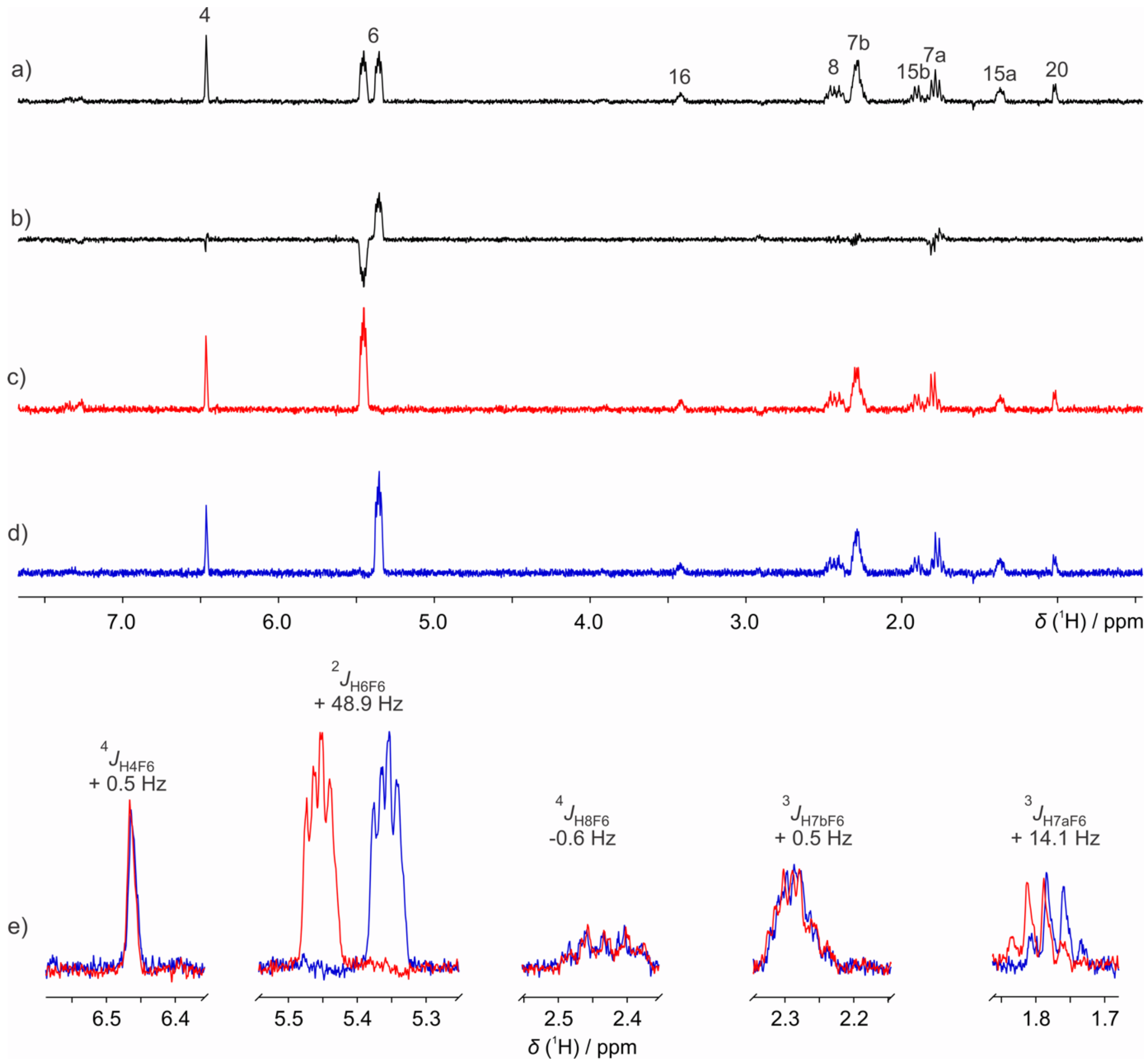

Figure S4: 500 MHz MODO-FESTA NMR spectra of extracted fluticasone propionate in $CDCl_3$: (a) IP-MODO-FESTA, (b) AP-MODO-FESTA, (c) (IP-AP)-MODO-FESTA and (d) (IP+AP)-MODO-FESTA. In the MODO element, F6 and H6 in fluticasone were selected at –187 ppm and 5.4 ppm, respectively. A scaling factor *k* of 1.072 was applied to the AP data when generating the (IP-AP) and (IP+AP) spectra.

2.2. Fluorohydrin

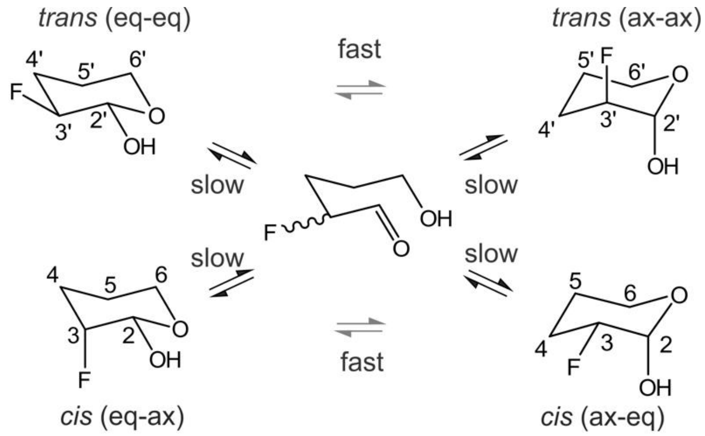

Scheme S2: Equilibrium between the *cis*- and *trans*- isomers of fluorohydrin, each with 2 main conformers. Fast (grey arrows) and slow (black arrows) relate to the conformer exchange rate on the chemical shift timescale.

The *cis*- and *trans*-3-fluoro-2-hydroxytetrahydropyran compounds were previously prepared[4] in our (CT) laboratory. According to the procedure described in the literature,[5] a solution containing nitromethane (50 mL), water (10 mL) and 3,4-dihydro-2*H*-pyran (1.83 mL, 0.02 mol) was cooled to 3 °C and selectfluor (10 g, 0.03 mol) was added. The reaction was stirred at 25 °C for 12 h. After that, the solution was refluxed (110 °C) for 1 h and concentrated under reduced pressure to remove the nitromethane. The crude residue was dissolved with dichloromethane (50 mL), followed by the addition of sodium bicarbonate (5%) solution. The organic layer was washed with brine, dried with magnesium sulfate, and concentrated under reduced pressure. The crude product was chromatographed over SiO_2 (70-230 mesh) using 7:3 hexane/ethyl acetate as eluent, and a mixture of *cis*- and *trans*-3-fluoro-2-hydroxytetrahydropyran diastereomers was isolated. The three fluorohydrin-based mixtures used in this work contained 0.68 M of 3-fluoro-2-hydroxytetrahydropyran in DMSO-d_6 (99.9 atom % D, Sigma-Aldrich), 0.58 M in D_2O (99.9 atom % D, Sigma-Aldrich), and 0.74 M in $CDCl_3$ (99.9 atom % D, Sigma-Aldrich). Fluorohydrin spectra were recorded at 298 K on a Bruker Avance III 500 MHz spectrometer (Bruker Biospin) with a 5 mm BBO probe with standard transmitter routing, equipped with a z-gradient coil with a maximum nominal gradient strength of 53.5 G cm^{-1}, operating at 500 MHz and 470 MHz for ^{1}H and ^{19}F, respectively. Data were processed using TopSpin software (version 4.0.9, Bruker BioSpin).

For ^{1}H NMR experiments, all acquisition parameters are given in Table S2. Prior to Fourier transformation of all 1D ^{1}H NMR data, zero-filling to 131072 complex points was applied. For ^{19}F experiments, the ^{19}F spectral window was set to 47619 Hz (101.26 ppm), the carrier frequency to –87014 Hz (–185 ppm), and the duration of the hard 90° pulse to 16.6 µs. 1D ^{19}F NMR data were acquired with 4 transients, a TD of 32768, and an experiment time of 25 s. {^{19}F}^{1}H NMR spectra were acquired using the CPD sequence WALTZ16 with a pulse duration of 80 µs and power level of 0.58 W. Prior to Fourier transformation of 1D ^{19}F NMR data, zero-filling to 131072 complex points was applied, and Gaussian weighting with LB of –0.01 Hz and GB of 0.005 was used.

Table S2: ^{1}H NMR experimental parameters: fluorohydrin in $CDCl_3$, DMSO-d_6, and D_2O.

Solvent	Spectral window		o1p		Hard 90° pulse duration / µs	Transients	Time domain data length	Experiment time
	/ Hz	/ ppm	/ Hz	/ ppm				
**$CDCl_3$**	5000	10.00	1999	4.0	12.15	16	32768	1 min 22 s
**DMSO-d_6**	5000	10.00	1999	4.0	12.20	32	32768	2 min 29 s
**D_2O**	5000	10.00	1999	4.0	12.15	32	32768	2 min 29 s

^{1}H IPAP-FESTA spectra were recorded with 32 transients, except for *trans*-fluorohydrin in $CDCl_3$, which was recorded with 16 transients. The chemical shift of the ^{1}H and ^{19}F signal selected in each experiment is shown in Table 3. ^{1}H signals ware selected using an rSNOB shaped pulse (4.65 ms, 450 Hz effective bandwidth) and ^{19}F signals using iBURP shaped pulses (32.5 ms, 1650 Hz effective bandwidth. These shaped pulses were designed using *Wavemaker*. A 150 ms mixing period was used for the DIPSI-2 (d9) element. When generating IPAP-FESTA by addition/subtracting processing, a scaling factor (*k*) was applied to the AP spectrum (Table S4). Prior to Fourier transformation of IPAP data, zero-filling to 131072 complex points was applied, and Gaussian weighting with LB of –0.01 Hz and GB of 0.013 was used.

Table S3*:* IPAP-FESTA experimental parameters for *cis*/trans fluorohydrin samples in $CDCl_3$, DMSO-d_6 and D_2O.

Solvent	Conformer	Sel. ^{19}F / ppm	Sel. ^{1}H / ppm	nF	J_{HF} / Hz	180° sel. ^{19}F pulse / ms	180° sel. ^{1}H pulse / ms	Mixing time / ms	Experiment time
**$CDCl_3$**	*cis*	-198.37	4.54	1	48.2	4.65	32.5	150	3 min 50 s
	trans	-189.69	4.35	1	48.2	4.65	32.5	150	2 min 33 s
**DMSO-d_6**	*cis*	-189.80	4.45	1	48.8	4.65	32.5	150	3 min 50 s
	trans	-186.42	4.23	1	48.8	4.65	32.5	150	3 min 50 s
**D_2O**	*cis*	-200.75	4.60	1	48.7	4.65	32.5	150	3 min 50 s
	trans	-187.68	4.30	1	48.4	4.65	32.5	150	3 min 50 s

Table S4: Scaling factors (*k*) applied to AP data when generating IPAP-FESTA spectra by addition/subtracting processing.

Solvent	Conformer	Scaling factor (*k*)
**$CDCl_3$**	*cis*	0.9308
	trans	1.096
**DMSO-d_6**	*cis*	0.9320
	trans	1.126
**D_2O**	*cis*	0.8893
	trans	1.125

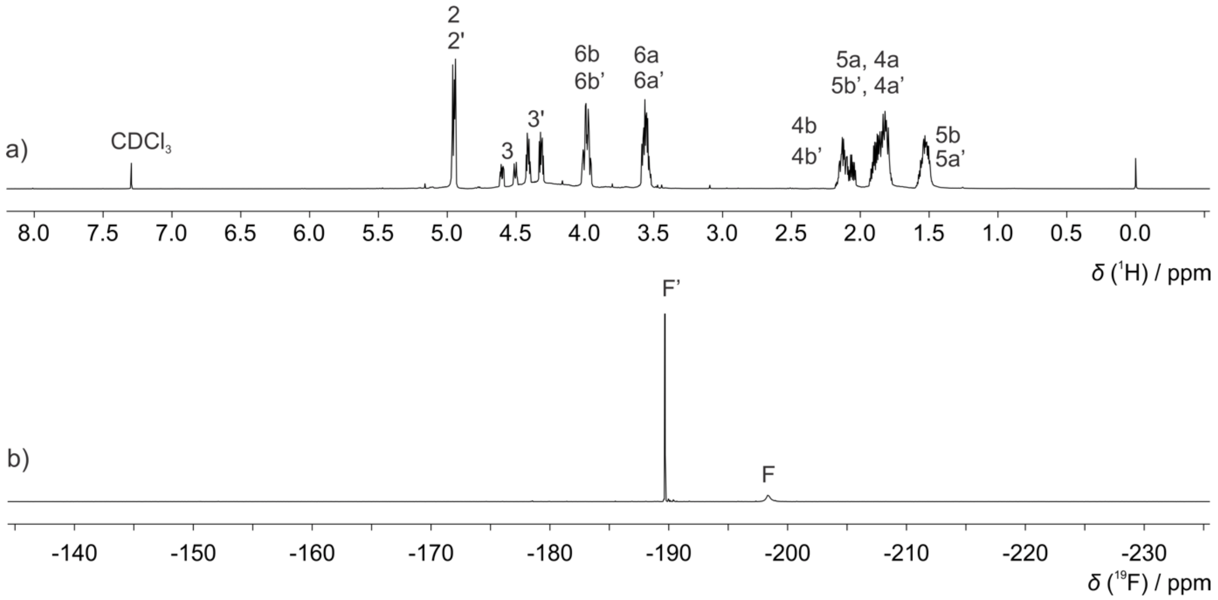

Figure S5: 500 and 470 MHz NMR spectra of 0.74 M fluorohydrin mixture in $CDCl_3$: (a) conventional ^1H and (b) $^{19}F\{^1H\}$ NMR. Peak assignments for *cis*-fluorohydrin and *trans*-fluorohydrin (the latter denoted by an apostrophe) are shown in (a) and (b). Small impurity peaks have not been assigned. In proton labels, 'a' corresponds to axial and 'b' to equatorial.

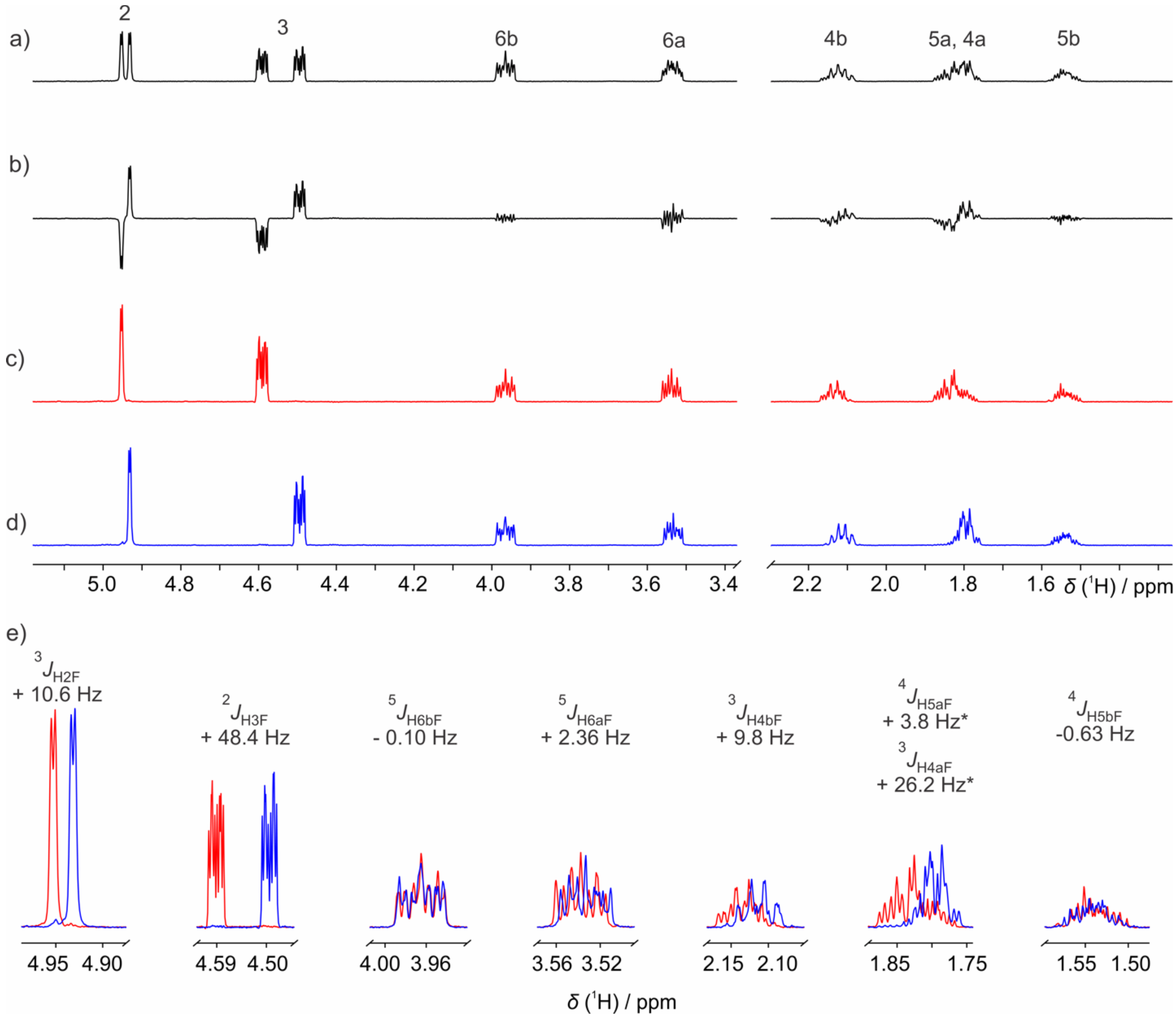

Figure S6: 500 MHz MODO-FESTA NMR spectra of *cis*-fluorohydrin in $CDCl_3$: (a) IP-MODO-FESTA, (b) AP-MODO-FESTA, (c) (IP-AP)-MODO-FESTA, and (d) (IP+AP)-MODO-FESTA. In the MODO element, F3 and H3 in *cis*-fluorohydrin were selected at –198.37 ppm and 4.54 ppm, respectively. A scaling factor of 0.931 was applied to the AP data when generating the (IP-AP) and (IP+AP) spectra. Peak assignments are shown in (a). Extracted J_{HF} values are shown in (e). Due to severe peak overlap, the J_{HF} values for $^4J_{5aF}$ and $^3J_{4aF}$ were measured using HD-HAPPY-FESTA (data not shown). In proton labels, 'a' corresponds to axial and 'b' to equatorial.

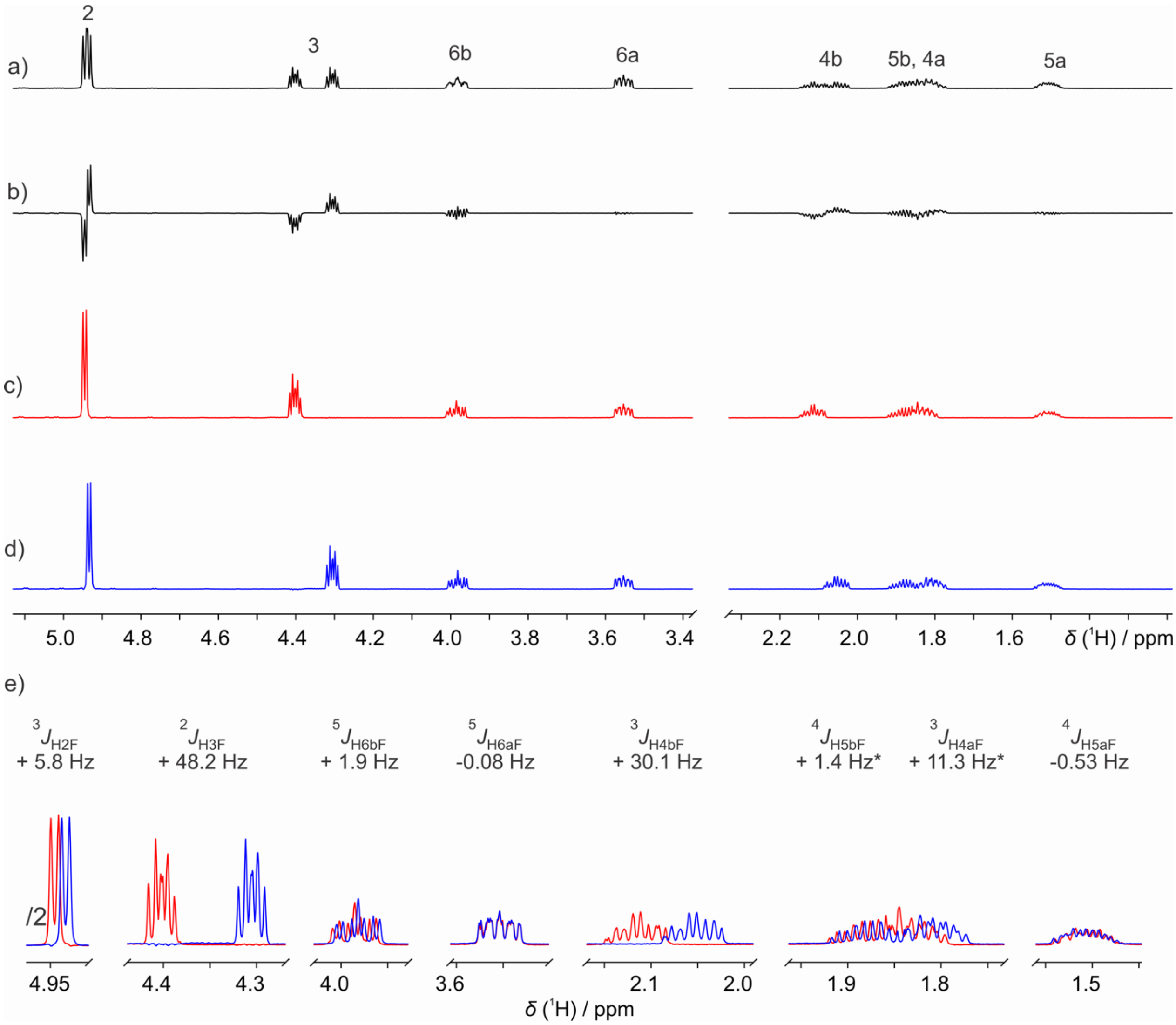

Figure S7: 500 MHz MODO-FESTA NMR spectra of *trans*-fluorohydrin in $CDCl_3$: (a) IP-MODO-FESTA, (b) AP-MODO-FESTA, (c) (IP-AP)-MODO-FESTA, and (d) (IP+AP)-MODO-FESTA. In the MODO element, F3 and H3 in *trans*-fluorohydrin were selected at –189.69 ppm and 4.35 ppm, respectively. A scaling factor of 1.096 was applied to the AP data when generating the (IP-AP) and (IP+AP) spectra. Peak assignments are shown in (a). Extracted J_{HF} values are shown in (e). The signal at 4.95 ppm in (e) was scaled by a factor of 0.5. The J_{HF} value of +11.3 Hz corresponding to $^{3}J_{4aF}$ was measured manually due to signal overlap. In proton labels, ‘a’ corresponds to axial and ‘b’ to equatorial.

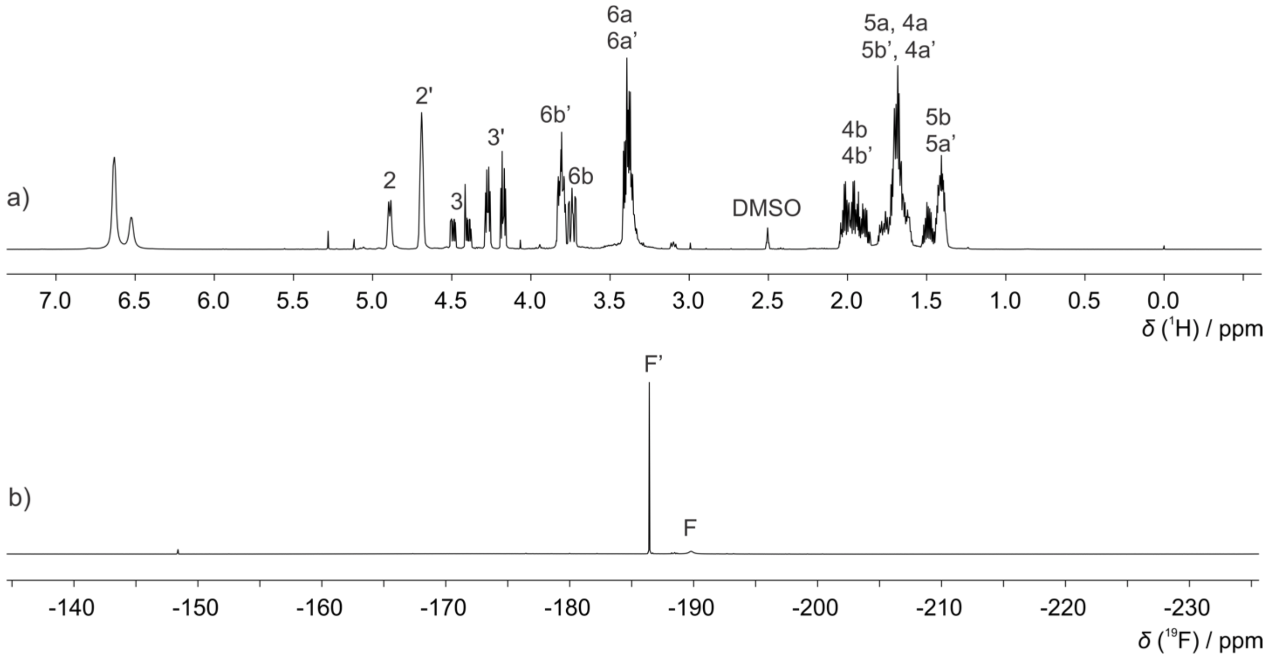

Figure S8: 500 MHz NMR spectra of 0.68 M fluorohydrin based mixture in DMSO-d_6: (a) conventional ^{1}H, (b) ^{19}F{^{1}H} NMR. Peak assignments for *cis*-fluorohydrin and *trans*-fluorohydrin (denoted by an apostrophe) are shown in (a) and (b). Small impurity peaks have not been assigned. In proton labels, 'a' corresponds to axial and 'b' to equatorial.

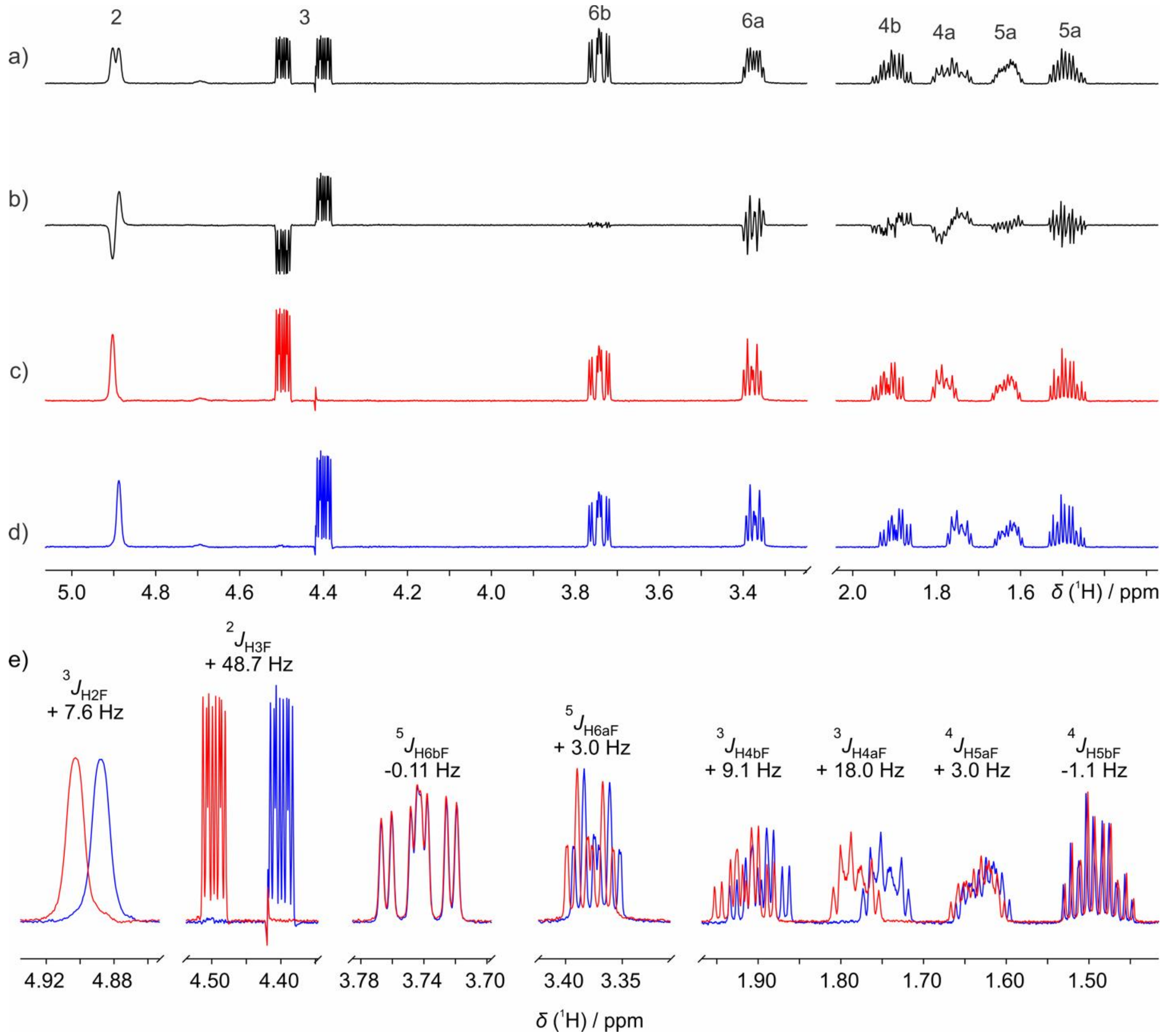

Figure S9: 500 MHz MODO-FESTA NMR spectra of *cis*-fluorohydrin in DMSO-d_6: (a) IP-MODO-FESTA, (b) AP-MODO-FESTA, (c) (IP-AP)-MODO-FESTA, (d) (IP+AP)-MODO-FESTA. In the MODO element, F3 and H3 in *cis*-fluorohydrin were selected at –189.80 ppm and 4.45 ppm, respectively. A scaling factor of 0.9320 was applied to the AP data when generating the (IP-AP) and (IP+AP) spectra. Peak assignments are shown in (a). Extracted J_{HF} values are shown in (e). In proton labels, ‘a’ corresponds to axial and ‘b’ to equatorial.

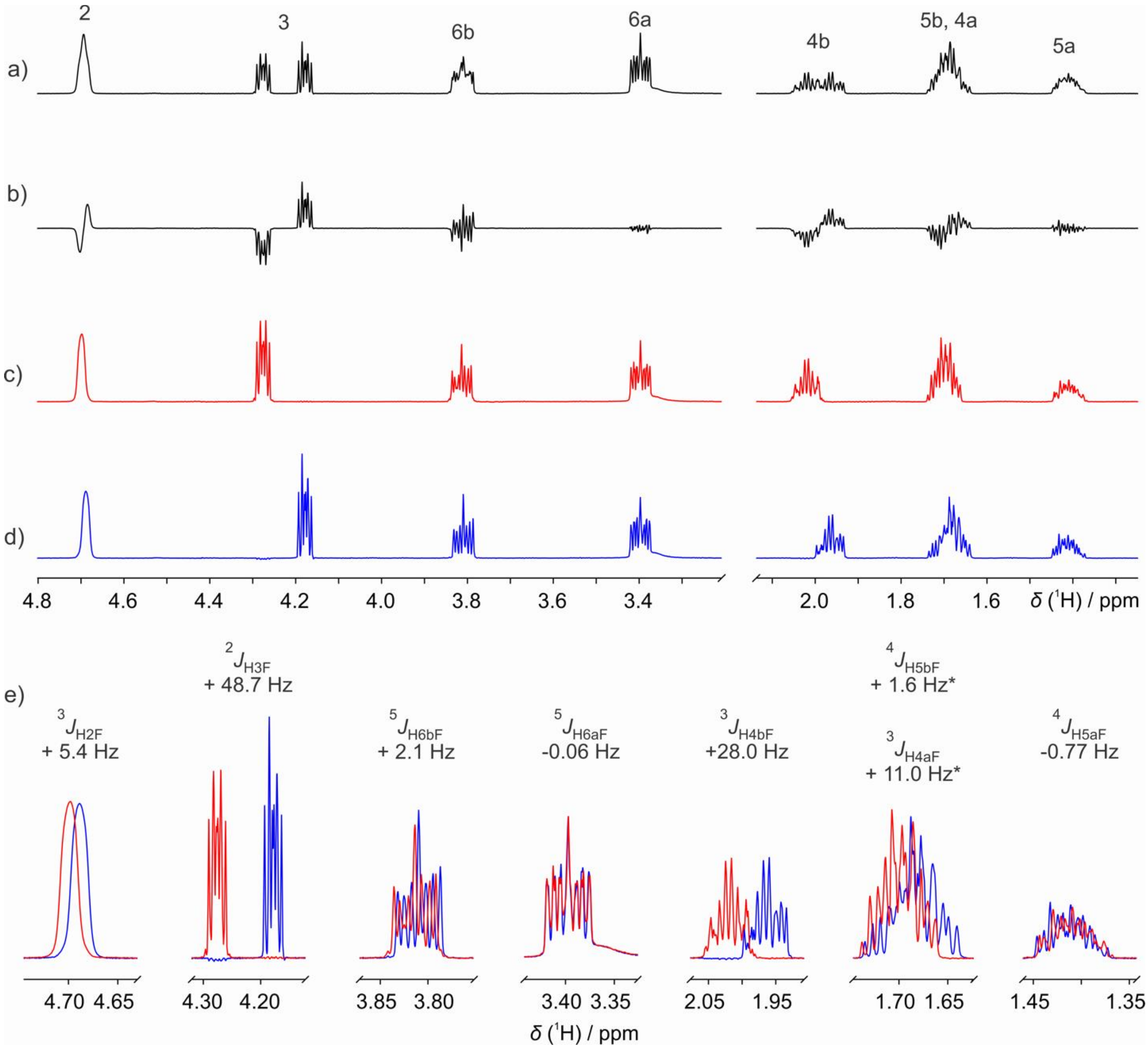

Figure S10: 500 MHz MODO-FESTA NMR spectra of *trans*-fluorohydrin in DMSO-d_6: (a) IP-MODO-FESTA, (b) AP-MODO-FESTA, (c) (IP-AP)-MODO-FESTA, (d) (IP+AP)-MODO-FESTA. In the MODO element, F3 and H3 in *trans*-fluorohydrin were selected at -186.42 ppm and 4.23 ppm, respectively. A scaling factor of 1.126 was applied to the AP data when generating the (IP-AP) and (IP+AP) spectra. Peak assignments are shown in (a). Extracted J_{HF} values are shown in (e). J_{HF} values for $^4J_{5bF}$ and $^3J_{4aF}$ were measured manually due to signal overlap. In proton labels, 'a' corresponds to axial and 'b' to equatorial.

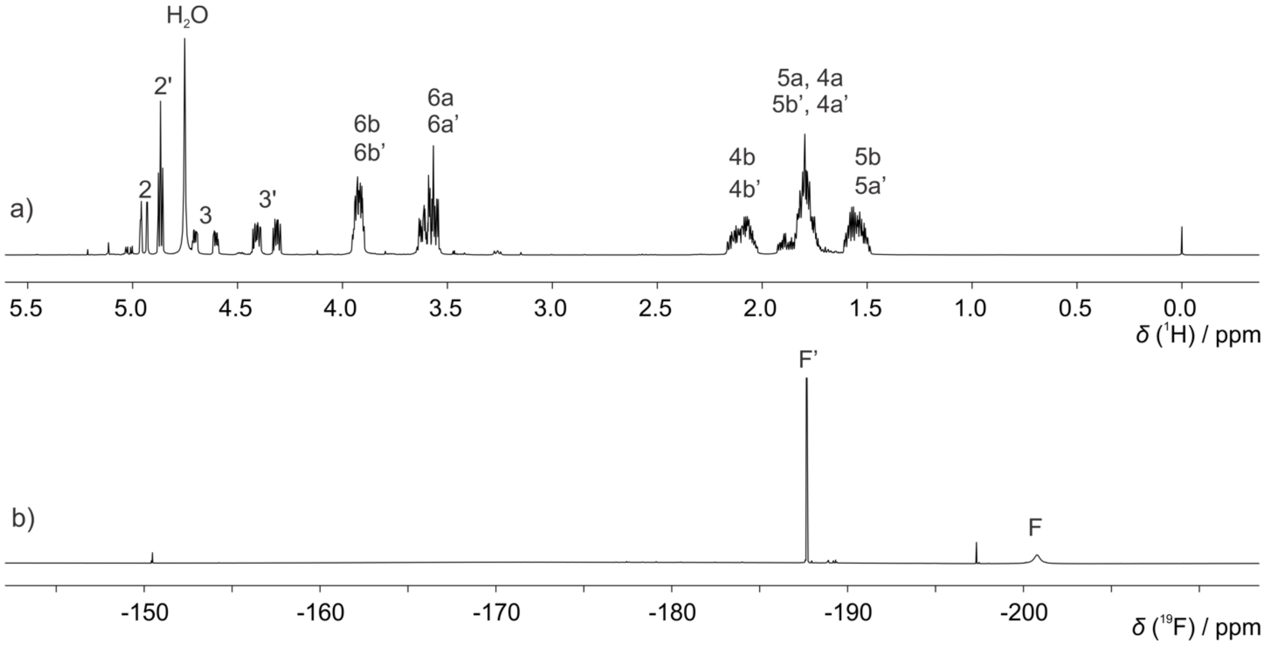

Figure S11: 500 MHz NMR spectra of 0.58 M fluorohydrin based mixture in D_2O: (a) conventional ^1H and (b) $^{19}F\{^1H\}$ NMR. Peak assignments for *cis*-fluorohydrin and *trans*-fluorohydrin (denoted by an apostrophe) are shown in (a) and (b). Small impurity peaks have not been assigned. In proton labels, 'a' corresponds to axial and 'b' to equatorial.

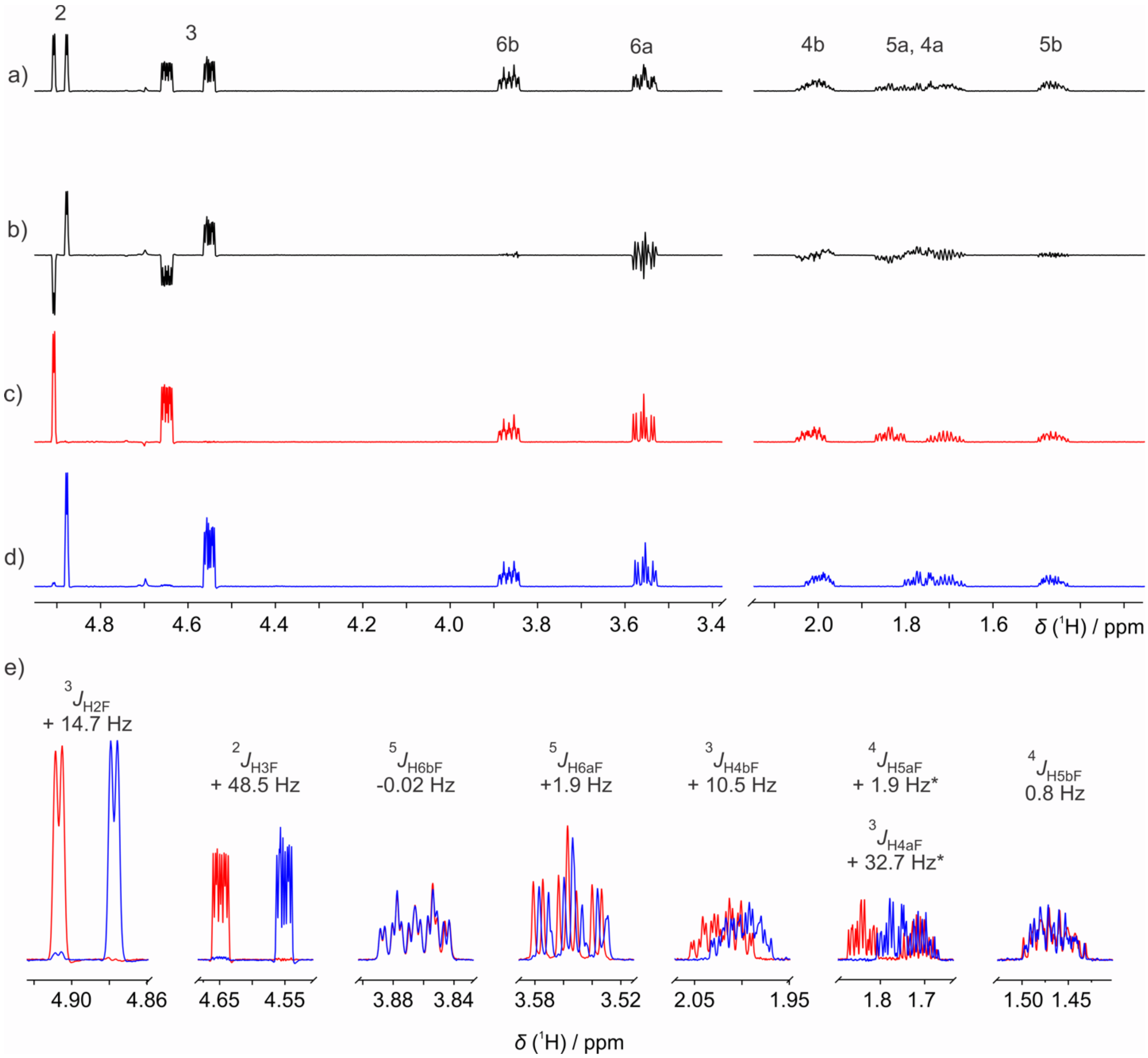

Figure S12: 500 MHz MODO-FESTA NMR spectra of *cis*-fluorohydrin in D_2O: (a) IP-MODO-FESTA, (b) AP-MODO-FESTA, (c) (IP-AP)-MODO-FESTA, (d) (IP+AP)-MODO-FESTA. In the MODO element, F3 and H3 in *cis*-fluorohydrin were selected at – 200.75 ppm and 4.60 ppm, respectively. A scaling factor of 0.8893 was applied to the AP data when generating the (IP-AP) and (IP+AP) spectra. Peak assignments are shown in (a). Extracted J_{HF} values are shown in (e). The J_{HF} value for $^4J_{5eqF}$ was measured using HD-HAPPY-FESTA due to severe peak overlap. J_{HF} values for $^4J_{5bF}$ and $^3J_{4aF}$ were measured manually due to signal overlap. In proton labels, 'a' corresponds to axial and 'b' to equatorial.

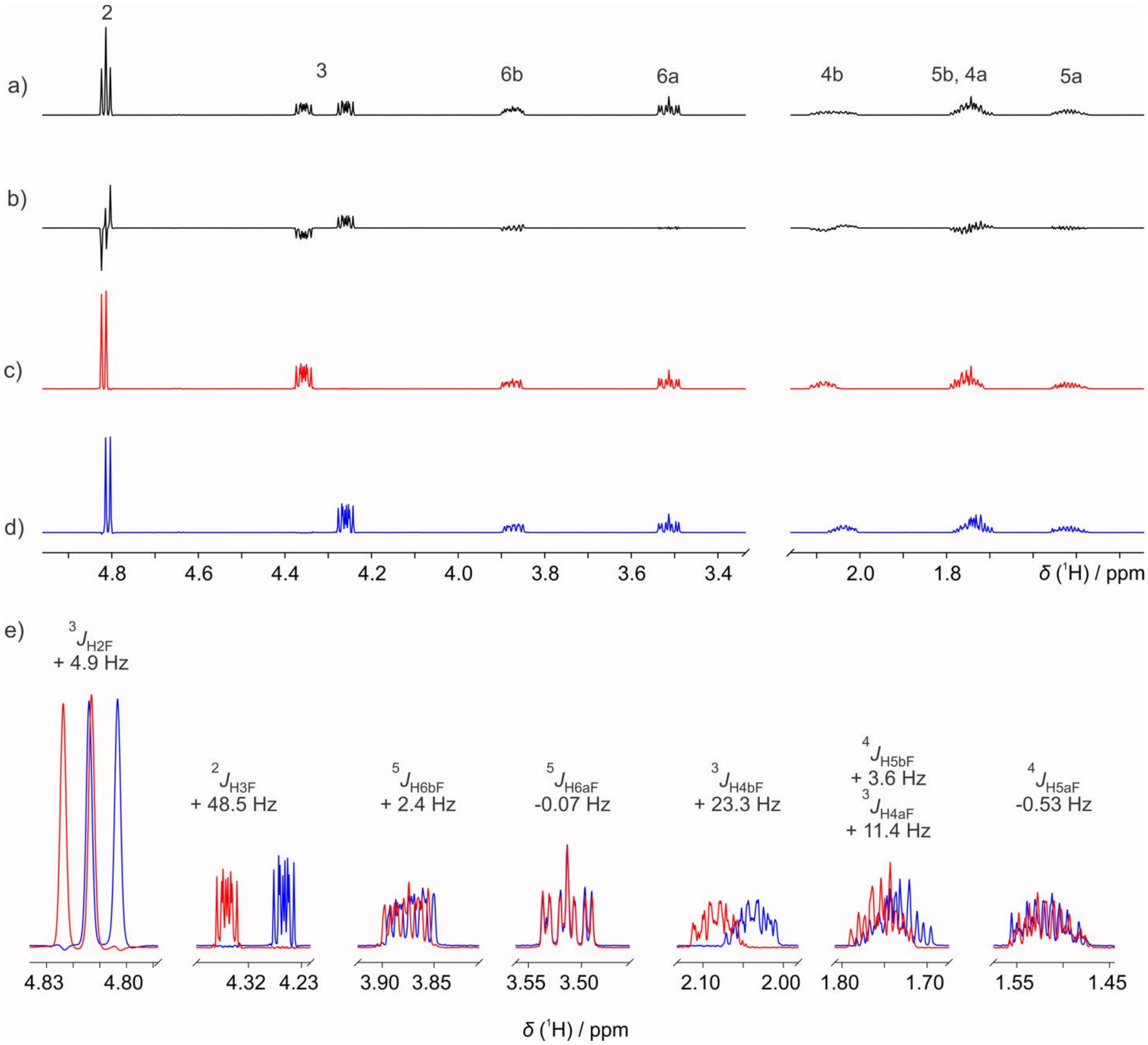

Figure S13: 500 MHz MODO-FESTA NMR spectra of *trans*-fluorohydrin in D_2O: (a) IP-MODO-FESTA, (b) AP-MODO-FESTA, and (c) (IP-AP)-MODO-FESTA and (d) (IP+AP)-MODO-FESTA. In the MODO element, F3 and H3 in *trans*-fluorohydrin were selected at -187.68 ppm and 4.30 ppm, respectively. A scaling factor of 1.125 was applied to the AP data when generating the (IP-AP) and (IP+AP) spectra. Peak assignments are shown in (a). Extracted J_{HF} values are shown in (e). The J_{HF} value for $^4J_{5bF}$ was measured from the HD-HAPPY-FESTA spectrum due to severe peak overlap. In proton labels, 'a' corresponds to axial and 'b' to equatorial.

2.3. Comparison of J_{HF} couplings measured using IPAP-FESTA and using ^{1}H and ^{1}H{^{19}F} NMR spectra

To confirm the accuracy of the J_{HF} values obtained from IPAP-FESTA NMR data, they were compared to the J_{HF} values of all the well-resolved signals in the ^{1}H NMR spectra of the fluorohydrin and fluticasone samples. Excellent agreement is seen between J_{HF} couplings measured using IPAP-FESTA and with ^{1}H and ^{1}H{^{19}F} NMR spectra, as shown in Table S5.

Table S5: Comparison of J_{HF} values measured from well-resolved signals in the ^{1}H NMR spectra to the corresponding J_{HF} values obtained from the IPAP-FESTA spectra for all samples studied.

Sample	Solvent	Isomer	Assignment	Measured J_{HF} / Hz		Difference / Hz
				^{1}H NMR	IPAP-FESTA	
Fluorohydrin	$CDCl_3$	*cis*	H3	48.5	48.4	0.1
		trans	H3	48.3	48.2	0.1
	DMSO-d_6	*cis*	H2	6.9*	7.6	-0.7
		cis	H3	48.7	48.7	0.0
		trans	H3	48.7	48.7	0.0
	D_2O	*cis*	H2	14.7	14.7	0.0
		trans	H2	5.0	4.9	0.1
		cis	H3	48.7	48.5	0.2
		trans	H3	48.5	48.5	0.0
Fluticasone	$CDCl_3$	N/A	H6	48.8	48.9	-0.1

* Broad doublet.

2.4. Cross-talk artifacts and the scaling factor *k* in IPAP data

Small systematic differences in peak intensity between the IP and AP data sets result in unwanted cross-talk artifacts when generating IPAP spectra by addition/subtraction processing. It is recommended to scale one spectrum, here the AP spectrum, by a scaling factor *k* to minimise such artifacts and obtain cleaner spectra. To highlight the importance of the scaling factor *k*, the IP and AP FESTA ^{1}H NMR spectra of *trans*-fluorohydrin in DMSO-d_6 were processed using the TopSpin AU macro '*split*', using a *k* factor of 1 (no scaling) and 1.126 (optimized scaling factor). Results are shown in Figure S14, where cross-talk artifacts for H3 in the IP-AP spectrum are reduced from a signal intensity of 7.5 % to 0.8 % when using the optimized scaling factor. Cross-talk artifacts for H4b are also highlighted, but signal intensities are not reported due to a small spectral overlap with the wanted signal.

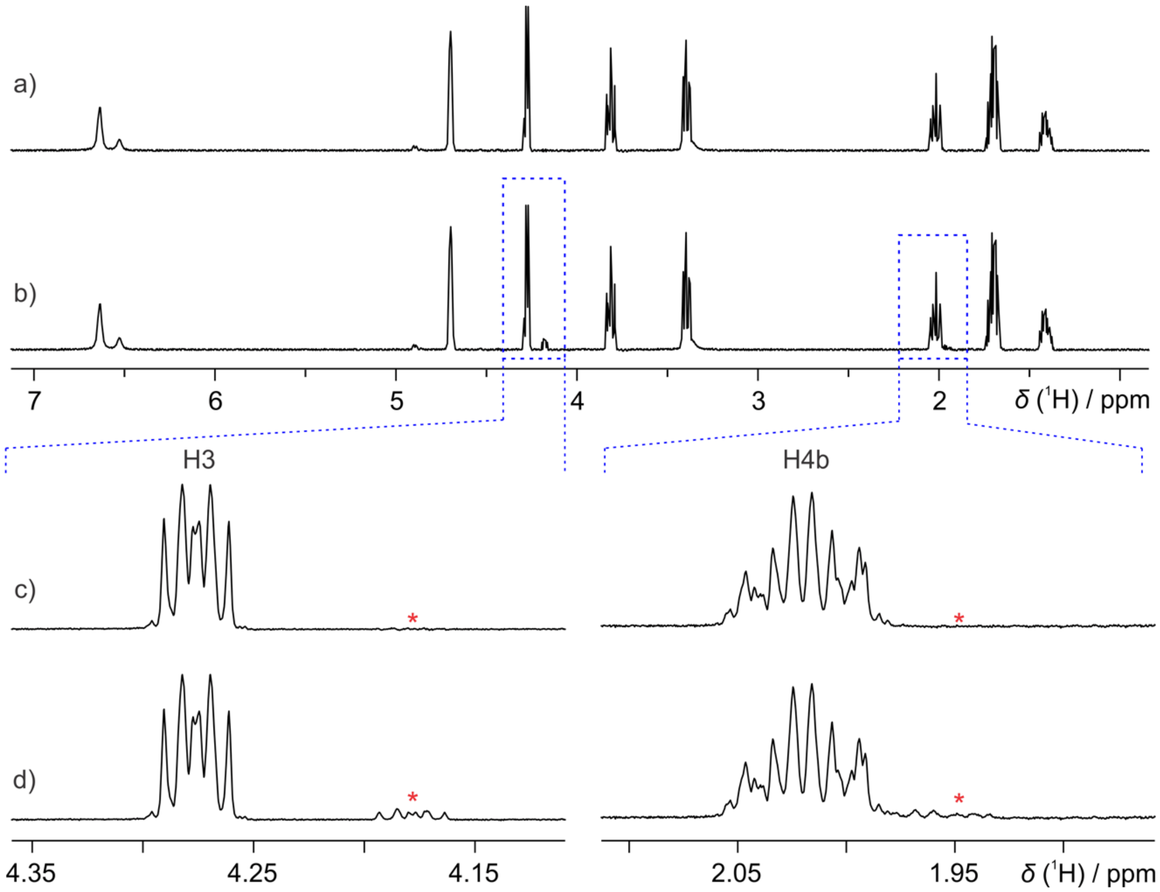

Figure S14: 500 MHz (IP-AP)-FESTA NMR spectra of *trans*-fluorohydrin in DMSO-d_6 where (a) optimized (k=1.126) and (b) unoptimized (k =1) scaling was applied to the AP-FESTA spectrum before addition/subtraction processing. Spectra (c) and (d) are expansions of (a) and (b), respectively. Cross-talk artifacts are highlighted by red asterisks in (c) and (d).

3. Computational data and measured IPAP-FESTA data

3.1. Fluticasone propionate J_{HF} values

All J_{HF6} couplings for fluticasone propionate obtained using IPAP-FESTA data are shown in Table S6. The Mathematica notebook used to measure J_{HF} values and the raw data are available for download at DOI: [10.48420/21975908](https://doi.org/10.48420/21975908).

Table S6: J_{HF} couplings for fluticasone propionate in $CDCl_3$ obtained using IPAP-FESTA with H6 at 5.41 ppm and F6 at –187.4 ppm selected. The positive sign of the $^2J_{H6F6}$ value was used as a reference for all other J-couplings as $^2J_{HF}$ couplings are typically large and positive. IPAP-FESTA spectra with J_{HF} couplings are shown in Figure S4.

**δ / ppm**	$^nJ_{H(X)F6}$	***J* / Hz**
6.46	$^4J_{H4F6}$	+ 0.5
5.41	$^2J_{H6F6}$	+ 48.8
2.43	$^4J_{H8F6}$	- 0.6
2.29	$^3J_{H7bF6}$	+ 0.5
1.79	$^3J_{H7aF6}$	+ 14.1

3.2. Potential energy curves for *cis*- and *trans*- fluorohydrin

The potential energy curves for the *cis*- (Figure S14, left) and *trans*- (Figure S14, right) fluorohydrin show the dihedral angle dependence for the ‘H-O-C_2-C_3’ bond. For each of the conformers the C_3-C_2-O-H dihedral angle was fixed and the rest of the molecule was allowed to relax during the geometry optimization calculations. Local minimum energies (most stable conformers) were used to describe each system. The potential energy curves for *cis*- and *trans*-fluorohydrin were scanned at the B3LYP/cc-pVDZ level of theory.

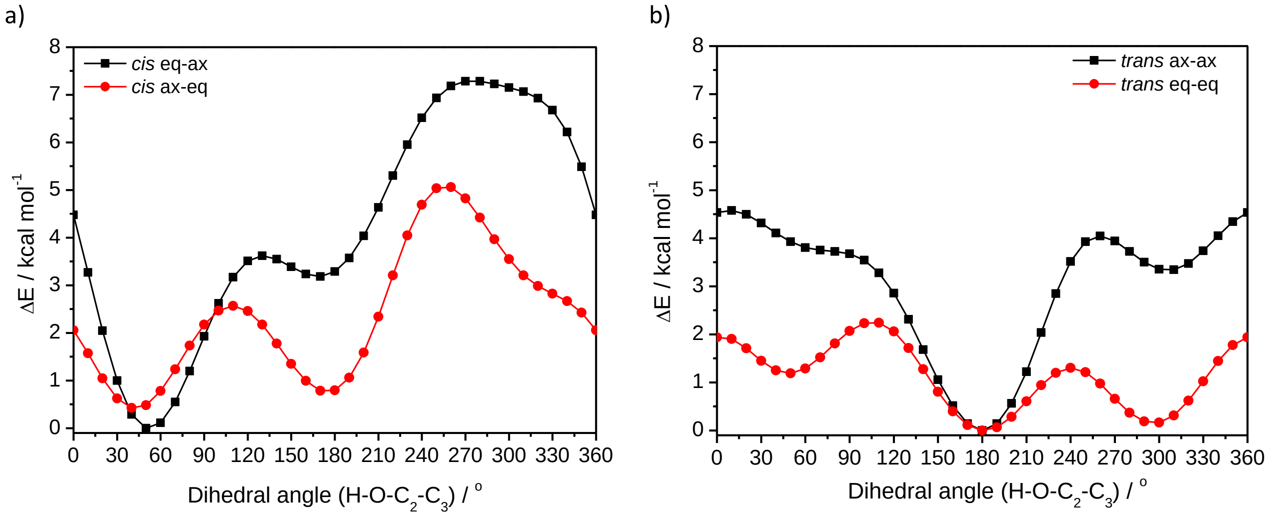

Figure S15: Potential energy curves calculated at the B3LYP/cc-pVDZ level of theory for a) *cis*- and b) *trans*-3-fluoro-2-hydroxytetrapyran.

Table S7: Gibbs energy differences for the major conformers, calculated at the M062X/aug-cc-pVTZ level, with respect to the eq-ax-50 and ax-ax-180 conformers for *cis* and *trans* respectively.

	cis						***trans***			
	**ΔG / kcal mol⁻¹**						**ΔG / kcal mol⁻¹**			
**Conformers**	**Isolated phase**	**$CHCl_3$**	**DMSO**	**H_2O**		**Conformers**	**Isolated phase**	**$CHCl_3$**	**DMSO**	**H_2O**
ax-eq-180	0.4	0.4	0.8	0.8		ax-ax-180	0.0	0.0	0.0	0.4
ax-eq-50	0.4	1.0	1.4	1.9		ax-ax-310	3.1	2.3	1.3	1.5
eq-ax-170	1.9	1.3	1.0	0.5		eq-eq-50	2.8	1.7	1.2	0.2
eq-ax-50	0.0	0.0	0.0	0.0		eq-eq-180	1.6	0.7	0.2	0.0
						eq-eq-300	1.8	0.8	0.1	0.1

Cartesian coordinates (xyz) for the four major conformers of the *cis* isomer in isolated phase.

***Cis*(ax-eq-180)**

Energy = -446.12546 a.u.
C -0.86186400 0.50198700 -0.45963900
C -0.39398200 -0.94599300 -0.38111200
C 1.81554200 -0.24496400 0.10917100
C 1.43091900 1.22479600 0.08230800
C -0.04345700 1.38554400 0.45480300
H -0.92139500 -1.55702400 -1.11597400
H 1.76626000 -0.62840600 1.13151000
H 2.82122300 -0.40272100 -0.27335800
H 2.06581900 1.78189400 0.77146100
H 1.60558200 1.62129500 -0.92054700
H -0.21522300 1.07126400 1.48531600
H -0.37236400 2.41915600 0.35382300
O 0.96370800 -1.02123000 -0.73205100
O -0.63349100 -1.40419600 0.91816500
H -0.48724300 -2.35333800 0.94326600
H -0.76081100 0.82587700 -1.49842500
F -2.20294800 0.56635400 -0.13324100

***Cis*(ax-eq-50)**

Energy = -446.125496 a.u.
C -0.82195100 0.52936200 -0.49593900
C -0.40522900 -0.93820100 -0.40650100
C 1.80074100 -0.32298800 0.17199400
C 1.49571200 1.16180900 0.07409100
C 0.02220900 1.40575400 0.40032800
H -0.93229500 -1.52592400 -1.16210200
H 1.68223300 -0.67266600 1.19997900
H 2.81171500 -0.54672800 -0.15968800
H 2.13595500 1.71773000 0.75898200
H 1.71720100 1.50638200 -0.93854800
H -0.17911100 1.13356300 1.43806300
H -0.25626000 2.45014300 0.26470000
O 0.94764200 -1.07626800 -0.68748700
O -0.66722900 -1.44485700 0.87953600
H -1.56126900 -1.18811600 1.12827500
H -0.76496800 0.85114200 -1.53742700
F -2.16060000 0.60322900 -0.11027400

***Cis*(eq-ax-170)**

Energy = -446.123081 a.u.
C -0.32730300 1.01229300 0.36641800
C -0.88503900 -0.39932400 0.46183700
C 1.17246100 -1.37834200 -0.09575600
C 1.85653000 -0.02798500 -0.23515600
C 1.16282600 1.00025600 0.65659500
H 1.29620500 -1.76196400 0.92641800
H 1.58197400 -2.11086900 -0.78725000
H 2.91001600 -0.11954400 0.02990300
H 1.79783200 0.29182400 -1.27579600
H 1.30846000 0.74538400 1.70975700
H 1.56464000 2.00078100 0.50242200
O -0.21053800 -1.28152100 -0.39844300
H -0.74277800 -0.74557100 1.50107300
O -2.22477900 -0.38443900 0.11575000
H -2.49073100 -1.28827200 -0.07868800
H -0.87792300 1.66176400 1.04899800
F -0.52689000 1.49030800 -0.91543600

***Cis*(eq-ax-50)**

Energy = -446.126095 a.u.
C -0.37198600 0.96388700 0.40273700
C -0.86103400 -0.47920900 0.48531800
C 1.26790900 -1.30461100 -0.09285300
C 1.86030400 0.08249800 -0.27615100
C 1.12247700 1.07093700 0.62213700
H 1.43208100 -1.65310100 0.93639400
H 1.71629900 -2.02724200 -0.77077900
H 2.92442900 0.06798300 -0.04014000
H 1.75145000 0.37860000 -1.31998200
H 1.32326700 0.84196800 1.67262400
H 1.44005400 2.09813600 0.44573600
O -0.12046400 -1.30792200 -0.37654500
H -0.75883800 -0.82955000 1.52074700
O -2.19787300 -0.58001200 0.15196600
H -2.30414400 -0.21201600 -0.73299800
H -0.93797400 1.57344100 1.10833100
F -0.68288300 1.42936000 -0.87449300

Cartesian coordinates (xyz) for the five major conformers of the *trans* isomer in isolated phase.

***Trans*(ax-ax-180)**

Energy = -446.127286 a.u.
C -0.42512500 -1.06069900 0.25727900
C 0.92930700 -0.61184600 -0.27888500
C 0.26145400 1.66645400 -0.26400000
C -1.15119400 1.31676000 0.16751700
C -1.12396100 0.04771000 1.01540300
H 1.29605200 -1.34294500 -1.00198500
H -0.28639000 -1.95121300 0.87002700
H 0.87436600 1.91069000 0.60897900
H 0.27639400 2.51386500 -0.94527900
H -1.57846200 2.14730700 0.72965000
H -1.76820300 1.15684400 -0.71746900
H -0.56920900 0.22297800 1.93936000
H -2.12738900 -0.28255300 1.28214600
O 0.86157900 0.59170500 -0.98380600
O 1.77445000 -0.52449200 0.84051900
H 2.64905900 -0.26474500 0.53760500
F -1.19970500 -1.42202200 -0.83784700

***Trans*(ax-ax-310)**

Energy = -446.122331 a.u.
C -0.23803500 -1.11214600 0.25475200
C 1.03009300 -0.45398300 -0.28733100
C -0.00796800 1.67975100 -0.28571900
C -1.34082800 1.11910800 0.18000500
C -1.10398800 -0.13141400 1.02251400
H 1.51153800 -1.12214300 -0.99785500
H 0.04056400 -1.97544500 0.85959100
H 0.57594800 2.04691700 0.56849300
H -0.14171100 2.51431600 -0.96986400
H -1.88298200 1.87395500 0.74966300
H -1.94066000 0.86034500 -0.69285900
H -0.60239000 0.13054300 1.95975600
H -2.03826300 -0.62184300 1.29380100
O 0.73905900 0.71031200 -1.00416700
O 1.95730900 -0.24948600 0.74617400
H 1.55160800 0.23718900 1.46860500
F -0.96447000 -1.58203800 -0.83119000

***Trans*(eq-eq-180)**

Energy = -446.124784 a.u.
C -0.70517800 1.42457200 0.08653100
C 0.59291300 0.74614900 -0.30333000
C 0.65621900 -0.66268600 0.26117100
C -1.69264900 -0.84732900 0.29310300
C -1.89396900 0.54268500 -0.28633400
H 0.71469000 0.68216000 -1.38709700
H -0.68571800 1.58541100 1.16811200
H -0.76080100 2.40334200 -0.38800500
H -2.47210900 -1.53605300 -0.02467200
H -1.69673000 -0.80330600 1.39035800
H -1.97827900 0.46122600 -1.37168900
H -2.82314600 0.97366400 0.08694200
H 0.66320100 -0.60897800 1.36240300
O -0.46872400 -1.40184000 -0.15821100
O 1.79233900 -1.28182400 -0.22600500
H 1.81528400 -2.18506300 0.10300900
F 1.65452000 1.47517400 0.20305900

***Trans*(eq-eq-300)**

Energy = -446.124449 a.u.
C -0.70762100 1.42397900 0.09529900
C 0.58537000 0.73776400 -0.29690000
C 0.65824600 -0.66863900 0.28782200
C -1.69511500 -0.84973400 0.28073000
C -1.89503500 0.54517700 -0.28799400
H 0.68341600 0.66661500 -1.38548800
H -0.68912700 1.57705800 1.17798000
H -0.75917500 2.40614000 -0.37300400
H -2.46833600 -1.53758700 -0.05380300
H -1.71441900 -0.81659900 1.37765000
H -1.97622500 0.47475900 -1.37460400
H -2.82614400 0.97166700 0.08550100
H 0.67170200 -0.60407900 1.38309000
O -0.46249300 -1.40139800 -0.15185100
O 1.80233200 -1.35059900 -0.08009700
H 1.77100100 -1.50775400 -1.02966700
F 1.65705900 1.47271900 0.17490900

***Trans*(eq-eq-50)**

Energy = -446.122791 a.u.
C -0.68160300 1.44009200 0.11895200
C 0.57827900 0.72187900 -0.30832800
C 0.61466200 -0.68649200 0.26597900
C -1.71394600 -0.82891900 0.26948900
C -1.88900900 0.58482400 -0.26664700
H 0.66438800 0.65348900 -1.39496400
H -0.64708300 1.57442300 1.20348700
H -0.72331400 2.42912700 -0.33543500
H -2.50347000 -1.48969100 -0.08008600
H -1.73070400 -0.82011200 1.36703000
H -1.98226100 0.53934800 -1.35329300
H -2.80793500 1.01986600 0.12621500
H 0.59703900 -0.61959700 1.36765600
O -0.49785100 -1.40029400 -0.18182300
O 1.72818100 -1.39760900 -0.16895200
H 2.50713000 -0.85299500 -0.01822500
F 1.70369600 1.39567500 0.16079300

3.3. Fluorohydrin conformer populations determination from Gibbs free energy

The Excel document used to calculate the conformer ratios for Table S8-S16 is available for download at DOI: 10.48420/21975908. The Boltzmann factor is the ratio of the population of each conformer to the population of the lowest energy conformer. The Gibbs energy (Tables S8-S15) was used to weight the populations of the conformations because it includes the entropic contribution to the Boltzmann distribution. Contributions from translational, electronic, rotational, and vibrational motion were considered.. For a single molecule, one of the most essential approximations throughout this analysis is that all the equations assume non-interacting particles and apply only to an ideal gas. To compute all contributions, the partition function for the corresponding component of the total partition function is used. Since the vibrational partition function depends on the frequencies, you must use a structure that is either a minimum or a saddle point. Therefore, the thermal energy (E^{therm}), enthalpy ($H=E^{therm}+PV$), entropy (S) and Gibbs free energy (G=H-TS) were computed at the default conditions of 298.15 K and 1 atmosphere, which can be obtained from frequency calculation.[6]

Table S8: Determination of conformer ratios for *cis*-fluorohydrin, isolated phase.

***Cis* isomer conformer**	**Gibbs free energy / a.u. [a]**	**ΔG / kcal mol^{-1}**	**Boltzmann factor**	**% of each conformer**	**Total % for (ax-eq) vs (eq-ax)**
Cis(ax-eq-180)	-446.125460	0.4	0.5107	24.52	50.0
Cis(ax-eq-50)	-446.125496	0.4	0.5305	25.48	
Cis(eq-ax-170)	-446.123081	1.9	0.0412	1.98	50.0
Cis(eq-ax-50)	-446.126095	0.0	1.0000	48.02	

[a] atom units

Table S9: Determination of conformer ratios for *trans*-fluorohydrin, isolated phase.

***Trans* isomer conformer**	**Gibbs free energy / a.u. [a]**	**ΔG / kcal mol^{-1}**	**Boltzmann factor**	**% of each conformer**	**Total % for (ax-ax) vs (eq-eq)**
Trans(ax-ax-180)	-446.127286	0.0	1.0000	88.15	88.6
Trans(ax-ax-310)	-446.122331	3.1	0.0053	0.47	
Trans(eq-eq-180)	-446.124784	1.6	0.0708	6.24	11.4
Trans(eq-eq-300)	-446.124449	1.8	0.0497	4.38	
Trans(eq-eq-50)	-446.122791	2.8	0.0086	0.76	

[a] atom units

Table S10: Determination of conformer ratios for *cis*-fluorohydrin in $CDCl_3$.

***Cis* isomer conformer**	**Gibbs free energy / a.u. [a]**	**ΔG / kcal mol^{-1}**	**Boltzmann factor**	**% of each conformer**	**Total % for (ax-eq) vs (eq-ax)**
Cis(ax-eq-180)	-446.138280	0.4	0.5322	29.38	39.0
Cis(ax-eq-50)	-446.137229	1.0	0.1750	9.66	
Cis(eq-ax-170)	-446.136742	1.3	0.1045	5.77	61.0
Cis(eq-ax-50)	-446.138876	0.0	1.0000	55.19	

[a] atom units

Table S11: Determination of conformer ratios for *trans*-fluorohydrin in $CDCl_3$.

***Trans* isomer conformer**	**Gibbs free energy / a.u. [a]**	**ΔG / kcal mol^{-1}**	**Boltzmann factor**	**% of each conformer**	**Total % for (ax-ax) vs (eq-eq)**
Trans(ax-ax-180)	-446.139431	0.0	1.0000	60.02	61.3
Trans(ax-ax-310)	-446.135813	2.3	0.0217	1.30	
Trans(eq-eq-180)	-446.138346	0.7	0.3172	19.04	38.7
Trans(eq-eq-300)	-446.138198	0.8	0.2712	16.28	
Trans(eq-eq-50)	-446.136706	1.7	0.0559	3.36	

[a] atom units

Table S12: Determination of conformer ratios for *cis*-fluorohydrin in DMSO-d_6.

Cis isomer conformer	Gibbs free energy / a.u. [a]	ΔG / kcal mol^{-1}	Boltzmann factor	% of each conformer	Total % for (ax-eq) vs (eq-ax)
Cis(ax-eq-180)	-446.138495	0.8	0.2603	16.88	23.0
Cis(ax-eq-50)	-446.137534	1.4	0.0941	6.11	
Cis(eq-ax-170)	-446.138185	1.0	0.1875	12.16	77.0
Cis(eq-ax-50)	-446.139767	0.0	1.0000	64.86	

[a] atom units

Table S13: Determination of conformer ratios for *trans*-fluorohydrin in DMSO-d_6.

Trans isomer conformer	Gibbs free energy / a.u. [a]	ΔG / kcal mol^{-1}	Boltzmann factor	% of each conformer	Total % for (ax-ax) vs (eq-eq)
Trans(ax-ax-180)	-446.139485	0.0	1.0000	35.92	39.6
Trans(ax-ax-310)	-446.137335	1.3	0.1028	3.69	
Trans(eq-eq-180)	-446.139243	0.2	0.7741	27.80	60.4
Trans(eq-eq-300)	-446.139251	0.1	0.7807	28.04	
Trans(eq-eq-50)	-446.137531	1.2	0.1265	4.54	

[a] atom units

Table S14: Determination of conformer ratios for *cis*-fluorohydrin in D_2O.

Cis isomer conformer	Gibbs free energy / a.u. [a]	ΔG / kcal mol^{-1}	Boltzmann factor	% of each conformer	Total % for (ax-eq) vs (eq-ax)
Cis(ax-eq-180)	-446.138697	0.8	0.2809	16.25	18.7
Cis(ax-eq-50)	-446.136915	1.9	0.0426	2.47	
Cis(eq-ax-170)	-446.139042	0.5	0.4046	23.41	81.3
Cis(eq-ax-50)	-446.139897	0.0	1.0000	57.87	

[a] atom units

Table S15: Determination of conformer ratios for *trans*-fluorohydrin in D_2O.

Trans isomer conformer	Gibbs free energy / a.u. [a]	ΔG / kcal mol^{-1}	Boltzmann factor	% of each conformer	Total % for (ax-eq) vs (eq-ax)
Trans(ax-ax-180)	-446.139098	0.4	0.5233	16.05	18.4
Trans(ax-ax-310)	-446.137264	1.5	0.0751	2.30	
Trans(eq-eq-180)	-446.139710	0.0	1.0000	30.67	81.6
Trans(eq-eq-300)	-446.139598	0.1	0.8882	27.24	
Trans(eq-eq-50)	-446.139468	0.2	0.7741	23.74	

[a] atom units

Table S16*:* Summary of the isomer and conformer populations for the *cis*- and *trans*-fluorohydrin isomers in DMSO-d_6, $CDCl_3$ and D_2O. The *cis*/*trans* ratio for each solvent were measured using the ^{1}H NMR integrals and conformer ratios were estimated using theoretical J_{HF} coupling values from DFT calculations.

	Relative percentages					
	^{1}H NMR (measured)		DFT (calculated)			
			cis conformers		*trans* conformers	
**Solvent**	*cis* isomer	*trans* isomer	ax-eq	eq-ax	ax-ax	eq-eq
**Chloroform**	33%	67%	39%	61%	61%	39%
**DMSO**	34%	66%	23%	77%	40%	60%
**Water**	40%	60%	19%	81%	18%	82%

3.4 J_{HF} values from DFT calculations and values extracted from experimental IPAP-FESTA data

The computational uncertainty in both the populations and *J*-values calculated via DFT is not discussed here as it is outside of the scope of this work. To determine the computational uncertainty, calculations using different methods (functional and basis set) and the evaluation of different solvent effect models (implicit and explicit) must be performed, which suggest using molecular dynamics approaches. Some coupling constants are population dependent, such as the $^3J_{HF}$ couplings, and deviation/variation between experimental and theoretical is more pronounced. This is not the case for $^2J_{HF}$ since in both conformations coupled nuclei are in a similar orientation, i.e., bond angle and bond distance are similar.

Table S17: J_{HF} values obtained from DFT calculations - *cis*-fluorohydrin, in isolated phase. From DFT calculations, the ratio of ax-eq conformer: eq-ax conformer was 1:1.

		cis **isomer and its conformations**		
		$^nJ_{HF}$ **in isolated phase**		
$^nJ_{H_XF}$		**Calculated with DFT / Hz**		
**n**	**X**	**(*cis* ax-eq)**	**(*cis* eq-ax)**	**(weighted average)**
2	3	54	54.8	54.4
3	2	0.8	15.4	8.1
3	4a	6.4	44.1	25.3
3	4b	7.9	9.8	8.9
4	5a	3.4	0.8	2.1
4	5b	-2	0.3	-0.9
5	6a	3.8	1.2	2.5
5	6b	-0.4	-0.2	-0.3

Table S18: J_{HF} values from DFT calculations - *trans*-fluorohydrin, in isolated phase. From DFT calculations, the ratio of ax-ax conformer: eq-eq conformer was 88.6:11.4.

		trans **isomer and its conformations**		
		$^nJ_{HF}$ **in isolated phase**		
$^nJ_{H_XF}$		**Calculated with DFT / Hz**		
**n**	**X**	***trans*(ax-ax)**	***trans*(eq-eq)**	**(weighted average)**
2	3	53.3	56.1	53.6
3	2	9.2	3.3	8.5
3	4a	10.6	12.1	10.8
3	4b	43.8	4.7	39.3
4	5a	0	-1.7	-0.2
4	5b	0.8	2.2	1.0
5	6a	-0.3	-0.3	-0.3
5	6b	1.4	3.1	1.6

Table S19: J_{HF} values obtained from DFT calculations and extracted from experimental IPAP-FESTA data (Figure S6) for *cis*-fluorohydrin in $CDCl_3$. From DFT calculations, the ratio of ax-eq conformer: eq-ax conformer was 39:61.

		***Cis* isomer and its conformations**				
		**$^nJ_{HF}$ in chloroform**				
**$^nJ_{H_XF}$**		**Calculated with DFT / Hz**			**Measured with IPAP-FESTA / Hz**	**^{1}H NMR (δ / ppm)**
**n**	**X**	**(*cis* ax-eq)**	**(*cis* eq-ax)**	**(weighted average)**		
2	3	53.9	55.5	54.9	48.4	4.54
3	2	0.7	17.3	10.8	10.6	4.94
3	4a	6.8	49.8	33.0	26.2§	1.82
3	4b	9.4	11.2	10.5	9.8	2.13
4	5a	4.1	0.7	2.0	3.8§	1.82
4	5b	-2.1	0.2	-0.7	-0.63	1.54
5	6a	4.3	1.2	2.4	2.4	3.54
5	6b	-0.4	-0.1	-0.2	-0.10	3.96

§ J_{HF} values measured using HD-HAPPY-FESTA data due to severe peak overlap.

Table S20: J_{HF} values calculated from DFT calculations and extracted from experimental IPAP analysis (Figure S7) for *trans*-fluorohydrin, in $CDCl_3$. From DFT calculations, the ratio of ax-ax conformer: eq-eq conformer was 61.3:38.7.

		***trans* isomer and its conformations**				
		**$^nJ_{HF}$ in chloroform**				
**$^nJ_{H_XF}$**		**Calculated with DFT / Hz**			**Measured with IPAP-FESTA / Hz**	**^{1}H NMR (δ / ppm)**
**n**	**X**	***trans*(ax-ax)**	***trans*(eq-eq)**	**(weighted average)**		
2	3	53.1	57.1	54.6	48.2	4.35
3	2	10.5	2.9	7.6	5.8	4.94
3	4a	7.5	13.3	9.7	11.3§	1.82
3	4b	50	5.5	32.8	30.1	2.09
4	5a	-0.2	-1.9	-0.9	-0.53	1.51
4	5b	0.7	2.7	1.5	1.4§	1.88
5	6a	-0.3	-0.3	-0.3	-0.79	3.55
5	6b	1.4	3.5	2.2	1.9	3.98

§ Overlaying signals. J_{HF} coupling extracted manually from IPAP data using TopSpin software.

Table S21: J_{HF} values from DFT calculations and extracted from experimental IPAP data (Figure S9) for *cis*-fluorohydrin, in DMSO-d_6. From DFT calculations, the ratio of ax-eq conformer: eq-ax conformer was 23:77.

		***cis* isomer and its conformations**				
		**$^nJ_{HF}$ in DMSO**				
**$^nJ_{H_XF}$**		**Calculated with DFT / Hz**			**Measured with IPAP-FESTA / Hz**	**^{1}H NMR (δ / ppm)**
**n**	**X**	**(*cis* ax-eq)**	**(*cis* eq-ax)**	**(weighted average)**		
2	3	54.3	56.1	55.7	48.7	4.45
3	2	0.8	17.9	14.0	7.6	4.9
3	4a	7.1	52.1	41.8	18.0	1.76
3	4b	9.7	11.8	11.3	9.1	1.91
4	5a	4.3	0.7	1.5	3.0	1.63
4	5b	-2.2	0.2	-0.4	-1.1	1.49
5	6a	4.5	1.2	2.0	3.0	3.38
5	6b	-0.4	-0.1	-0.2	-0.11	3.74

Table S22: J_{HF} values from DFT calculations and extracted from experimental IPAP data (Figure S10) for *trans*-fluorohydrin, in DMSO-d_6. From DFT calculations, the ratio of ax-ax conformer: eq-eq conformer was 39.6:60.4.

		***trans* isomer and its conformations**				
		**$^nJ_{HF}$ in DMSO**				
**$^nJ_{H_XF}$**		**Calculated with DFT / Hz**			**Measured with IPAP-FESTA / Hz**	**^{1}H NMR (δ / ppm)**
**n**	**X**	***trans*(ax-ax)**	***trans*(eq-eq)**	**(weighted average)**		
2	3	53.3	57.7	56.0	48.7	4.23
3	2	10.8	3.1	6.1	5.4	4.69
3	4a	12.5	13.9	13.3	11.0§	1.68
3	4b	52	5.8	24.1	28.0	1.99
4	5a	-0.2	-2	-1.3	-0.77	1.41
4	5b	0.7	2.9	2.0	1.6§	1.69
5	6a	-0.3	-0.3	-0.3	-0.06	3.4
5	6b	1.5	3.8	2.9	2.1	3.81

§ Overlaying signals. J_{HF} coupling extracted manually from IPAP data using TopSpin software.

Table S23: J_{HF} values from DFT calculations and extracted from experimental IPAP data (Figure S12) for *cis*-fluorohydrin, in D_2O. From DFT calculations, the ratio of ax-eq conformer: eq-ax conformer was 18.7:81.3.

		***cis* isomer and its conformations**				
		**$^nJ_{HF}$ in water**				
**$^nJ_{H_XF}$**		**Calculated with DFT / Hz**			**Measured with IPAP-FESTA / Hz**	**^{1}H NMR (δ / ppm)**
**n**	**X**	**(*cis* ax-eq)**	**(*cis* eq-ax)**	**(weighted average)**		
2	3	54.1	56	55.6	48.5	4.60
3	2	0.9	18.3	15.0	14.7	4.89
3	4a	6.8	51.7	43.3	32.7§	1.81
3	4b	9.9	11.5	11.2	10.5	2.01
4	5a	4.5	0.7	1.4	1.9§	1.71
4	5b	-2.2	0.3	-0.2	0.8§§	1.47
5	6a	4.5	1.2	1.8	1.9	3.56
5	6b	-0.4	-0.1	-0.2	-0.02	3.87

§ Overlaying signals. J_{HF} coupling extracted manually from IPAP data using TopSpin software.

§§ $^4J_{5bF}$ measured via HD-HAPPY-FESTA due to severe signal overlap.

Table S24: J_{HF} values from DFT calculations and extracted from experimental IPAP data (Figure S13) for *trans*-fluorohydrin, in D_2O. From DFT calculations, the ratio of ax-ax conformer: eq-eq conformer was 18.4:81.6.

		***trans* isomer and its conformations**				
		**$^nJ_{HF}$ in water**				
**$^nJ_{H_XF}$**		**Calculated with DFT / Hz**			**Measured with IPAP-FESTA / Hz**	**^{1}H NMR (δ / ppm)**
**n**	**X**	***trans*(ax-ax)**	***trans*(eq-eq)**	**(weighted average)**		
2	3	53	57.6	56.8	48.5	4.31
3	2	10.7	3.3	4.7	4.9	4.82
3	4a	12.1	13.7	13.4	11.4	1.72
3	4b	52	5.8	14.3	23.3	2.06
4	5a	-0.1	-2	-1.7	-0.91	1.51
4	5b	0.7	3.1	2.7	2.4§	1.75
5	6a	-0.3	-0.2	-0.2	-0.07	3.51
5	6b	1.5	3.7	3.3	2.4	3.87

§ $^4J_{5bF}$ value is approximated using HD-HAPPY-FESTA data due to severe peak overlap.

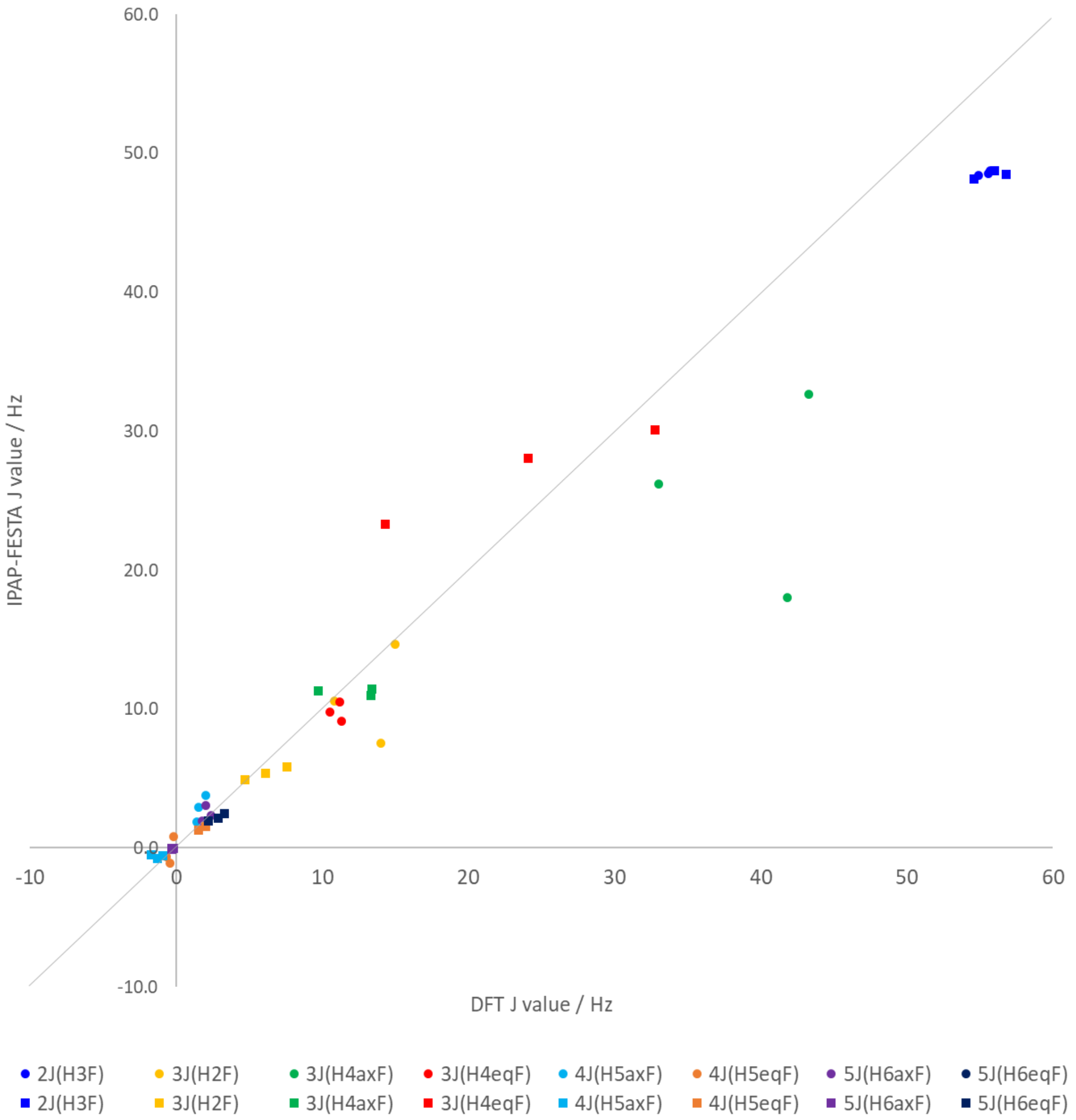

Figure S16: Scattergram comparing calculated DFT and measured IPAP-FESTA J_{HF} values for *cis*-fluorohydrin (circle data plots) and *trans*-fluorohydrin (square data plots) in $CDCl_3$, D_2O and DMSO. The trend line represents exact agreement between simulated and experimental *J* values. R^2 for all data points is 0.95.

3.5. Repeatability of J_{HF} couplings measured from experimental IPAP-FESTA data

To confirm the repeatability of IPAP-FESTA for measuring the magnitude and sign of J_{HF} couplings, ten repetitions of IPAP-FESTA data acquisition and processing were performed for *cis*-fluorohydrin in DMSO-d_6, for eight multiplets. The data were processed with two different time-domain weighting functions (no weighting, and 1 Hz worth of exponential weighting), using a modified version of the Mathematica notebook in Section 5. The results are summarised in in Tables S25 and S26. The standard deviations of the 16 J_{HF} coupling values ranged from 0.3 to 1.1 mHz. This confirms the excellent repeatability of the IPAP-FESTA data processing, potentially giving mHz accuracy in the weak coupling limit. Importantly, IPAP methods will show systematic errors in the presence of strong couplings; these have not been analysed and will be spin system dependent. The repeatability data and the Mathematica notebook used are available from the DOI: 10.48420/21975908.

Table S25: IPAP-FESTA repeatability results for the 10 repetitions of *cis*-fluorohydrin in DMSO-d_6, where no time-domain weighting function is applied.

Chemical shift / ppm	Measured J_{HF} coupling / Hz	Standard deviation of J_{HF}
4.90	7.599	0.0011
4.45	48.724	0.0010
3.75	-0.120	0.0014
3.38	3.028	0.0009
1.91	9.115	0.0001
1.76	18.057	0.0025
1.63	2.943	0.0032
1.49	-1.130	0.0008

Table S26: IPAP-FESTA repeatability results for the 10 repetitions of *cis*-fluorohydrin in DMSO-d$_6$, where 1 Hz worth of time-domain exponential weighting is applied.

Chemical shift / ppm	Measured J_{HF} coupling / Hz	Standard deviation of J_{HF}
4.90	7.583	0.0009
4.45	48.727	0.0002
3.75	-0.120	0.0011
3.38	3.034	0.0007
1.91	9.111	0.0013
1.76	18.006	0.0017
1.63	2.927	0.0012
1.49	-1.116	0.0005

4. Product operator description of the MODO element in IPAP-FESTA

To demonstrate how the modulated echo selectively excites a single ^{19}F coupled ^{1}H multiplet, a four-spin system is described by product operators using POMA,[7] where spins 1 and 2 are ^{1}H, and spins 3 and 4 are ^{19}F. All spins are mutually coupled, and the selected ^{1}H-^{19}F coupled pair are spins 1 and 3. A simplified version of the modulated echo in IPAP-FESTA is shown in Figure S17. For each set of data (IP and AP), the odd- and even-numbered scans yield identical product operators and therefore combine constructively on summation of successive scans. For spin 1 (the selected ^{1}H), the effects of the *J*-coupling to spins 2 and 4 are refocused. A summary of the Δ delays used, and the resulting product operators, is given in Table S26. The Mathematica notebook used to determine the product operators resulting for each scan is available from the DOI: 10.48420/21975908.

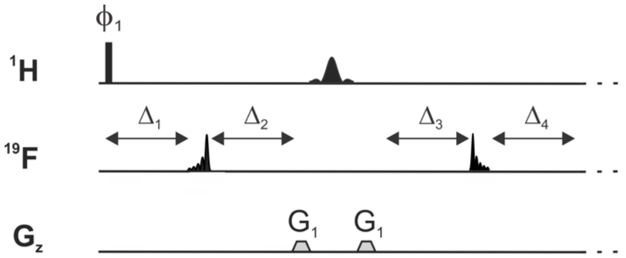

Figure S17: A simplified pulse sequence of the selectively modulated echo in IPAP-FESTA. IP and AP, modulated and unmodulated, data are acquired by using different arrangements of ^{19}F selective iBURP pulses and delays. Modulated and unmodulated data are acquired by changing the phase of the excitation 90° ^{1}H pulse; ϕ_1= x on odd-numbered scans, ϕ_1= –x on even-numbered scans and using different arrangements of ^{19}F selective iBURP pulses and delays in odd- and even-numbered scans. The values of Δ_1, Δ_2, Δ_3 and Δ_4 are dependent on whether IP, AP, modulated or unmodulated data is being acquired. Refer to Table S24 for specific durations for each scan in IPAP-FESTA.

Table S27: Summary of the resulting product operator in odd- and even-numbered scans in the IP and AP data sets, after the selective modulated echo shown in Figure S17. $J_{HF}$is the *J*-coupling between the chosen ^{1}H-^{19}F coupled pair (spins 1 and 3). The delays Δ_1, Δ_2, Δ_3 and Δ_4 are shown in Figure S17.

IP or AP data acquisition	Odd / even numbered scan	Delta (Δ) durations / s				Product Operator
		Δ_1	Δ_2	Δ_3	Δ_4	
IP	odd	0	$1/2J_{HF}$	0	$1/2J_{HF}$	$-\hat{H}_{1y}$
	even	$1/2J_{HF}$	0	0	$1/2J_{HF}$	$-\hat{H}_{1y}$
AP	odd	0	$1/2J_{HF}$	$1/4J_{HF}$	$1/4J_{HF}$	$-2\hat{H}_{1x}\hat{F}_{3z}$
	even	$1/4J_{HF}$	$1/4J_{HF}$	$1/2J_{HF}$	0	$-2\hat{H}_{1x}\hat{F}_{3z}$

5. Mathematica code for determining J_{HF} coupling constants from IPAP-FESTA data

```
Clear[Evaluate[Context[] <> "*"]];
vardiff[tspip_,tspap_,ratio_?NumericQ,diff_?NumericQ]:=Module[{zspm,len},len=Length[tspip];
  zspm=Join[Table[0,{i,len}],Join[tspip-ratio tspap,Table[0,{i,len}]]];
  Total[(tspip+ratio tspap-Take[RotateRight[zspm,diff],{len+1,2 len}])^2]
  ]; (* Calculate variance between shifted spectra obtained by IPAP processing *)

getpars[filenamebase_,procno_]:=Module[{filename},filename=filenamebase<>"/pdata/"<>procno<>"/procs";
 stream=OpenRead[filename];
 offset=Read[StringToStream[Find[stream,"OFFSET= "]],{Word,Number}][[2]];
 si=Read[StringToStream[Find[stream,"SI= "]],{Word,Number}][[2]];
 Close[stream];
 filename=filenamebase<>"/acqus";
 stream=OpenRead[filename];
 sfo1=Read[StringToStream[Find[stream,"SFO1= "]],{Word,Number}][[2]];
 swp=Read[StringToStream[Find[stream,"SW= "]],{Word,Number}][[2]];
 Close[stream];
 {offset,si,sfo1,swp}
 ]

process[filenamebaseIP_,filenamebaseAP_,shift_,width_,swp_,offset_,si_,sfo1_]:=Module[{diff,dip,start,end,hzpp,ncproc,spip,s
pap},
  filename=filenamebaseIP<>"/procs";
  stream=OpenRead[filename];
  ncproc=Read[StringToStream[Find[stream,"NC_proc= "]],{Word,Number}][[2]]; (* Need to correct for any difference in scaling
during FT etc. *)
  Close[stream];
  filename=filenamebaseIP<>"/1r";
  spip=2.^ncproc Import[filename,"Integer32"]/10^6;

  filename=filenamebaseAP<>"/procs";
  stream=OpenRead[filename];
  ncproc=Read[StringToStream[Find[stream,"NC_proc= "]],{Word,Number}][[2]]; (* Need to correct for any difference in scaling
during FT etc. *)
  Close[stream];
  filename=filenamebaseAP<>"/1r";
  spap=2.^ncproc Import[filename,"Integer32"]/10^6;

  hzpp=swp sfo1/(si-1);
  start=Round[sfo1 (offset-shift-width/2)/hzpp];
  end=Round[sfo1 (offset-shift+width/2)/hzpp ];
  tspip=Take[spip,{start,end}];
  len=Length[tspip];
  tspap=Take[spap,{start,end}];
  Print[Style["Chemical shift = ","Subsection"],Style[shift,"Subsection"],Style[" ppm","Subsection"]];
  (* Do optimization in two stages, otherwise fails to find global minimum *)
  res1=NMinimize[{vardiff[tspip,tspap,fratio,5 fdiff],fdiff>-len/5,fdiff<len/5,fratio>0.8,fratio<1.2},{fdiff∈
Integers,fratio},MaxIterations->100];
  res2=NMinimize[{vardiff[tspip,tspap,fratio, fdiff],fratio>0.8,fratio<1.2,fdiff>Round[(5 fdiff/.res1[[2]])]-
len/10,fdiff<Round[len/10+5 fdiff/.res1[[2]]]},{fdiff∈ Integers,fratio},MaxIterations->1000];
  Print["Variance and optimized shift and ratio:"];
  Print[res2];
  ratio=fratio/.res2[[2]];
  spp=tspip+ratio tspap;
  spm=tspip-ratio tspap;
```

```
  Print[Style["Input data","Subsection"]];
  Print[ListPlot[{spp,spm},Joined->True,PlotRange->All]];
  len=Length[spp];
  zspm=Join[Table[0,{i,len}],Join[spm,Table[0,{i,len}]]];
  diff=Table[0,{i,2 len+1}];
  For[i=1,i<=Length[diff],i++,
   diff[[i]]=Total[(spp-Take[RotateRight[zspm,len-i],{len+1,2 len}])^2];
   ];
  Print[Style["RMS difference vs displacement in data points","Subsection"]];
  Print[ListPlot[diff,Joined->True,PlotRange->All]];
  min=Min[diff];
  For[i=1,i<=Length[diff],i++, If[diff[[i]]==min,minpoint=i];];
  Print[Style["Expansion of RMS difference vs displacement in data points, with parabolic fit","Subsection"]];
  dip=Take[diff,{minpoint-10,minpoint+10}];
  dipfit=NonlinearModelFit[dip,a+b (x-x0)^2,{a,b,x0},x];
  Print[Show[{Plot[dipfit[x],{x,1,Length[dip]},PlotRange->{All,{0,1.1 Max[dip]}}],ListPlot[dip,Joined->False,PlotRange->All]}]];
  Print["Interpolated frequency shift with best match is ",-hzpp((x0/.dipfit["BestFitParameters"])+minpoint-11-len), " Hz"];
  -hzpp((x0/.dipfit["BestFitParameters"])+minpoint-11-len)
  ];
(* Enter experimental information here *)
shifts={5.5};
widths={0.3};
njs=Length[shifts];
nreps=1;
filestart=10;
js=Table[0,njs,nreps];
filenamebase="W:/FILEPATH/";
procnoIP="1001";
procnoAP="1002";
(* *)

getpars[filenamebase<>ToString[filestart],procnoIP];
starttime=AbsoluteTime[];
For[jnumber=1,jnumber<=Length[shifts],jnumber++,
 For[rep=1,rep<=nreps,rep++,
   filename=filenamebase<>ToString[filestart+rep-1]<>"/pdata/";
   js[[jnumber,rep]]=process[filename<>procnoIP,filename<>procnoAP,shifts[[jnumber]],widths[[jnumber]],swp,offset,si,sfo1];
   ];
 ];
Print[Style["Coupling constants","Subsection"]];
TableForm[js]
jslb1=js;
timeused=AbsoluteTime[]-starttime;
Print["Time used = ",timeused,"seconds"];
meansdandsdmean[x_]:={Mean[x],Sqrt[Variance[x]/((Length[x]-1))],Sqrt[Variance[x]/(Length[x] (Length[x]-1))]};
If[nreps>1,reslb1=Table[0,Length[shifts]];
 reslb1=Prepend[Table[Flatten[{shifts[[i]],meansdandsdmean[jslb1[[i]]]}],{i,Length[shifts]}],{"Shift / ppm","JHF / Hz","std dev / Hz","sd of mean of J / Hz"}];
 Print[TableForm[reslb1]];]
```

6. Pulse program codes for Bruker spectrometers

6.1. IPAP-MODO-FESTA pulse program suitable for a TBI probe

```
; ipap_modofesta_tbi
; optional IPAP using ZGOPTNS
;
;   Developed by:
;   NMR Methodology Group
;   University of Manchester
;   Physical Organic Chemistry Laboratory (LFQO)
;   University of Campinas - Brazil
;
;   IPAP-pMODO-FESTA
;   1D homonuclear Hartman-Hahn transfer using
;   DIPSI2 sequence for mixing
;   permuted modulated heteronuclear
;   spin echo with gradients
;   using selective refocussing/inversion pulses
;
; FESTA:
; L. Castanar, P. Moutzouri, T. Barbosa, C. Tormena, R. Rittner, A. Phillips, S. Coombes, M. Nilsson, G. A. Morris.
; Anal. Chem., 2018, 90, 8, 5445-5450
; DOI: 10.1021/acs.analchem.8b00753
;T. M. Barbosa, L. Castanar, P. Moutzouri, M. Nilsson, G. A. Morris, R. Rittner, C. F. Tormena
; Anal. Chem. 2020, 92, 2, 2224–2228
; DOI: 10.1021/acs.analchem.9b04924
; G. Dal Poggetto, J. V. Soares, C. F. Tormena
; Anal. Chem. 2020, 92, 20, 14047–1405
; DOI: 10.1021/acs.analchem.0c02976

;   THE USE OF WAVEMAKER IS RECOMMENDED
;   ZGOPTNS:
;   -DIP : In-phase data set
;   -DAP : Anti-phase data set
;   -DIPAP : Both In-phase and Anti-phase interleaved data sets (2D, TD=2)
;
;   Other option
;           [cnst9 = 1] with TOCSY transfer
;           [cnst9 = 0] without TOCSY transfer
;$CLASS=HighRes
;$DIM=2D
;$TYPE=
;$SUBTYPE=
;$COMMENT=

#include <Avance.incl>
#include <Grad.incl>
#include <Delay.incl>
#include <De.incl>

"p2=2*p1"
"p4=p3*2"
"p18=1m"

;%%%%%%%%%% WaveMaker Parameters %%%%%%%%%%
;sp12:wvm:IPAP_rsnob:f1 rsnob(cnst12 Hz, cnst13 ppm; PA=0.5; NPOINTS=5000) ss=1 us;
;sp14:wvm:IPAP_Fiburp1:f2 iburp2(cnst14 Hz, cnst15 ppm; PA=1; NP=5000) ss=1 us;
;sp15:wvm:IPAP_Fiburp2:f2 iburp2(cnst14 Hz, cnst15 ppm; TR=1; NP=5000) ss=1 us;

"d11=30m+1s/(cnst12)-1s/(cnst12)"
"d11=30m+1s/(cnst13)-1s/(cnst13)"
"d11=30m+1s/(cnst14)-1s/(cnst14)"
"d11=30m+1s/(cnst15)-1s/(cnst15)"
```

```
;%%%%%%%%%% TOCSY Parameters %%%%%%%%%%
"p33=1000000.0/(cnst53*4)"
"cnst33= (p33/p1) * (p33/p1)"
"spw32=plw1/cnst33"

"p35=1000000.0/(cnst54*4)"
"cnst35= (p35/p1) * (p35/p1)"
"spw34=plw1/cnst35"

"spoff32=0"
"spoff34=0"

"p6=1000000.0/(cnst6*4)"
"cnst46= (p6/p1) * (p6/p1)"
"plw10=plw1/cnst46"

"FACTOR1=(d9/(p6*115.112))/2"
"l1=FACTOR1*2"

;%%%%%%%%%% Other Parameters %%%%%%%%%%
"d4=((1s*0.5)/(cnst2*cnst3))"

"DELTA1=d4*0.5"

"spoal12=0.5"
"spoal14=1"
"spoal15=0"

"d11=30m"
"d12=20u"

#ifdef IP
"l0=1"
#endif

#ifdef AP
"l0=0"
#endif

#ifdef IPAP
"l0=1"
#endif

"acqt0=-p1*2/3.1416"

1 ze
  d11 pl2:f2
2 d11
  50u LOCKH_OFF
  d1
  50u LOCKH_ON
  10u UNBLKGRAMP
  d12 pl1:f1
3 d12

if "l0%2 == 1"          ;In-Phase
                {
if "nsdone%2 == 1"
   {
  (p1 ph1):f1

  20u pl3:f2
  (p14:sp14 ph3):f2
  20u pl0:f1

  DELTA1
  DELTA1
```

```
  p16:gp1
  d16
  (p12:sp12 ph2):f1
  p16:gp1
  d16

  20u pl3:f2
  (p14:sp15 ph3):f2
  20u pl1:f1

  DELTA1
  DELTA1

  (p1 ph4):f1
}
else
  {
  (p1 ph1+ph11):f1

  DELTA1
  DELTA1

  20u pl3:f2
  (p14:sp14 ph3):f2
  20u pl0:f1

  p16:gp1
  d16
  (p12:sp12 ph2):f1
  p16:gp1
  d16

  20u pl3:f2
  (p14:sp15 ph3):f2
  20u pl1:f1

  DELTA1
  DELTA1

  (p1 ph4):f1
}
                } ;END In-Phase
                else ;Anti-phase
                {
 if "nsdone%2 == 1"
    {
    (p1 ph1):f1

    20u pl3:f2
    (p14:sp14 ph3):f2
    20u pl0:f1

    DELTA1
    DELTA1

    p16:gp1
    d16
    (p12:sp12 ph2):f1
    p16:gp1
    d16

    DELTA1

    20u pl3:f2
    (p14:sp15 ph3):f2
    20u pl1:f1
```

```
   DELTA1

  (p1 ph5):f1
}
  else
   {
   (p1 ph1+ph11):f1

   DELTA1

   20u pl3:f2
   (p14:sp14 ph3):f2
   20u pl0:f1

   DELTA1

   p16:gp1
   d16
   (p12:sp12 ph2):f1
   p16:gp1
   d16

   DELTA1
   DELTA1

   20u pl3:f2
   (p14:sp15 ph3):f2
   20u pl1:f1

  (p1 ph5):f1
}
                } ;END Anti-phase

  10u pl0:f1
  10u gron0
  (p32:sp32 ph6):f1
  20u groff

  100u pl10:f1

  if "cnst9 == 1"    ;TOCSY block
  {
                                                          ;begin DIPSI2
4 p6*3.556 ph23
  p6*4.556 ph25
  p6*3.222 ph23
  p6*3.167 ph25
  p6*0.333 ph23
  p6*2.722 ph25
  p6*4.167 ph23
  p6*2.944 ph25
  p6*4.111 ph23
  p6*3.556 ph25
  p6*4.556 ph23
  p6*3.222 ph25
  p6*3.167 ph23
  p6*0.333 ph25
  p6*2.722 ph23
  p6*4.167 ph25
  p6*2.944 ph23
  p6*4.111 ph25
  p6*3.556 ph25
  p6*4.556 ph23
  p6*3.222 ph25
  p6*3.167 ph23
  p6*0.333 ph25
```

```
  p6*2.722 ph23
  p6*4.167 ph25
  p6*2.944 ph23
  p6*4.111 ph25
  p6*3.556 ph23
  p6*4.556 ph25
  p6*3.222 ph23
  p6*3.167 ph25
  p6*0.333 ph23
  p6*2.722 ph25
  p6*4.167 ph23
  p6*2.944 ph25
  p6*4.111 ph23
  lo to 4 times l1
                                                  ;end DIPSI2
}
else
{
}
  p18:gp2*-1

  d18 pl0:f1
  10u gron10
  (p34:sp34 ph6):f1
  20u groff

  100u pl1:f1
  200u BLKGRAMP
if "l0%2 == 1"          ;In-Phase
                {
  20u pl1:f1 pl2:f2
 (center (p1 ph7):f1 (p3 ph8):f2 )
}
else
{
  20u
  (p1 ph7):f1
}
  go=2 ph31
  d11 mc #0 to 2

#ifdef IPAP
  F1QF(calclc(l0,1))
#else
  F1QF()
#endif

  50u LOCKH_OFF
exit

ph1=0 0 0 0 0 0 0 0 2 2 2 2 2 2 2 2
ph11=2
ph2=0 0 1 1 2 2 3 3
ph3=0
ph4=0 0 0 0 0 0 0 0 0 0 0 0 0 0 0 0 2 2 2 2 2 2 2 2 2 2 2 2 2 2 2 2
ph5=1 1 1 1 1 1 1 1 1 1 1 1 1 1 1 1 3 3 3 3 3 3 3 3 3 3 3 3 3 3 3 3
ph6=0
ph7=0 0 0 0 0 0 0 0 0 0 0 0 0 0 0 0 0 0 0 0 0 0 0 0 0 0 0 0 0 0 0 0 2 2 2 2 2 2 2 2 2 2 2 2 2 2 2 2 2 2 2 2 2 2 2 2 2 2 2 2 2 2 2 2
ph8=0 2 2 0
ph23=3
ph25=1
ph31=0 0 2 2 0 0 2 2 2 2 0 0 0 0 2 2 2 2 0 0 2 2 0 0 0 0 2 2 2 2 0 0 2 2 0 0 2 2 0 0 0 0 2 2 2 2 0 0 0 0 2 2 0 0 2 2 2 2 0 0 0 0 2 2

;POWER LEVEL
;pl0  : f1 channel - zero power (0W)
;pl1  : f1 channel - power level for hard pulse (default)
;pl2  : f2 channel - power level for hard pulse (default)
```

```
;pl3  : f2 channel - zero power (0W)
;pl10 : f1 channel - power level for TOCSY-spinlock
;sp12 : f1 channel - power level of refocusing shaped pulse
;sp14 : f2 channel - power level of inversion shaped pulse
;sp15 : f2 channel - power level of inversion shaped pulse
;sp32 : f1 channel - power level of adiabatic pulse of first ZQF element
;sp34 : f1 channel -power level of adiabatic pulse of last ZQF element

;PULSE DURATION
;p1  : f1 channel - 90 degree high power pulse
;p2  : f1 channel - 180 degree high power pulse
;p3  : f2 channel - 90 degree high power pulse
;p4  : f2 channel - 180 degree high power pulse
;p6  : f1 channel - 90 degree low power pulse
;p12 : f1 channel - 180 degree refocusing shaped pulse
;p14 : f2 channel - 180 degree inversion shaped pulse
;p32 : f1 channel - first ZQF 180 degree inversion shaped pulse (adiabatic)          [10 msec]
;p34 : f1 channel - second ZQF 180 degree inversion shaped pulse (adiabatic)         [30 msec]

;GRADIENT DURATION
;p16 : duration of CTP gradients [200 us - 1 ms]
;p18 : homospoil gradient [1 msec]

;DELAY
;d1  : relaxation delay;                                  [5*T1 s]
;d4  : delay for JHX evolution
;d9  : TOCSY mixing time                                  [50-200 ms]
;d11 : delay for disk I/O                                 [30 msec]
;d16 : recovery delay for gradients                       [200 us - 1 ms ]
;d18 : recovery delay for homospoil gradient              [200 us - 1 ms ]

;PULSE SHAPE
;spnam12 : f1 channel - file name for the selective 180 refocusing shaped pulse
          [RSNOB]
;spnam14 : f2 channel - file name for the selective 180 inversion shaped pulse            [IBURP2]
;spnam15 : f2 channel - file name for the selective 180 inversion shaped pulse            [IBURP2]
;spnam32 : f1 channel - file name for the adiabatic shaped pulse using in first ZQF       [CHIRP]
;       smoothed chirp (low to high, 20% smoothing, 1000 points, 20KHz)
;spnam34 : f1 channel - file name for the adiabatic shaped pulse using in last ZQF        [CHIRP]
;       smoothed chirp (low to high, 20% smoothing, 1000 points, 20KHz)

;GRADIENT SHAPE
;gpnam1 : SMSQ10.100
;gpnam2 : SMSQ10.100

;GRADIENT STRENGTH
;gpz0 : first ZQF gradient              [3%]
;gpz1 : CTP gradient                    [23%]
;gpz2 : spoil gradient                  [79%]
;gpz10: last ZQF gradient               [4%]

;OTHER
;cnst2 : selected J(HX)(in Hz)
;cnst3 : 1 for AX/AX3 and 2 for AX2
;cnst6: bandwidth of TOCSY transfer (in Hz)
;cnst9: 1 (with DIPSI-2) / 0 (without DIPSI-2)
;cnst12: f1 channel - bandwidth for selective refocussing pulse (in Hz)
;cnst13: f1 channel - chemical shift for selective refocussing pulse (in ppm)
;cnst14: f2 channel - bandwidth for selective inversion pulse (in Hz)
;cnst15: f2 channel - chemical shift for selective inversion pulse (in ppm)
;cnst53 : GammaB1 of first adiabatic ZQF shaped pulse
;cnst54 : GammaB1 of last adiabatic ZQF shaped pulse
;l1   : loop for DIPSI cycle: ((p6*115.112) * l1) = mixing time
;ns: 16 * n, total number of scans: NS * TD0
;ds: 8
;MC2 : QF
```

6.2. IPAP-MODO-FESTA pulse program suitable for a BBFO probe

```
; ipap_modofesta_bbfo
; optional IPAP using ZGOPTNS
;
;   Coral Mycroft and Guilherme Dal Poggetto
;   email: coral.mycroft@manchester.ac.uk and
;          guilherme.dal.poggetto@merck.com
;
;   Developed by:
;   NMR Methodology Group
;   University of Manchester
;   Physical Organic Chemistry Laboratory (LFQO)
;   University of Campinas - Brazil
;
;   IPAP-pMODO-FESTA
;   1D homonuclear Hartman-Hahn transfer using
;   DIPSI2 sequence for mixing
;   permuted modulated heteronuclear
;   spin echo with gradients
;   using selective refocussing/inversion pulses
;
; FESTA:
; L. Castanar, P. Moutzouri, T. Barbosa, C. Tormena, R. Rittner, A. Phillips, S. Coombes, M. Nilsson, G. A. Morris.
; Anal. Chem., 2018, 90, 8, 5445-5450
; DOI: 10.1021/acs.analchem.8b00753
;T. M. Barbosa, L. Castanar, P. Moutzouri, M. Nilsson, G. A. Morris, R. Rittner, C. F. Tormena
; Anal. Chem. 2020, 92, 2, 2224–2228
; DOI: 10.1021/acs.analchem.9b04924
;G. Dal Poggetto, J. V. Soares, C. F. Tormena
; Anal. Chem. 2020, 92, 20, 14047–1405
; DOI: 10.1021/acs.analchem.0c02976
;
;   THE USE OF WAVEMAKER IS RECOMMENDED
;   ZGOPTNS:
;   -DIP : In-phase data set
;   -DAP : Anti-phase data set
;   -DIPAP : Both In-phase and Anti-phase interleaved data sets (2D, TD=2)
;
;   Other option
;          [cnst9 = 1] with TOCSY transfer
;          [cnst9 = 0] without TOCSY transfer
;$CLASS=HighRes
;$DIM=2D
;$TYPE=
;$SUBTYPE=
;$COMMENT=

#include <Avance.incl>
#include <Grad.incl>
#include <Delay.incl>
#include <De.incl>

"p2=2*p1"
"p4=p3*2"
"p18=1m"

;%%%%%%%%%% WaveMaker Parameters %%%%%%%%%%
;sp12:wvm:IPAP_rsnob:f1 rsnob(cnst12 Hz, cnst13 ppm; PA=0.5; NPOINTS=5000) ss=1 us;
;sp14:wvm:IPAP_Fiburp1:f2 iburp2(cnst14 Hz, cnst15 ppm; PA=1; NP=5000) ss=1 us;
;sp15:wvm:IPAP_Fiburp2:f2 iburp2(cnst14 Hz, cnst15 ppm; TR=1; NP=5000) ss=1 us;

"d11=30m+1s/(cnst12)-1s/(cnst12)"
"d11=30m+1s/(cnst13)-1s/(cnst13)"
"d11=30m+1s/(cnst14)-1s/(cnst14)"
"d11=30m+1s/(cnst15)-1s/(cnst15)"
```

```
;%%%%%%%%% TOCSY Parameters %%%%%%%%%
"p33=1000000.0/(cnst53*4)"
"cnst33= (p33/p1) * (p33/p1)"
"spw32=plw1/cnst33"

"p35=1000000.0/(cnst54*4)"
"cnst35= (p35/p1) * (p35/p1)"
"spw34=plw1/cnst35"

"spoff32=0"
"spoff34=0"

"p6=1000000.0/(cnst6*4)"
"cnst46= (p6/p1) * (p6/p1)"
"plw10=plw1/cnst46"

"FACTOR1=(d9/(p6*115.112))/2"
"l1=FACTOR1*2"

;%%%%%%%%% Other Parameters %%%%%%%%%
"d4=((1s*0.5)/(cnst2*cnst3))"

"DELTA=d4*0.5"

"spoal12=0.5"
"spoal14=1"
"spoal15=0"

"d11=30m"
"d12=20u"

#ifdef IP
"l0=1"
#endif

#ifdef AP
"l0=0"
#endif

#ifdef IPAP
"l0=1"
#endif

"acqt0=-p1*2/3.1416"

1 ze
  d11 pl2:f2
2 d11
  50u LOCKH_OFF
  d1
  50u LOCKH_ON
  10u UNBLKGRAMP
  d12 pl1:f1
3 d12

if "l0%2 == 1"          ;In-Phase
                {
if "nsdone%2 == 1"
   {
  (p1 ph1):f1

  20u pl3:f2
  (p14:sp14 ph3):f2
  20u pl0:f1

  DELTA
```

```
  DELTA

  p16:gp1
  d16
  (p12:sp12 ph2):f1
  p16:gp1
  d16

  20u pl3:f2
  (p14:sp15 ph3):f2
  20u pl1:f1

  DELTA
  DELTA

  (p1 ph4):f1
}
else
  {
  (p1 ph1+ph11):f1

  DELTA
  DELTA

  20u pl3:f2
  (p14:sp14 ph3):f2
  20u pl0:f1

  p16:gp1
  d16
  (p12:sp12 ph2):f1
  p16:gp1
  d16

  20u pl3:f2
  (p14:sp15 ph3):f2
  20u pl1:f1

  DELTA
  DELTA

  (p1 ph4):f1
}
                } ;END In-Phase
                else ;Anti-phase
                {
 if "nsdone%2 == 1"
   {
   (p1 ph1):f1

   20u pl3:f2
   (p14:sp14 ph3):f2
   20u pl0:f1

   DELTA
   DELTA

   p16:gp1
   d16
   (p12:sp12 ph2):f1
   p16:gp1
   d16

   DELTA

   20u pl3:f2
   (p14:sp15 ph3):f2
```

```
   20u pl1:f1

   DELTA

  (p1 ph5):f1
}
  else
   {
   (p1 ph1+ph11):f1

   DELTA

   20u pl3:f2
   (p14:sp14 ph3):f2
   20u pl0:f1

   DELTA

   p16:gp1
   d16
   (p12:sp12 ph2):f1
   p16:gp1
   d16

   DELTA
   DELTA

   20u pl3:f2
   (p14:sp15 ph3):f2
   20u pl1:f1

  (p1 ph5):f1
}
                } ;END Anti-phase
  10u pl0:f1
  10u gron0
  (p32:sp32 ph6):f1
  20u groff
  100u pl10:f1

  if "cnst9 == 1"    ;TOCSY block
  {
                                                            ;begin DIPSI2
4 p6*3.556 ph23
  p6*4.556 ph25
  p6*3.222 ph23
  p6*3.167 ph25
  p6*0.333 ph23
  p6*2.722 ph25
  p6*4.167 ph23
  p6*2.944 ph25
  p6*4.111 ph23
  p6*3.556 ph25
  p6*4.556 ph23
  p6*3.222 ph25
  p6*3.167 ph23
  p6*0.333 ph25
  p6*2.722 ph23
  p6*4.167 ph25
  p6*2.944 ph23
  p6*4.111 ph25
  p6*3.556 ph25
  p6*4.556 ph23
  p6*3.222 ph25
  p6*3.167 ph23
  p6*0.333 ph25
  p6*2.722 ph23
```

```
  p6*4.167 ph25
  p6*2.944 ph23
  p6*4.111 ph25
  p6*3.556 ph23
  p6*4.556 ph25
  p6*3.222 ph23
  p6*3.167 ph25
  p6*0.333 ph23
  p6*2.722 ph25
  p6*4.167 ph23
  p6*2.944 ph25
  p6*4.111 ph23
  lo to 4 times l1
                                                                              ;end DIPSI2
}
else
{
}
  p18:gp2*-1
  d18 pl0:f1
  10u gron10
  (p34:sp34 ph6):f1
  20u groff

  100u pl1:f1
  200u BLKGRAMP
if "l0%2 == 1"          ;In-Phase
          {
  20u pl1:f1 pl2:f2
(p3 ph8):f2
(p1 ph7):f1
}
else
{
  20u
  (p1 ph7):f1
}
  go=2 ph31
  d11 mc #0 to 2

#ifdef IPAP
  F1QF(calclc(l0,1))
#else
  F1QF()
#endif

  50u LOCKH_OFF
exit

ph1=0 0 0 0 0 0 0 0 2 2 2 2 2 2 2 2
ph11=2
ph2=0 0 1 1 2 2 3 3
ph3=0
ph4=0 0 0 0 0 0 0 0 0 0 0 0 0 0 0 0 2 2 2 2 2 2 2 2 2 2 2 2 2 2 2 2
ph5=1 1 1 1 1 1 1 1 1 1 1 1 1 1 1 1 3 3 3 3 3 3 3 3 3 3 3 3 3 3 3 3
ph6=0
ph7=0 0 0 0 0 0 0 0 0 0 0 0 0 0 0 0 0 0 0 0 0 0 0 0 0 0 0 0 0 0 0 0 2 2 2 2 2 2 2 2 2 2 2 2 2 2 2 2 2 2 2 2 2 2 2 2 2 2 2 2 2 2 2 2
ph8=0 2 2 0
ph23=3
ph25=1
ph31=0 0 2 2 0 0 2 2 2 2 0 0 0 0 2 2 2 2 0 0 2 2 0 0 0 0 2 2 2 2 0 0 2 2 0 0 2 2 0 0 0 0 2 2 2 2 0 0 0 0 2 2 0 0 2 2 2 2 0 0 0 0 2 2

;POWER LEVEL
;pl0  : f1 channel - zero power (0W)
;pl1  : f1 channel - power level for hard pulse (default)
;pl2  : f2 channel - power level for hard pulse (default)
;pl3  : f2 channel - zero power (0W)
```

```
;pl10 : f1 channel - power level for TOCSY-spinlock
;sp12 : f1 channel - power level of refocusing shaped pulse
;sp14 : f2 channel - power level of inversion shaped pulse
;sp15 : f2 channel - power level of inversion shaped pulse
;sp32 : f1 channel - power level of adiabatic pulse of first ZQF element
;sp34 : f1 channel -power level of adiabatic pulse of last ZQF element

;PULSE DURATION
;p1  : f1 channel - 90 degree high power pulse
;p2  : f1 channel - 180 degree high power pulse
;p3  : f2 channel - 90 degree high power pulse
;p4  : f2 channel - 180 degree high power pulse
;p6  : f1 channel - 90 degree low power pulse
;p12 : f1 channel - 180 degree refocusing shaped pulse
;p14 : f2 channel - 180 degree inversion shaped pulse
;p32 : f1 channel - first ZQF 180 degree inversion shaped pulse (adiabatic)          [10 msec]
;p34 : f1 channel - second ZQF 180 degree inversion shaped pulse (adiabatic)         [30 msec]

;GRADIENT DURATION
;p16 : duration of CTP gradients [200 us - 1 ms]
;p18 : homospoil gradient [1 msec]

;DELAY
;d1  : relaxation delay;                                  [5*T1 s]
;d4  : delay for JHX evolution
;d9  : TOCSY mixing time                                  [50-200 ms]
;d11 : delay for disk I/O                                 [30 msec]
;d16 : recovery delay for gradients                       [200 us - 1 ms ]
;d18 : recovery delay for homospoil gradient              [200 us - 1 ms ]

;PULSE SHAPE
;spnam12 : f1 channel - file name for the selective 180 refocusing shaped pulse
        [RSNOB]
;spnam14 : f2 channel - file name for the selective 180 inversion shaped pulse                  [IBURP2]
;spnam15 : f2 channel - file name for the selective 180 inversion shaped pulse                  [IBURP2]
;spnam32 : f1 channel - file name for the adiabatic shaped pulse using in first ZQF              [CHIRP]
;        smoothed chirp (low to high, 20% smoothing, 1000 points, 20KHz)
;spnam34 : f1 channel - file name for the adiabatic shaped pulse using in last ZQF               [CHIRP]
;        smoothed chirp (low to high, 20% smoothing, 1000 points, 20KHz)

;GRADIENT SHAPE
;gpnam1 : SMSQ10.100
;gpnam2 : SMSQ10.100

;GRADIENT STRENGTH
;gpz0 : first ZQF gradient          [3%]
;gpz1 : CTP gradient                [23%]
;gpz2 : spoil gradient              [79%]
;gpz10: last ZQF gradient           [4%]

;OTHER
;cnst2 : selected J(HX)(in Hz)
;cnst3 : 1 for AX/AX3 and 2 for AX2
;cnst6: bandwidth of TOCSY transfer (in Hz)
;cnst9: 1 (with DIPSI-2) / 0 (without DIPSI-2)
;cnst12: f1 channel - bandwidth for selective refocussing pulse (in Hz)
;cnst13: f1 channel - chemical shift for selective refocussing pulse (in ppm)
;cnst14: f2 channel - bandwidth for selective inversion pulse (in Hz)
;cnst15: f2 channel - chemical shift for selective inversion pulse (in ppm)
;cnst53 : GammaB1 of first adiabatic ZQF shaped pulse
;cnst54 : GammaB1 of last adiabatic ZQF shaped pulse
;l1   : loop for DIPSI cycle: ((p6*115.112) * l1) = mixing time
;ns: 16 * n, total number of scans: NS * TD0
;ds: 8
;MC2 : QF
```

6.3. Selective fluorine reverse INEPT (SRI) pulse program suitable for a TBI probe

```
; mSRI experiment
;
; Selective fluorine reverse INEPT (19F-1H correlation)
;   using selective 90 19F pulse for excitation
;   using PFG for CTP selection
;
; Developed by:
;   NMR Methodology Group
;   University of Manchester
; FESTA:
; L. Castanar, P. Moutzouri, T. Barbosa, C. Tormena, R. Rittner, A. Phillips, S. Coombes, M. Nilsson, G. A. Morris.
; Anal. Chem., 2018, 90, 8, 5445-5450
; DOI: 10.1021/acs.analchem.8b00753
;T. M. Barbosa, L. Castanar, P. Moutzouri, M. Nilsson, G. A. Morris, R. Rittner, C. F. Tormena
; Anal. Chem. 2020, 92, 2, 2224–2228
; DOI: 10.1021/acs.analchem.9b04924
;G. Dal Poggetto, J. V. Soares, C. F. Tormena
; Anal. Chem. 2020, 92, 20, 14047–1405
; DOI: 10.1021/acs.analchem.0c02976
;
;
; Options:
;		[cnst1 = 1] with 1H purge pulses before d1
;		[cnst1 = 0] without 1H purge pulses before d1
;
;
;$CLASS=HighRes
;$DIM=1D
;$TYPE=
;$SUBTYPE=
;$COMMENT=

#include <Avance.incl>
#include <Grad.incl>
#include <Delay.incl>
#include <De.incl>

;%%%%%%%%%% WaveMaker Parameters %%%%%%%%%%
;sp11:wvm:X_90_guass:f2 gaus90(cnst15 Hz, cnst13 ppm; NPOINTS=1000) ss=5 us;
"spoff11=0"
"d11=30m+1s/(cnst13)-1s/(cnst13)"
"d11=30m+1s/(cnst15)-1s/(cnst15)"
"d11=30m"

; PULSES
"p2=p1*2"
"p4=p3*2"

; DELAYS
"d2=1s/(4*cnst2)"
"d13=20u"
"d16=200u"
"d26=p16+d16"
"DELTA1=d2"
"DELTA2=d2-p16-d16"

; OTHER
"cnst30=(sfo2/sfo1)"
; If using heteronuclei with negative gyromagnetic ratio:
;"cnst30=-(sfo2/sfo1)"

"acqt0=0"

baseopt_echo
```

```
1 ze
 30m
2 30m
  d13 UNBLKGRAD

 if "cnst1 == 1"                          ;Purge pulses
   {
  d13 UNBLKGRAD
  d13 pl1:f1
  p16:gp20
  d16
  (p1 ph20):f1
  (p1 ph21):f1
  p16:gp21
  d16
  d13 BLKGRAD
   }
  else
   {
   }

  10u BLKGRAD
  50u LOCKH_OFF
  d1
  50u LOCKH_ON
  10u UNBLKGRAMP
  10u pl0:f2 pl1:f1

   (p11:sp11 ph1):f2
   (p2 ph2):f1
   DELTA1 pl2:f2 pl1:f1
   (p4 ph3):f2
   (p2 ph2):f1
   DELTA2
   p16:gp1
   d16

  (p3 ph4):f2
  p16:gp2
  d16
  (p1 ph5):f1

  p16:gp1*cnst30
  d16
  (p2 ph6):f1
  d26

  go=2 ph31
  30m mc #0 to 2 F0(zd)
  d13 BLKGRAD
exit

ph1=0 2 2 0
ph2=0
ph3=0
ph4=1
ph5=0
ph6=0
ph20=0
ph21=1
ph31=0 2 2 0

;POWER LEVEL
;pl0  : zero power (0 W)
;pl1  : f1 channel - power level for pulse (default)
```

;pl2 : f2 channel - power level for pulse (default)
;sp11 : f2 channel - power level of excitation shaped pulse

;PULSE DURATION
;p1 : f1 channel - 90 degree high power pulse
;p2 : f1 channel - 180 degree high power pulse
;p3 : f2 channel - 90 degree high power pulse
;p4 : f2 channel - 180 degree high power pulse
;p11: f2 channel - 90 degree low power shaped pulse
;   choose p11 according to desired selectivity

;GRADIENT DURATION
;p16: duration of CTP gradients   [1 ms]

;DELAY
;d1 : relaxation delay; 1-5 * T1
;d2 : 1/(4*J(XH))
;d16: delay for gradient recovery   [200 usec]

;PULSE SHAPE
;spnam11 : f2 channel - file name for the selective 90 excitation shaped pulse   [Gauss]

;GRADIENT SHAPE
;gpnam1 : SMSQ10.100
;gpnam2 : SMSQ10.100
;gpnam20: SMSQ10.100
;gpnam21: SMSQ10.100

;GRADIENT STRENGTH
;gpz1 : X-H CTP gradient   [47%]
;gpz2 : homospoil gradient   [37%]
;gpz20 : homospoil gradient purge pulses   [53%]
;gpz21 : homospoil gradient purge pulses   [59%]

;CONSTANTS
;cnst1 : 0 (--) / 1 (Purge pulses)
;cnst2 : J(HX)
;cnst13: f2 channel - chemical shift for selective X pulse (in ppm)
;cnst15: f2 channel - bandwidth for the selective refocussing pulse (in Hz)
;cnst30 : X/H gamma ratio

;OTHER
;ns: 2 * n, total number of scans: NS * TD0
;ds: 2
;set O2 on resonance to the 19F signal of interest

7. References

(1) Castañar, L.; Moutzouri, P.; Barbosa, T. M.; Tormena, C. F.; Rittner, R.; Phillips, A. R.; Coombes, S. R.; Nilsson, M.; Morris, G. A. FESTA: An Efficient Nuclear Magnetic Resonance Approach for the Structural Analysis of Mixtures Containing Fluorinated Species. *Anal. Chem.* **2018**, *90* (8), 5445–5450. https://doi.org/10.1021/acs.analchem.8b00753.

(2) Thrippleton, M. J.; Keeler, J. Elimination of Zero-Quantum Interference in Two-Dimensional NMR Spectra. *Angew. Chemie - Int. Ed.* **2003**, *42* (33), 3938–3941. https://doi.org/10.1002/anie.200351947.

(3) Gaussian 16, Revision C.01, M. J. Frisch, G. W. Trucks, H. B. Schlegel, G. E. Scuseria, M. A. Robb, J. R. Cheeseman, G. Scalmani, V. Barone, G. A. Petersson, H. Nakatsuji, X. Li, M. Caricato, A. V. Marenich, J. Bloino, B. G. Janesko, R. Gomperts, B. Mennucci, H. P. Hratchian, J. V. Ortiz, A. F. Izmaylov, J. L. Sonnenberg, D. Williams-Young, F. Ding, F. Lipparini, F. Egidi, J. Goings, B. Peng, A. Petrone, T. Henderson, D. Ranasinghe, V. G. Zakrzewski, J. Gao, N. Reg, G. Zheng, W. Liang, M. Hada, M. Ehara, K. Toyota, R. Fukuda, J. Hasegawa, M. Ishida, T. Nakajima, Y. Honda, O. Kitao, H. Nakai, T. Vreven, K. Throssell, J. A. Montgomery, Jr., J. E. Peralta, F. Ogliaro, M. J. Bearpark, J. J. Heyd, E. N. Brothers, K. N. Kudin, V. N. Staroverov, T. A. Keith, R. Kobayashi, J. Normand, K. Raghavachari, A. P. Rendell, J. C. Burant, S. S. Iyengar, J. Tomasi, M. Cossi, J. M. Millam, M. Klene, C. Adamo, R. Cammi, J. W.

Ochterski, R. L. Martin, K. Morokuma, O. Farkas, J. B. Foresman, and D. J. Fox, Gaussian, Inc., Wallingford CT, 2016.

(4) Barbosa, T. M.; Viesser, R. V.; Abraham, R. J.; Rittner, R.; Tormena, C. F. Experimental and Theoretical Evaluation of Trans-3-Halo-2-Hydroxy-Tetrahydropyran Conformational Preferences. Beyond Anomeric Interaction. *RSC Adv.* **2015**, *5* (45), 35412–35420. https://doi.org/10.1039/c5ra04968g.

(5) Fokt, I.; Szymanski, S.; Skora, S.; Cybulski, M.; Madden, T.; Priebe, W. D-Glucose- and d-Mannose-Based Antimetabolites. Part 2. Facile Synthesis of 2-Deoxy-2-Halo-d-Glucoses and -d-Mannoses. *Carbohydr. Res.* **2009**, *344* (12), 1464–1473. https://doi.org/10.1016/j.carres.2009.06.016.

(6) Ochterski, J. W. Thermochemistry in Gaussian. Gaussian, 2022. https://gaussian.com/wp-content/uploads/dl/thermo.pdf (accessed 2023-06-15).

(7) Guntert, P.; Schaefer, N.; Otting, G.; Wuthrich, K. POMA: A Complete Mathematica Implementation of the NMR Product-Operator Formalism. *J. Magn. Reson. Ser. A* **1993**, *101* (1), 103–105. https://doi.org/10.1006/jmra.1993.1016.
